# Mechanistic DFT Study of 1,3-Dipolar Cycloadditions of Azides with Guanidine

**DOI:** 10.3390/molecules28052342

**Published:** 2023-03-03

**Authors:** Ivana Antol, Zoran Glasovac, Davor Margetić

**Affiliations:** Ruđer Bošković Institute, Bijenička Cesta 54, HR-10002 Zagreb, Croatia

**Keywords:** 1,3-dipolar cycloadditions, DFT calculations, azides, guanidines, tetrazoles

## Abstract

Density functional calculations SMD(chloroform)//B3LYP/6-311+G(2d,p) were employed in the computational study of 1,3-dipolar cycloadditions of azides with guanidine. The formation of two regioisomeric tetrazoles and their rearrangement to cyclic aziridines and open-chain guanidine products were modeled. The results suggest the feasibility of an uncatalyzed reaction under very drastic conditions since the thermodynamically preferred reaction path (a), which involves cycloaddition by binding the carbon atom from guanidine to the terminal azide nitrogen atom, and the guanidine imino nitrogen with the inner N atom from the azide, has an energy barrier higher than 50 kcal mol^−1^. The formation of the other regioisomeric tetrazole (imino nitrogen interacts with terminal N atom of azide) in direction (b) can be more favorable and proceed under milder conditions if alternative activation of the nitrogen molecule releases (e.g., photochemical activation), or deamination could be achieved because these processes have the highest barrier in the less favorable (b) branch of the mechanism. The introduction of substituents should favorably affect the cycloaddition reactivity of the azides, with the greatest effects expected for the benzyl and perfluorophenyl groups.

## 1. Introduction

Guanidines are organic compounds that possess very interesting physico-chemical properties, such as very high basicity [1,2] and biological activity [3]. Their chemical behavior is extensively studied and guanidines have found applications in various fields, such as catalysis [4], coordination chemistry [5], supramolecular chemistry [6], medicinal chemistry [7,8] and chemical sensoring [9,10]. Whereas this versatile functional group undergoes numerous chemical reactions, guanidines are rarely involved in cycloaddition reactions [11], either in Diels–Alder or dipolar cycloadditions. Moreover, there are scarce reports on the cycloadditions where the imino bond of guanidines undertakes the role of dipolarophile with the formation of a new heterocyclic ring. An example is the formal 1,3-dipolar cycloaddition to guanidine, where aryldiazonium salts reacted with guanidines [12], NIS/DBU catalyzed to obtain aminotetrazoles—a multistep process involving nucleophilic addition/cyclization. The mechanistically similar formal 1,3-dipolar reaction of aryldiazonium salts and amidines facilitated by K_2_CO_3_/I_2_ provided tetrazole [13]. Tetrazoles are of great interest due to their various biological activities [14], and are constituents of many active pharmaceutical ingredients [15].

Different synthetic strategies are employed for the synthesis of tetrazoles [16]; however, the direct addition of azide to guanidine was not utilized. Realizing that this type of reaction has not been not theoretically studied to date, and in continuation of our interest in cycloaddition reactions [17,18] and study of the effects of the substitution of dienes with guanidines [19], we conducted this computational study to address several scientific questions: What is the reactivity of guanidine in 1,3-dipolar cycloadditions, and what is the regioselectivity of this reaction? This study aims to establish the cycloaddition (dipolarophilic) properties of imine bond in guanidine and the regioselectivity of the formation of tetrazoles. In addition, the stability of the initially formed cycloadducts and other conceivable reaction paths leading to other products needs to be considered.

## 2. Results and Discussion

**Figure 1:**
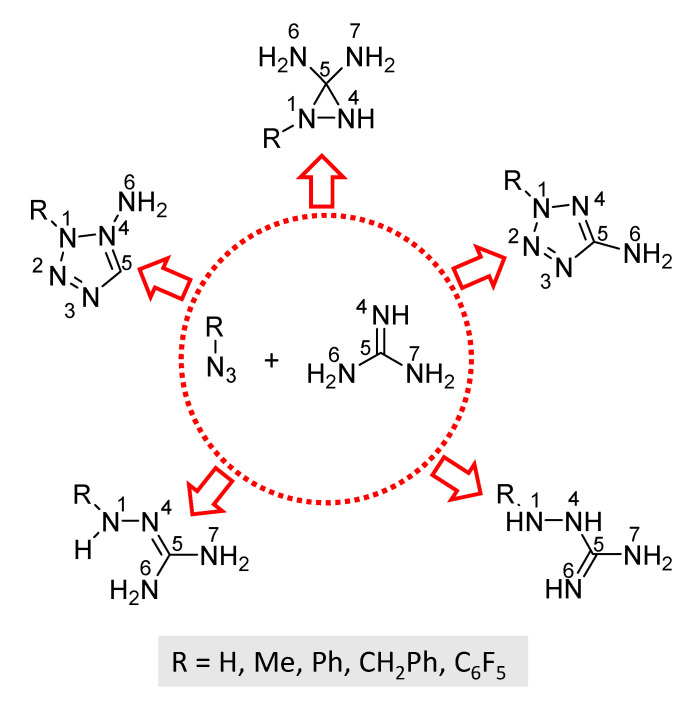
Model reaction system and possible products.

A theoretical investigation of the 1,3-dipolar cycloaddition reactivity of guanidines was carried out on the model reaction system depicted in Figure 1. Mechanistic paths for the formation of two regioisomeric tetrazoles, cyclic aziridines, and open chain guanidines were modeled for all intermediates and transition states (TSs).

**Figure 2:**
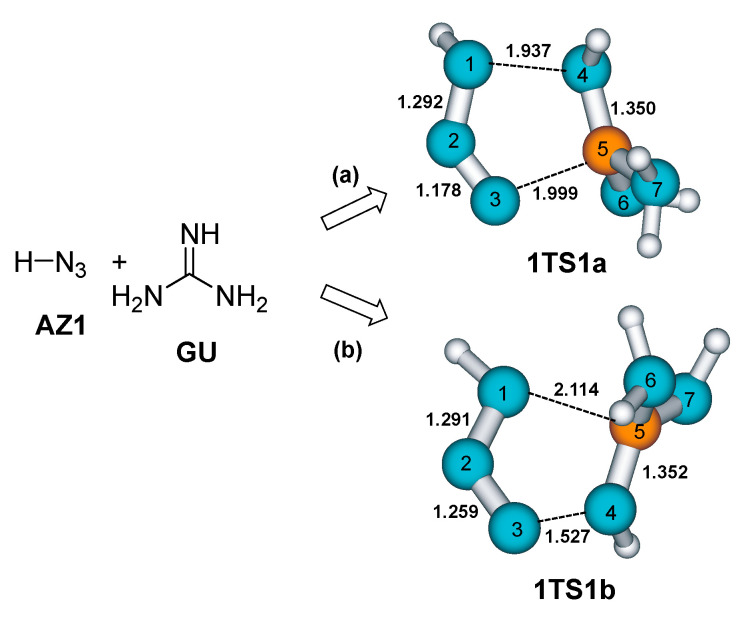
Transition state structures (bond lengths are given in Å) for two different modes of azide addition to the guanidine C=N bond. (**a**) addition mode (a); (**b**) addition mode (b).

The theoretical study of the reaction mechanism of the 1,3-dipolar cycloaddition of azide to the guanidine C=N double bond was initiated with the SMD(chloroform)//B3LYP/6-311+G(2d,p) calculations of unsubstituted derivatives: azide **AZ1** (hydrazoic acid) and guanidine **GU** (Figure 2). The B3LYP functional [20,21] was previously used for successful regioselectivity investigations of similar thermal, uncatalyzed 1,3-dipolar cycloadditions [22,23,24,25], including azide cycloaddition to acetylenes [26]; and azide cycloaddition to nitriles forming tetrazoles [27], which are early applications of DFT methods to this kind of reaction.

The addition of an azide functional group to a C=N double bond can proceed in two ways, leading to two different regioisomers: (a) the carbon atom from guanidine binds to the terminal azide N3 atom, and the guanidine imino nitrogen interacts with the N1 atom from the azide; in the second reaction mode (b), interacting atoms are reversed. Imino nitrogen (N4) forms a bond to the N3 end of the azide group while the carbon atom interacts with the N1 azide nitrogen. In both regioisomeric approaches, the formation of new C-N and N-N bonds is a concerted process, but due to the asymmetry of the reagents, the transition structures are partly asymmetric (Figure 2). The bond lengths of new forming bonds are in good accordance with the reactions of azides to enamines [28], alkenes (distances 2.02–2.27 Å) [29], or to nitriles (distances 1.92–2.07 Å; regioisomers’ energy difference is smaller—ΔΔ*H*_act_ is about 5–6 kcal mol^−1^) [27].

**Figure 3:**
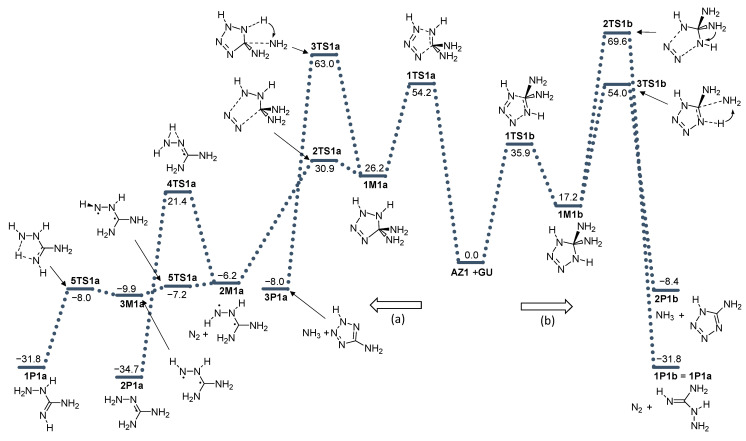
Energy profile for the addition of azide **AZ1** with guanidine **GU**. Energies relative to reactants are given in kcal mol^−1^. (**a**) and (**b**) denote two modes of azide addition to **GU**.

The direction (b) is energetically significantly more favorable, by 18.3 kcal mol^−1^ (*E*_rel_ (**1TS1b**) = 35.9 kcal mol^−1^ vs. *E*_rel_ (**1TS1a**) = 54.2 kcal mol^−1^) (Figure 3). Both processes are endothermic and the 5,5-diamino-1,4(or 2)-dihydro-1,2,3,4-tetrazole products are less stable than the reactant by 26.2 and 17.2 kcal mol^−1^ for (a) and (b) directions of the reaction, respectively. It is known that the triazolines formed by the analogous cycloaddition of azides to alkenes are unstable and convert to aziridines, amines, or pyrazolines [15,30,31]. Hence, we assumed that in the case of additions to guanidine, the resulting tetrazole products would also be thermodynamically unstable and subject to similar rearrangements. Two transition state structures for the release of N_2_ and two TS structures for the release of NH_3_ molecules were found on the potential energy surface. In the (a) direction, the release of N_2_ is far more probable than the release of ammonia because the energy barrier is extremely small, only 4.7 kcal mol^−1^. The resulting diradical intermediate is even more easily rearranged to form thermodynamically stable amino-guanidine end-products (Figure 3). In the (b) reaction direction, the release of ammonia is a more favorable process than the release of N_2_, but it should be borne in mind that both processes are significantly more energy-demanding than identical processes for direction (a).

**Figure 4:**
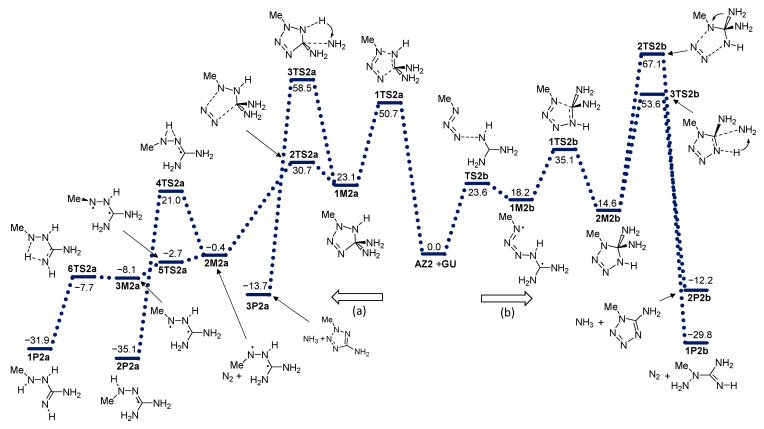
Energy profile for the addition of azide **AZ2** with guanidine **GU**. Energies relative to reactants are given in kcal mol^−1^. (**a**) and (**b**) denote two modes of azide addition to **GU**.

**Figure 5:**
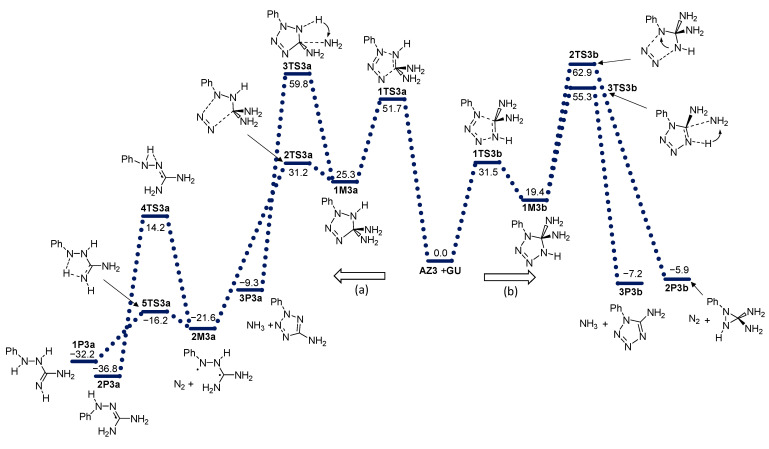
Energy profile for the addition of azide **AZ3** with guanidine **GU**. Energies relative to reactants are given in kcal mol^−1^. (**a**) and (**b**) denote two modes of azide addition to **GU**.

**Figure 6:**
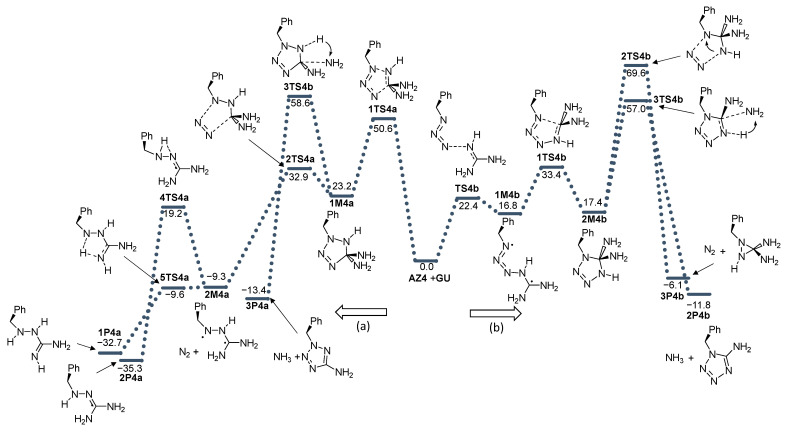
Energy profile for the addition of azide **AZ4** with guanidine **GU**. Energies relative to reactants are given in kcal mol^−1^. (**a**) and (**b**) denote two modes of azide addition to **GU**.

**Figure 7:**
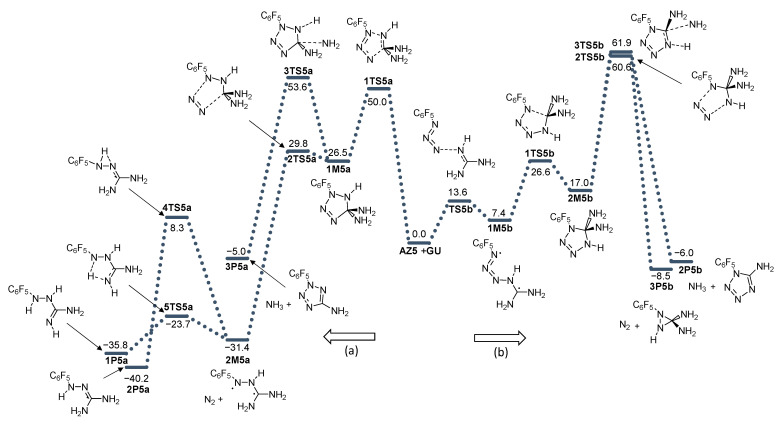
Energy profile for the addition of azide **AZ5** with guanidine **GU**. Energies relative to reactants are given in kcal mol^−1^. (**a**) and (**b**) denote two modes of azide addition to **GU**.

The effect on the reactivity of azide substitution obtained by different functional groups was examined by the introduction of methyl (**AZ2**), phenyl (**AZ3**), benzyl (**AZ4**), and perfluorophenyl (**AZ5**) groups to the azide functionality. The energy profiles of these reactions are given in Figure 4, Figure 5, Figure 6 and Figure 7.

**Figure 8:**
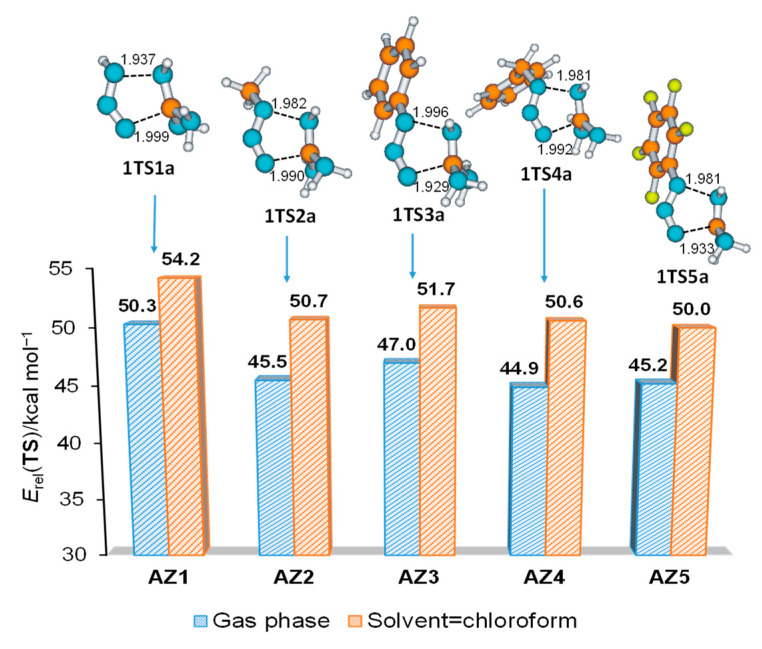
Relative energies (*E*_rel_/kcal mol^−1^) of transition state structures for cycloaddition of different azides **AZ1**–**AZ5** to guanidine **GU** in direction (a).

The replacement of the azide hydrogen atom with different functional groups did not lead to a significant change in the geometry of the TS structures for cycloaddition when the carbon atom approached the azide terminal N3 atom (a reaction direction) (Figure 8). The synchronous cycloaddition process is energetically more favorable for 2.5–4.2 kcal mol^−1^ compared to hydrazoic acid **AZ1**. The lowest energy barrier was computed for the reaction of guanidine with **AZ5** (introduction of the perfluorophenyl group). Additionally, the benzoyl group is preferable when compared to phenyl, which is in good accordance with the larger cycloaddition reactivity of benzyl compared to phenyl azide [32].

After cyclization, in all cases, the N_2_ release and formation of stable products are expected. Activation energies for the elimination of N_2_ are smaller for phenyl and C_6_F_5_ substituents in comparison with an unsubstituted derivative (5.9 and 3.3 kcal mol^−1^, respectively), whereas the substitution by methyl and benzyl raises the barrier (7.6 and 9.7 kcal mol^−1^). The activation energies for further diradical rearrangement are higher for phenyl and C_6_F_5_ substituents (5.4 and 7.7 kcal mol^−1^) than for other substituents and could be associated with the better stabilization of aniline radicals [33]. Similar to unsubstituted reactants, the alternative process of deamination and amino-tetrazole ring formation is less favorable. For the deamination process, activation energies are in the range between 34.5 and 35.4 kcal mol^−1^ for all substituents, with the exception of phenyl, where *E*_a_ is smaller than 33.1 kcal mol^−1^.

There are some mechanistic differences in direction (b) in comparison to **AZ1**. The synchronous formation of N3-N4 and N1-C5 bonds occurs exclusively in the reaction of guanidine with phenylazide **AZ3** (this is similar to **AZ1**). The relative energy of the transition structure is lower by 4.4 kcal mol^−1^ compared to the corresponding TS structure in the reaction of unsubstituted azide. The resulting adduct **1M3b** is less stable than the unsubstituted adduct by 2.2 kcal mol^−1^.

**Figure 9:**
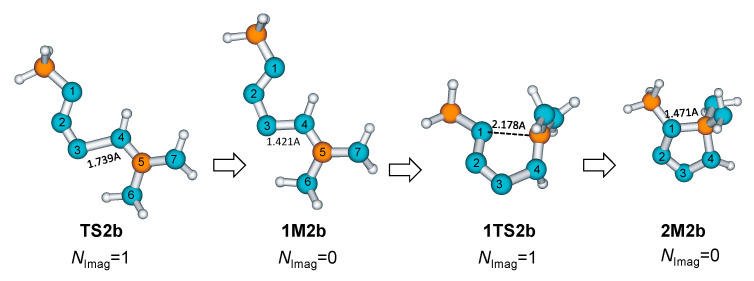
Stationary points on the potential energy surface along the reaction pathway describing the cycloaddition of methyl azide **AZ2** to guanidine **GU** in direction (b).

For other azides, **AZ2**, **AZ4,** and **AZ5**, the formation of diaminotetrazoles is a two-step process involving two TSs on the potential energy surface along the reaction pathway. The first (**TSNb**, N = 2,4,5) is a TS structure of a nucleophilic attack that creates a bond between the terminal azide nitrogen N3 and guanidine imino nitrogen N4 and the formation of intermediate **1MNb,** N = 2,4,5. The activation energies for initial nucleophilic attack are significantly lower than for synchronous TS and the formation of diaminotetrazole, which indicates that the nucleophilicity properties of azides are increased by methyl, benzyl, and C_6_H_5_ groups [34]. In the second stage of the reaction, the five-membered ring is closed by coupling the N1 azide nitrogen with a carbon atom from the guanidine group via a **1TSNb**, N = 2,4,5 structures. Examples of such structures are shown for **AZ2** in Figure 9.

**Figure 10:**
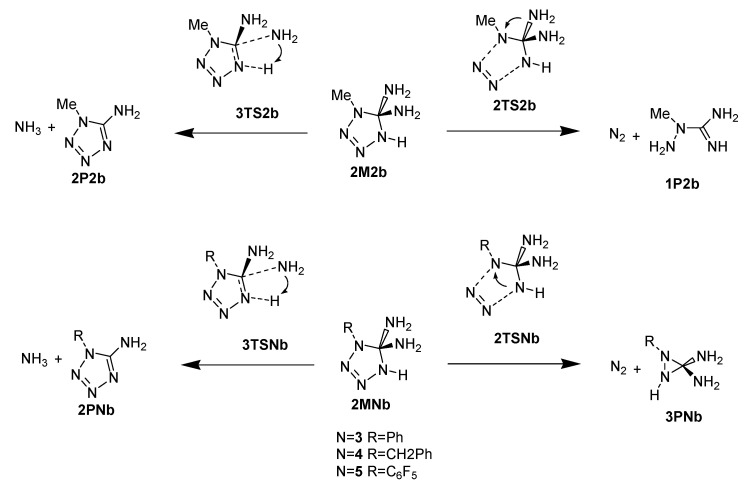
Schematic representation of the rearrangement of adducts from the addition of azide to guanidine in the direction (b).

The nitrogen released from the formed diaminotetrazole adducts during the reaction path (b) goes through **3TS** transition state structures with very high energies relative to the reactant: 67.1 (methyl group, **3TS2b**), 62.9 (phenyl group, **3TS3b**), 69.6 (benzyl group, **3TS4b**), and 60.6 kcal mol^−1^ (perfluorophenyl group **3TS5b**). IRC calculations showed that in the case of an adduct with a methyl group at the N1 position at the exit of N_2_, the amino group migrates from the C atom to the N1 position and a stable *N’,N’*-aminomethylguanidine is formed (**1P2b**). In adducts with larger substituents (phenyl, benzyl, and parafluorophenyl), migration of the amino group was not observed, but the closure of the three-membered aziridine ring occurred (Figure 10).

The deamination of diaminotetrazole **2M** structures goes through transition states **3TS** before reaching the final product **2P** (Figure 10). In the case of **AZ1**-**AZ4,** this process is kinetically more favorable than nitrogen release. It should be emphasized, however, that the energy barriers for this deamination (direction b) are also very high: 53.6 (-Me), 55.3 (-Ph), and 57.0 (-Bn). For **AZ5**, the energy of **3TS5b** is 61.9 kcal mol^−1^ less favorable than the nitrogen release by 1.3 kcal mol^−1^.

**Figure 11:**
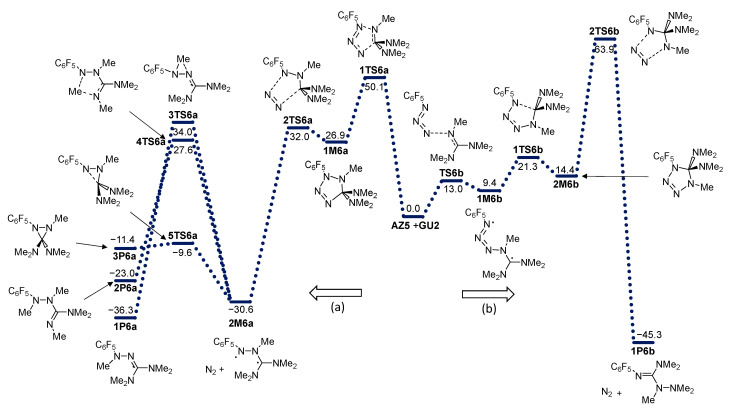
Energy profile for the addition of perfluorophenyl azide **AZ5** with permethylated guanidine **GU2**. Energies relative to reactants are given in kcal mol^−1^. (**a**) and (**b**) denote two modes of azide addition to **GU**.

**Figure 12:**
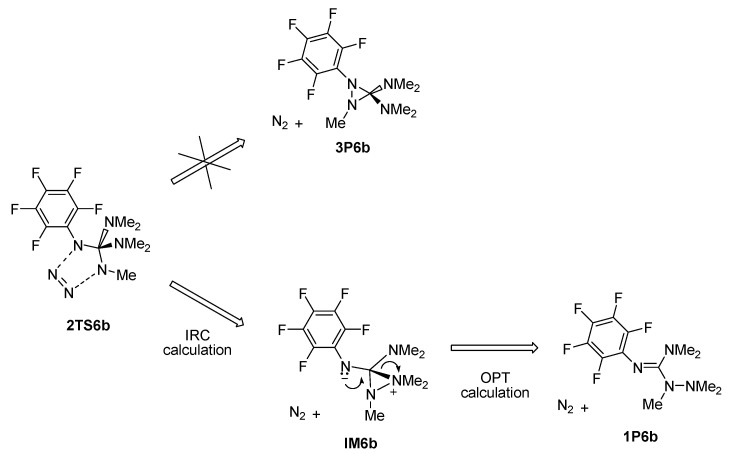
Schematic representation of the rearrangement of adducts from the addition of azide **AZ5** to guanidine **GU2** in the (b) direction.

The effect of substitution on the guanidine moiety was investigated by observing the reaction of **AZ4** with permethylated guanidine **GU2**. The energy profile is given in Figure 11. In brief, the cycloaddition part of the energy profile is very similar to that of unsubstituted guanidine (a comparison of energy profiles is provided in Figure 10 and Figure 11), which means that the direction (a) is less favorable and energetically more demanding than direction (b). The energy barrier for cycloaddition in the (a) direction is almost identical in the case of **GU** and **GU2**. In the (b) direction, all stationary points have a slightly lower energy in the case of **GU2**. The N_2_ release, similar to that found for **GU**, is more favorable in the (a) direction than the (b) direction. When the same behavior as before is observed in the (a) direction, a stable diradical intermediate is formed, which is further rearranged or cyclized into final products. The rearrangement is more energetically demanding in the case of **GU2,** because the radical center needs to pick up Methyl instead of H, and more energy is needed for this. Cyclization results in a less stable cyclic product, probably due to steric hindrance. The IRC calculation shows that, in the (b) direction from the **2TS6b** structure, the expected cyclic product **3P6b** is not formed (we expected this because, in the case of **GU**, **3P5b** was formed as a stable product). Instead, IRC leads to an intermediate structure for **IM6b**. An attempt to optimize this structure without an energy barrier leads to the final product, **1P6b** (see Figure 12).

To summarize, the reaction energy profiles from this computational study indicate that the product of cycloaddition formed through direction (b) should be kinetically favored; however, further rearrangements (elimination) of initial cycloadducts can more easily energetically proceed through direction (a). Hence, the formation of aminoguanidine from direction (a) is a favorable process.

## 3. Materials and Methods

The B3LYP/6-311+G(2d,p) method [18,19] was used to study the mechanism of azide addition to guanidine. Structures of reactants, transition states, and products were optimized without any symmetry constraints in the gas phase. Vibrational analysis was performed, and all structures were characterized either as minima without imaginary frequencies or as transition state structures with one imaginary frequency. The total energy of each stationary point on the surface of the potential energy was corrected by unscaled ZPV energy. The association of products with reactants via transition structures was confirmed by IRC calculations. All energy profiles are given in Figures S1–S6 in Supplementary Materials. To better mimic the usual experimental conditions, solvent effects were included as single-point calculations on optimized PES stationary points using the SMD method [35] and chloroform as solvent. Full population analysis with Hirshfeld [36,37] and the NBO [38] option was performed on reactants and selected TS structures. Partial atomic charges (Hirshfeld, NBO and Mulliken) of selected atoms are collected in Table S2. The Gaussian09 [39] software package was used to perform quantum-mechanical calculations, and the initial structures were generated using the Molden package [40].

Chloroform molecule is a very weak H-bonding acceptor and donor; therefore, strong explicit interactions with solute are not expected. Nevertheless, the calculations with one explicitly added CHCl_3_ molecule were undertaken for the reaction of **AZ3** with guanidine **GU** in the (a) direction. The results are listed in Supplementary Materials (Tables S3 and S5, Figure S7). It was shown that the energy profile was not altered when compared to previous results. Relative energies of all stationary points, with respect to reactants (TS as well as minima), were ca. 1.1–3.1 kcal mol^−1^ higher. It can be concluded that potential energy barriers were not substantially changed upon complexation with CHCl_3_ molecule. There is no reason to expect that the effect of CHCl_3_ complexation would be different for other azides studied in this paper.

## 4. Conclusions

The results of modeling the reaction pathways of the 1,3-dipolar cycloaddition of azides to guanidine suggest the feasibility of an uncatalyzed reaction, but under very drastic conditions. Regardless of the substituent that is present on azide, the formation of the aminoguanidine derivative by reaction path (a) is the thermodynamically most preferred process and, subject to high-temperature conditions, the expected product.

The reaction between azide and guanidine involves several steps, including the formation of different tetrazole intermediates and their rearrangements. It is interesting to note that the more favorable formation of the tetrazole intermediate is in the kinetically less favorable branch of the putative mechanism direction (b). This suggests the possibility of obtaining less thermodynamically favorable products under milder conditions but with alternative activation or release of the nitrogen molecule (e.g., photochemical activation) or deamination, because these processes have the highest barrier in the less favorable branch of the mechanism.

The introduction of azide substituents should favorably affect the cycloaddition reactivity of the azides, with the greatest effects being expected for the benzyl and perfluorophenyl groups.

## Data Availability

Not applicable.

## Supplementary Materials

The following supporting information can be downloaded at: www.mdpi.com/article/10.3390/molecules28052342/s1, Figures S1–S6: Potential energy profiles for cycloadditions in the gas phase; Figure S7: Stationary points along the reaction path with solvent explicitly included; Tables S1–S3: Energies, selected partial atomic charges, charge analysis and coordinates of all structures associated with this article.
