# Supplementary material for: Mechanistic DFT Study of 1,3-Dipolar Cycloadditions of Azides with Guanidine
Source: Molecules. 2023 Mar 3;28(5):2342. doi: 10.3390/molecules28052342 (PMC10004754; doi:10.3390/molecules28052342)
Supplement: Supplementary file 1 [file molecules-28-02342-s001.zip › molecules-2222332-supplementary.pdf]

Supplementary Material

for

Content:

1. Potential energy profiles for cycloadditions in the gas phase 2

2. Energies and partial atomic charges 5

3. Explicit solvation with one $CHCl_3$ molecule 11

4. Coordinates of all structures associated with this article 12

1. Potential energy profiles for cycloadditions in the gas phase

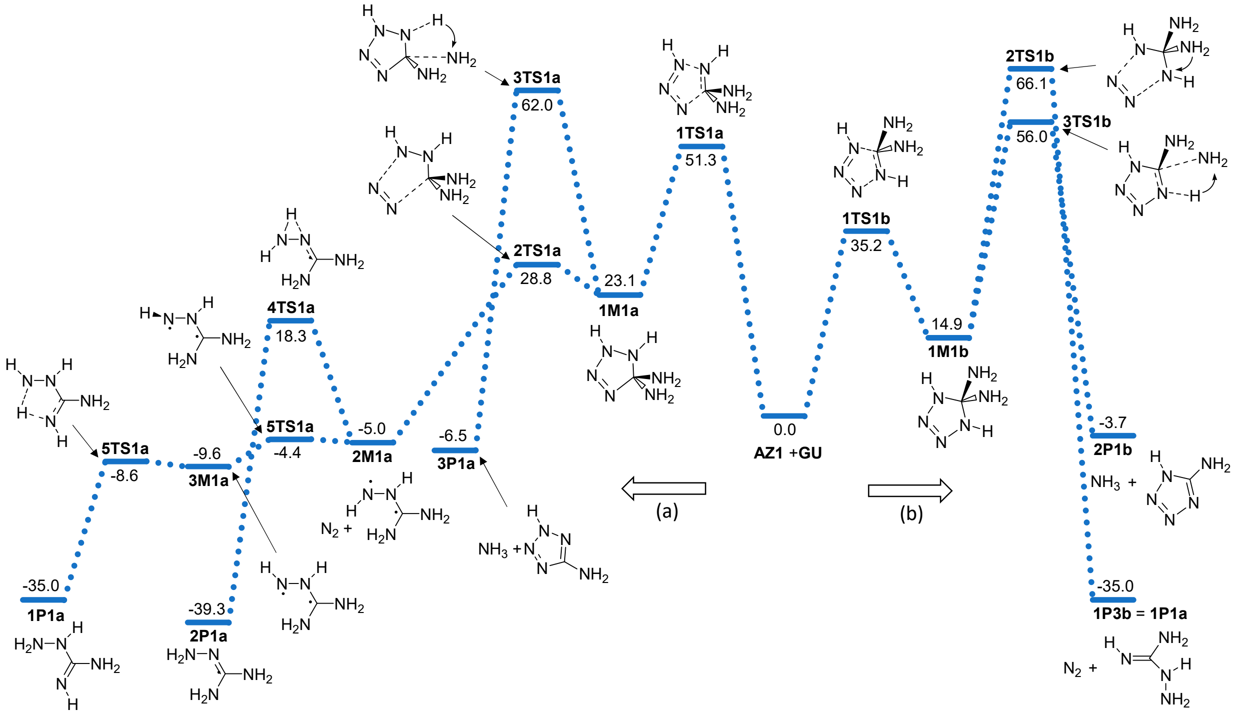

Figure S1. Energy profile for the addition of azide **AZ1** with guanidine **GU**. Energies relative to reactants are given in kcal mol^{-1}.

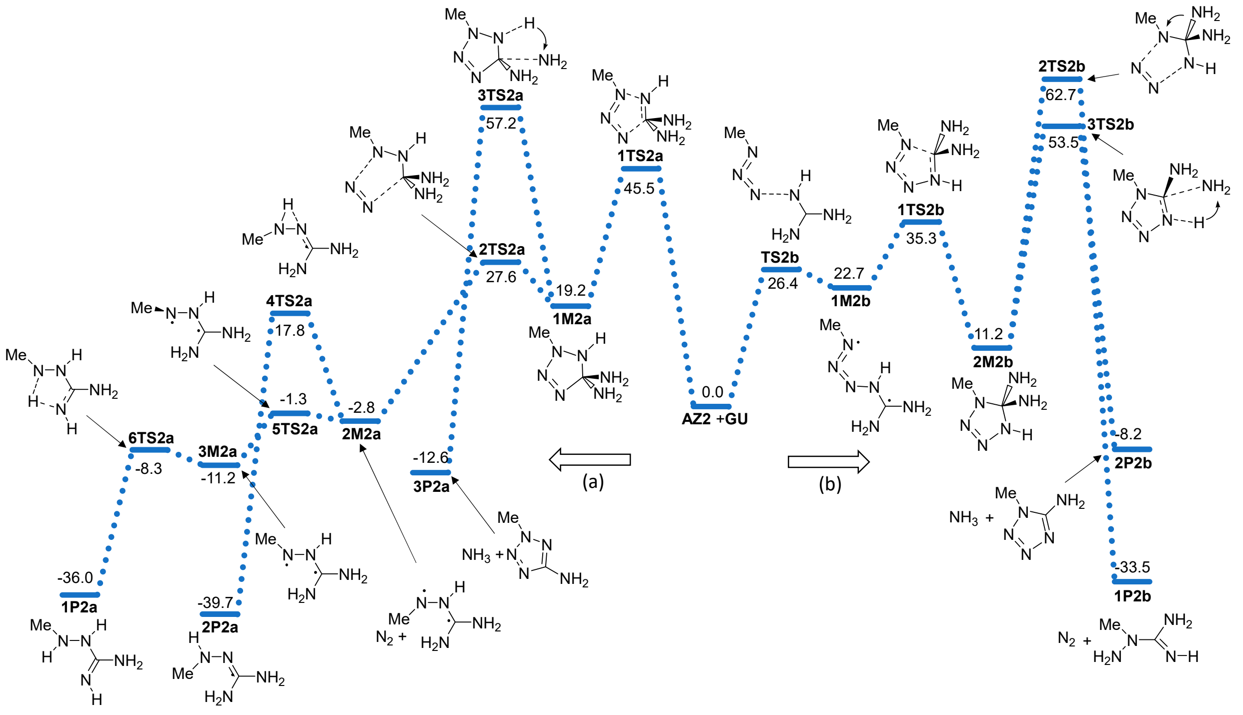

Figure S2. Energy profile for the addition of azide **AZ2** with guanidine **GU**. Energies relative to reactants are given in kcal mol^{-1}.

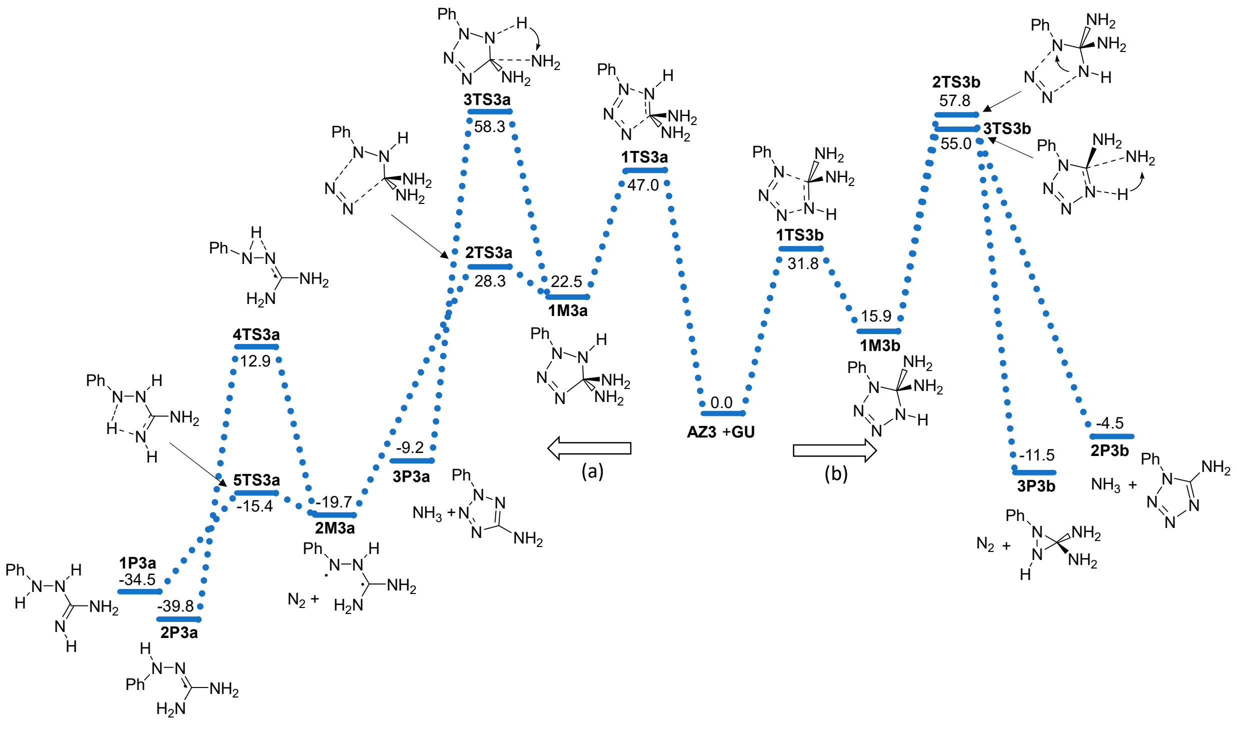

Figure S3. Energy profile for the addition of azide **AZ3** with guanidine **GU**. Energies relative to reactants are given in kcal mol^{-1}.

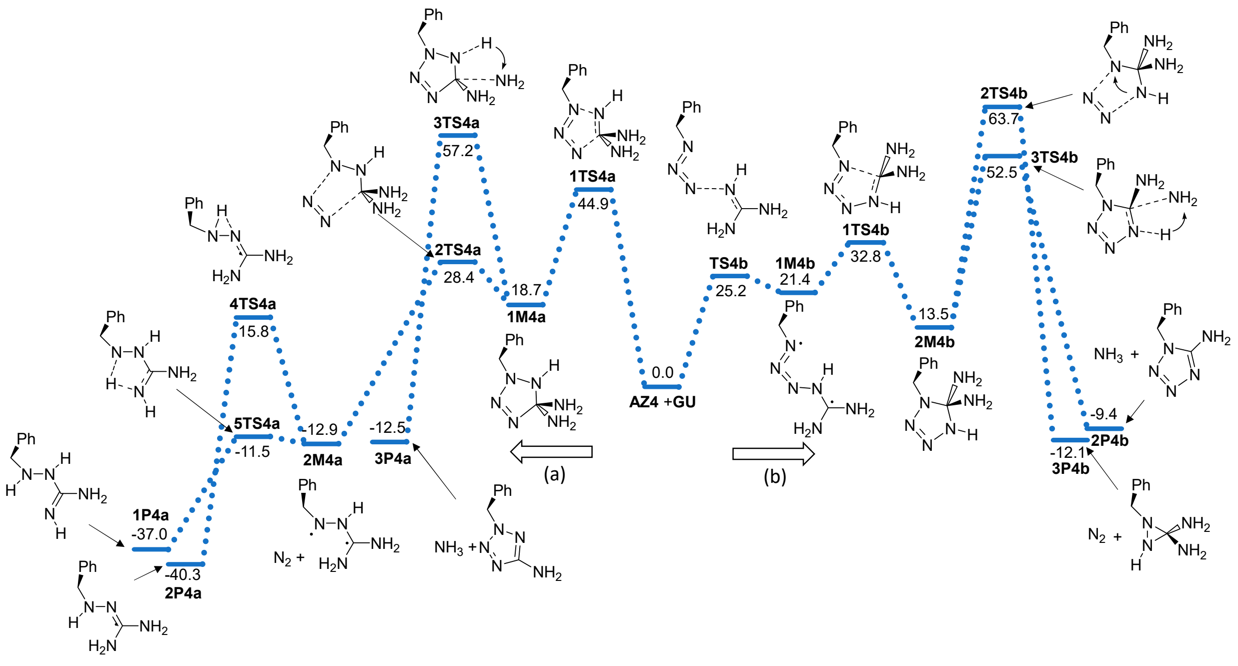

Figure S4. Energy profile for the addition of azide **AZ4** with guanidine **GU**. Energies relative to reactants are given in kcal mol^{-1}.

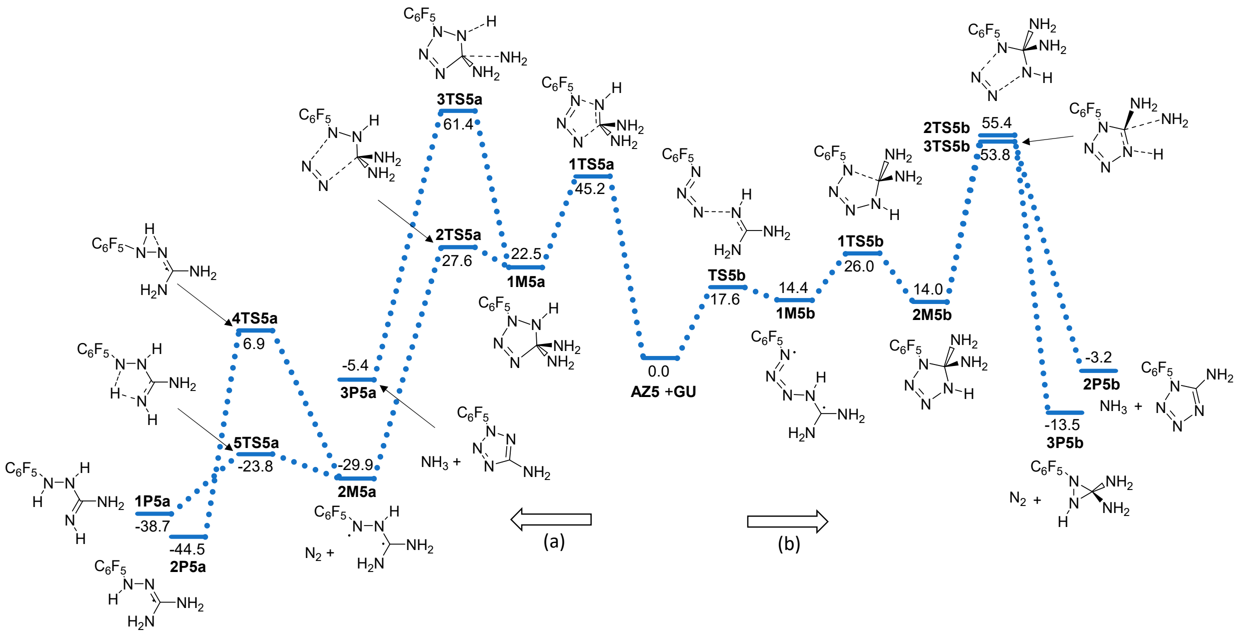

Figure S5. Energy profile for the addition of azide **AZ5** with guanidine **GU**. Energies relative to reactants are given in kcal mol^{-1}.

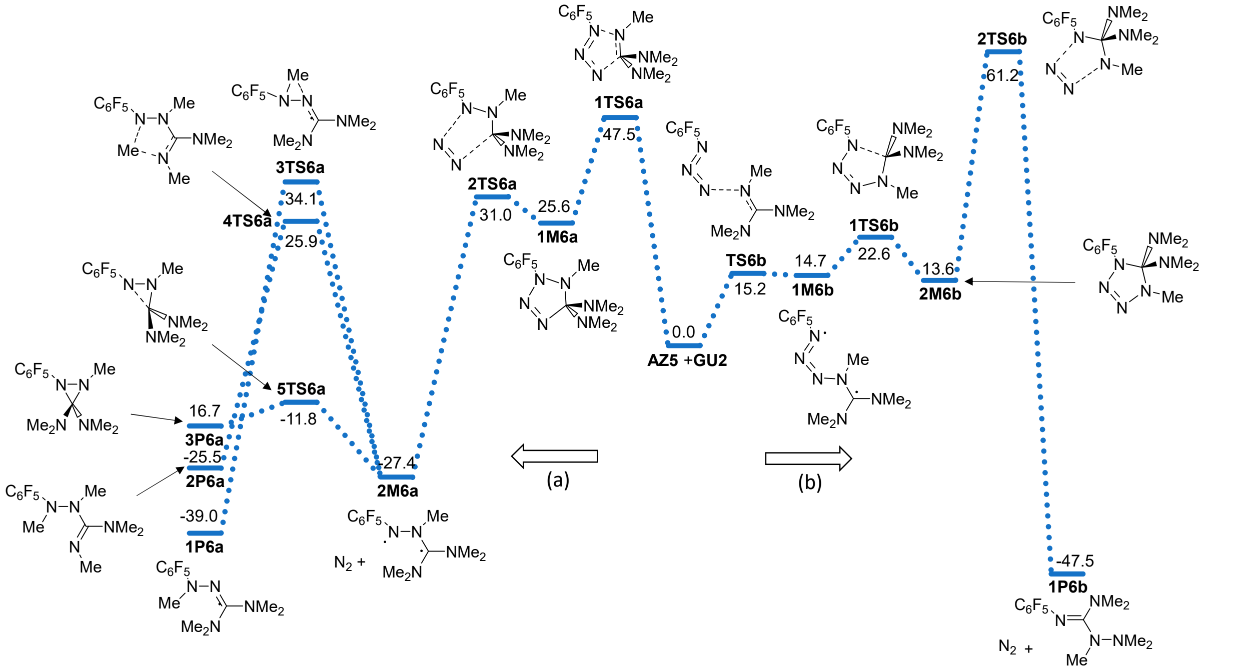

Figure S6. Energy profile for the addition of azide **AZ5** with guanidine **GU2**. Energies relative to reactants are given in kcal mol^{-1}.

2. Energies and partial atomic charges

Table S1. Energies, zero point vibrational energies (ZPVE), relative energies for all structures optimized at B3LYP/6-311+G(2d,p) level of theory in the gas phase using Integral=ultrafine option and energies and relative energies from single point SMD calculations with chloroform solvent. Imaginary frequencies are added in parentheses.

Structure	Gas Phase				Solvent: $CHCl_3$	
	Energy/a.u.	ZPVE/a.u.	N_{imag} (cm^{-1})	E_{rel}/kcal mol^{-1}	Energy/a.u.	E_{rel}/kcal mol^{-1}
**AZ1**	-164.84128	0.02133	0	-	-164.84084	-
**AZ2**	-204.15952	0.05010	0	-	-204.16012	-
**AZ3**	-395.95448	0.10299	0	-	-395.95996	-
**AZ4**	-435.27221	0.13117	0	-	-435.28038	-
**AZ5**	-892.26307	0.06255	0	-	-892.26407	-
**N_2**	-109.56348	0.00555	0	-	-109.55829	-
**NH_3**	-56.58384	0.03423	0	-	-56.58795	-
**$NHMe_2$**	-135.21245	0.09199	0	-	-135.21776	-
**GV**	-205.45032	0.07575	0	-	-205.46719	-
**GV2**	-402.01575	0.21408	0	-	-402.02883	-
**1TS1a**	-370.21345	0.09907	1 (-623.3)	50.3	-370.22363	54.2
**1M1a**	-370.26133	0.10369	0	23.1	-370.27287	26.2
**2TS1a**	-370.24969	0.10106	1 (-356.1)	28.8	-370.26284	30.9
**2M1a**	-260.73553	0.09103	0	-5.0	-260.75917	-6.2
**4TS1a**	-260.69378	0.08637	1 (-1520.0)	18.3	-260.71055	21.4
**2P1a**	-260.79196	0.09282	0	-39.3	-260.80639	-34.7
**5TS1a**	-260.73355	0.08999	1 (-325.6)	-4.4	-260.75966	-7.2
**3M1a**	-260.74285	0.09092	0	-9.6	-260.76488	-9.9
**6TS1a**	-260.73753	0.08725	1 (-934.3)	-8.6	-260.75829	-8.0
**1P1a**	-260.78493	0.09261	0	-35.0	-260.80151	-31.8
**3TS1a**	-370.19300	0.09730	1 (-1754.8)	62.0	-370.20792	63.0
**3P1a**	-313.71940	0.06414	0	-6.5	-313.73418	-8.0
**1TS1b**	-370.23876	0.10027	1 (-283.3)	35.2	-370.25408	35.9
**1M1b**	-370.27390	0.10315	0	14.9	-370.28672	17.2
**2TS1b**	-370.18584	0.09669	1 (-466.7)	66.1	-370.19676	69.6
**1P1b**	-260.78493	0.09261	0	-35.0	-260.80152	-31.8
**3TS1b**	-370.20235	0.09709	1 (-1702.9)	56.0	-370.22200	54.0
**2P1b**	-313.71422	0.06344	0	-3.7	-313.73402	-8.4
**1TS2a**	-409.53868	0.12724	1 (-547.7)	45.5	-409.54782	50.7
**1M2a**	-409.58434	0.13098	0	19.2	-409.59566	23.1
**2TS2a**	-409.56826	0.12827	1 (-383.9)	27.6	-409.58078	30.7
**2M2a**	-300.04956	0.11903	0	-2.8	-300.06836	-0.4
**4TS2a**	-300.01136	0.11368	1 (-1489.7)	17.8	-300.02886	21.0
**2P2a**	-300.11016	0.12079	0	-39.7	-300.12539	-35.1
**5TS2a**	-300.04561	0.11743	1 (-195.8)	-1.3	-300.07047	-2.7
**3M2a**	-300.06240	0.11842	0	-11.2	-300.08010	-8.1

**6TS2a**	-300.05352	0.11419	1 (-840.8)	-8.3	-300.07517	-7.7
**1P2a**	-300.10429	0.12086	0	-36.0	-300.12040	-31.9
**3TS2a**	-409.51779	0.12497	1 (-1770.3)	57.2	-409.53321	58.5
**3P2a**	-353.04640	0.09188	0	-12.6	-353.06143	-13.7
**1TS2b**	-409.55486	0.12713	1 (-189.0)	35.3	-409.57267	35.1
**2M2b**	-409.59748	0.13133	0	11.2	-409.60950	14.6
**TS2b**	-409.56809	0.12611	1 (-392.7)	26.4	-409.58992	23.6
**1M2b**	-409.57597	0.12814	0	22.7	-409.60056	18.2
**2TS2b**	-409.50750	0.12350	1 (-291.6)	62.7	-409.51805	67.1
**1P2b**	-300.09951	0.12008	0	-33.5	-300.11635	-29.8
**3TS2b**	-409.52296	0.12428	1 (-1782.0)	53.5	-409.54032	53.6
**2P2b**	-353.03883	0.09134	0	-8.2	-353.05846	-12.2
**1TS3a**	-601.33091	0.17979	1 (-512.2)	47.0	-601.34585	51.7
**1M3a**	-601.37490	0.18324	0	21.6	-601.39132	25.3
**2TS3a**	-601.36213	0.18123	1 (-285.7)	28.3	-601.37993	31.2
**2M3a**	-491.87107	0.17156	0	-19.7	-491.90170	-21.6
**5TS3a**	-491.85971	0.16706	1 (-1225.2)	-15.4	-491.88858	-16.2
**1P3a**	-491.89574	0.17268	0	-34.5	-491.91959	-32.2
**4TS3a**	-491.81398	0.16636	1 (-1622.3)	12.9	-491.83932	14.2
**2P3a**	-491.90459	0.17300	0	-39.8	-491.92733	-36.8
**3TS3a**	-601.31073	0.17765	1 (-1745.7)	58.3	-601.33070	59.8
**3P3a**	-544.83531	0.14428	0	-9.2	-544.85375	-9.3
**1TS3b**	-601.35598	0.18063	1 (-143.0)	31.8	-601.37888	31.5
**1M3b**	-601.38388	0.18312	0	15.9	-601.40059	19.4
**2TS3b**	-601.31204	0.17802	1 (-310.6)	57.8	-601.32622	62.9
**3P3b**	-491.85897	0.17259	0	-11.5	-491.87768	-5.9
**3TS3b**	-601.31591	0.17745	1 (-1714.1)	55.0	-601.33775	55.3
**2P3b**	-544.82758	0.14398	0	-4.5	-544.85017	-7.2
**1TS4a**	-640.65281	0.20873	1 (-544.0)	44.9	-640.66870	50.6
**1M4a**	-640.69825	0.21238	0	18.7	-640.71610	23.2
**2TS4a**	-640.67988	0.20956	1 (-388.5)	28.4	-640.69782	32.9
**2M4a**	-531.17822	0.19997	0	-12.9	-531.20264	-9.3
**5TS4a**	-531.17116	0.19551	1 (-882.4)	-11.3	-531.19879	-9.6
**1P4a**	-531.21862	0.20205	0	-37.0	-531.24212	-32.7
**4TS4a**	-531.12753	0.19504	1 (-1514.6)	15.8	-531.15242	19.2
**2P4a**	-531.22392	0.20205	0	-40.3	-531.24624	-35.3
**3TS4a**	-640.63068	0.20626	1 (-1762.8)	57.2	-640.65354	58.6
**3P4a**	-584.15903	0.17309	0	-12.5	-584.18143	-13.4
**1TS4b**	-640.67258	0.20928	1 (-132.2)	32.8	-640.69663	33.4
**2M4b**	-640.70601	0.21198	0	13.5	-640.72493	17.4
**TS4b**	-640.68282	0.20740	1 (-381.8)	25.2	-640.71231	22.4
**1M4b**	-640.69085	0.20942	0	21.4	-640.72333	16.8
**2TS4b**	-640.61951	0.20537	1 (-208.1)	63.7	-640.63514	69.6
**3P4b**	-531.17819	0.20128	0	-12.1	-531.19898	-6.1
**3TS4b**	-640.63796	0.20607	1 (-713.1)	52.5	-640.65585	57.0
**2P4b**	-584.15389	0.17284	0	-9.4	-584.17861	-11.8

**1TS5a**	-1097.64235	0.13926	1 (-513.5)	45.2	-1097.65254	50.0
**1M5a**	-1097.68192	0.14271	0	22.5	-1097.69341	26.5
**2TS5a**	-1097.67169	0.14055	1 (-253.9)	27.6	-1097.68601	29.8
**2M5a**	-988.19619	0.13131	0	-29.9	-988.22159	-31.4
**5TS5a**	-988.18174	0.12661	1 (-1278.9)	-23.8	-988.20461	-23.7
**1P5a**	-988.21108	0.13230	0	-38.7	-988.22960	-35.8
**4TS5a**	-988.13205	0.12584	1 (-1631.7)	6.9	-988.15284	8.3
**2P5a**	-988.22088	0.13294	0	-44.4	-988.23714	-40.2
**3TS5a**	-1097.61476	0.13756	1 (-1732.1)	61.4	-1097.64514	53.6
**3P5a**	-1041.13756	0.10354	0	-5.4	-1041.15071	-5.0
**1TS5b**	-1097.67399	0.14041	1 (-143.9)	26.0	-1097.69098	26.6
**2M5b**	-1097.69540	0.14255	0	14.0	-1097.70844	17.0
**TS5b**	-1097.68544	0.13843	1 (-339.5)	17.6	-1097.70963	13.6
**1M5b**	-1097.69239	0.14026	0	14.4	-1097.72149	7.4
**2TS5b**	-1097.62401	0.13724	1 (-315.1)	55.4	-1097.63359	60.6
**3P5b**	-988.17067	0.13195	0	-13.5	-988.18564	-8.5
**3TS5b**	-1097.62620	0.13685	1 (-1695.2)	53.8	-1097.63123	61.9
**2P5b**	-1041.13388	0.10332	0	-3.2	-1041.15212	-6.0
**1TS6a**	-1294.20337	0.27689	1 (-511.0)	47.5	-1294.21327	50.1
**1M6a**	-1294.24233	0.28087	0	25.6	-1294.25429	26.9
**2TS6a**	-1294.23100	0.27827	1 (-392.4)	31.0	-1294.24350	32.0
**2M6a**	-1184.75877	0.27086	0	-27.4	-1184.78312	-30.6
**5TS6a**	-1184.73158	0.26850	1 (-193.7)	-11.8	-1184.74726	-9.6
**3P6a**	-1184.74019	0.26927	0	-16.7	-1184.75093	-11.4
**3TS6a**	-1184.65675	0.26682	1 (-830.4)	34.1	-1184.67612	34.0
**1P6a**	-1184.77680	0.27033	0	-39.0	-1184.79176	-36.3
**4TS6a**	-1184.66986	0.26692	1 (-536.0)	25.9	-1184.68648	27.6
**2P6a**	-1184.75497	0.27007	0	-25.5	-1184.77028	-23.0
**TS6b**	-1294.25640	0.27820	1 (-249.7)	15.1	-1294.27380	13.0
**1M6b**	-1294.25897	0.28013	0	14.7	-1294.28149	9.4
**1TS6b**	-1294.24633	0.28014	1 (-127.1)	22.6	-1294.26244	21.3
**2M6b**	-1294.26158	0.28106	0	13.6	-1294.27388	14.7
**2TS6b**	-1294.17920	0.27460	1 (-353.3)	61.2	-1294.18901	63.9
**1P6b**	-1184.79099	0.27100	0	-47.5	-1184.80666	-45.3
**3P6b**	-1184.74019	0.26927	0	-16.7	-1184.75093	-11.4

Table S2. Partial atomic charges on selected atoms in **AZ1**-**AZ5**, **GV**, **GV2** and **1TSNa** and **1TSNb** (N=1-6) structures optimized at SMD(chloroform)//B3LYP/6-311+G(2d,p) level of theory.

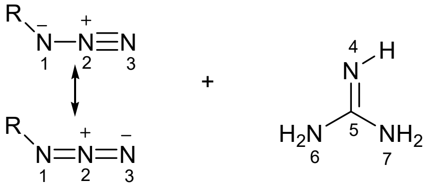

Structure	N1	N2	N3	N4	C5	N6	N7
			Hirshfeld charges				
**AZ1**	-0.20	0.17	-0.12	-	-	-	-
**AZ2**	-0.14	0.16	-0.14	-	-	-	-
**AZ3**	-0.12	0.18	-0.11	-	-	-	-
**AZ4**	-0.12	0.17	-0.14	-	-	-	-
**AZ5**	-0.11	0.18	-0.07	-	-	-	-
**GV**	-	-	-	-0.35	0.13	-0.18	-0.17
**GV2**	-	-	-	-0.24	0.13	-0.07	-0.06
**1TS1a**	-0.09	0.02	-0.14	-0.20	0.13	-0.21	-0.20
**1TS1b**	-0.20	-0.10	-0.19	-0.12	0.17	-0.16	-0.15
**1TS2a**	-0.03	0.04	-0.13	-0.21	0.12	-0.20	-0.22
**1TS2b**	-0.13	-0.09	-0.22	-0.11	0.17	-0.16	-0.14
**TS2b**	-0.21	-0.06	-0.16	-0.12	0.17	-0.14	-0.14
**1TS3a**	-0.02	0.04	-0.11	-0.20	0.12	-0.20	-0.22
**1TS3b**	-0.12	-0.06	-0.17	-0.11	0.18	-0.15	-0.14
**1TS4a**	-0.03	0.04	-0.13	-0.20	0.12	-0.20	-0.22
**1TS4b**	-0.14	-0.09	-0.21	-0.11	0.17	-0.16	-0.15
**TS4b**	-0.19	-0.05	-0.16	-0.12	0.17	-0.14	-0.14
**1TS5a**	-0.03	0.04	-0.10	-0.19	0.13	-0.20	-0.21
**1TS5b**	-0.12	-0.07	-0.14	-0.11	0.18	-0.15	-0.14
**TS5b**	-0.18	-0.02	-0.09	-0.12	0.18	-0.14	-0.13
**1TS6a**	-0.03	0.00	-0.13	-0.09	0.14	-0.06	-0.07
**TS6b**	-0.16	-0.02	-0.10	-0.07	0.16	-0.03	-0.03
**1TS6b**	-0.11	-0.09	-0.12	-0.04	0.18	-0.06	-0.04
			NBO charges				
**AZ1**	-0.56	0.25	-0.09	-	-	-	-
**AZ2**	-0.37	0.25	-0.12	-	-	-	-
**AZ3**	-0.34	0.26	-0.07	-	-	-	-
**AZ4**	-0.36	0.26	-0.11	-	-	-	-
**AZ5**	-0.33	0.26	-0.02	-	-	-	-
**GV**	-	-	-	-0.83	0.60	-0.85	-0.85
**GV2**	-	-	-	-0.62	0.65	-0.54	-0.55
**1TS1a**	-0.37	0.08	-0.18	-0.67	0.55	-0.87	-0.86
**1TS1b**	-0.62	-0.03	-0.23	-0.55	0.66	-0.81	-0.81
**1TS2a**	-0.20	0.11	-0.19	-0.66	0.53	-0.86	-0.90
**1TS2b**	-0.42	-0.05	-0.27	-0.53	0.67	-0.81	-0.81
**TS2b**	-0.49	-0.01	-0.21	-0.58	0.66	-0.81	-0.81
**1TS3a**	-0.20	0.11	-0.17	-0.63	0.54	-0.86	-0.89
**1TS3b**	-0.42	-0.02	-0.19	-0.54	0.67	-0.81	-0.80

**1TS4a**	-0.20	0.11	-0.19	-0.65	0.54	-0.86	-0.90
**1TS4b**	-0.43	-0.04	-0.24	-0.53	0.67	-0.81	-0.81
**TS4b**	-0.48	0.00	-0.20	-0.58	0.66	-0.81	-0.81
**1TS5a**	-0.22	0.11	-0.15	-0.62	0.54	-0.86	-0.89
**1TS5b**	-0.43	-0.01	-0.14	-0.55	0.67	-0.80	-0.80
**TS5b**	-0.47	0.03	-0.11	-0.59	0.66	-0.80	-0.80
**1TS6a**	-0.23	0.06	-0.19	-0.45	0.65	-0.54	-0.58
**TS6b**	-0.46	0.02	-0.09	-0.45	0.72	-0.49	-0.50
**1TS6b**	-0.43	-0.05	-0.11	-0.41	0.77	-0.53	-0.52
Mülliken charges							
**AZ1**	-0.70	0.56	-0.15	-	-	-	-
**AZ2**	-0.51	0.63	-0.30	-	-	-	-
**AZ3**	-0.25	0.85	-0.74	-	-	-	-
**AZ4**	-0.19	0.31	-0.21	-	-	-	-
**AZ5**	0.06	0.43	-0.65	-	-	-	-
**GV**	-	-	-	-0.62	0.38	-0.50	-0.51
**GV2**	-	-	-	-0.49	0.37	-0.19	-0.13
**1TS1a**	-0.17	0.14	-0.29	-0.45	0.31	-0.58	-0.55
**1TS1b**	-0.55	0.03	-0.30	-0.23	0.35	-0.43	-0.47
**1TS2a**	0.07	0.03	-0.25	-0.53	0.36	-0.56	-0.61
**1TS2b**	-0.23	-0.07	-0.32	-0.24	0.33	-0.48	-0.49
**TS2b**	-0.53	0.00	-0.17	-0.35	0.46	-0.50	-0.49
**1TS3a**	0.15	-0.30	0.12	-0.58	0.46	-0.58	-0.63
**1TS3b**	-0.13	-0.16	-0.13	-0.25	0.31	-0.46	-0.47
**1TS4a**	0.26	-0.08	-0.22	-0.59	0.44	-0.61	-0.64
**1TS4b**	-0.05	-0.17	-0.26	-0.25	0.35	-0.50	-0.48
**TS4b**	-0.35	-0.22	0.00	-0.35	0.47	-0.50	-0.49
**1TS5a**	0.27	-0.42	0.16	-0.63	0.50	-0.58	-0.63
**1TS5b**	-0.17	-0.19	-0.07	-0.27	0.36	-0.46	-0.48
**TS5b**	-0.33	-0.32	0.11	-0.41	0.47	-0.49	-0.47
**1TS6a**	0.20	-0.55	0.18	-0.31	-0.01	-0.20	-0.12
**TS6b**	-0.23	-0.23	-0.05	0.02	-0.24	0.07	0.06
**1TS6b**	-0.02	-0.03	-0.02	0.05	-1.24	0.09	0.17
**AZ1**	-0.70	0.56	-0.15				

Only the NBO partial charges are consistent with reaction energetics what can be explained by three points:

1. Both N1 and N3 reaction centers in azides are partially negative. However the negative partial charge on N1 azide atom is much larger than on N3 atom. Therefore the approach of partially positive C4 atom from guanidine to N1 is more preferable than to N3 making the (b) reaction direction preferable with respect to (a) azide guanidine interaction. This is valid for all azides investigated herein.

2. Partial charge on N1 is the biggest in unsubstituted AZ1 (-0.56|e|). All substituents decrease it to the range between -033 |e| and -0.37|e|. The approach of guanidine with its imino N4 partially negative atom toward N1 ((a) direction) has unfavorable interaction which is decreased upon substitution. Consequently, the relative energies of **1TSNa** N=2-5 structures are smaller with respect to **1TS1a**.

3. We noted that the total partial charge in the N_3 group (sum of partial charges on N1, N2 and N3 atoms) is substantially increased (the difference between **AZN** N=1-5 and **1TSNb** N=1-5 is in the range 0.47 and 0.50 |e|) while guanidine approaches azides in (b) direction. Electron-withdrawing substituents on azides should favorably affect the energetics of the reaction with increasing negative charges on the reaction center. Indeed, in the case of the strongest withdrawal C_6F_5 group relative energy of **1TS5b** is the lowest. In contrast to the changes along path (b), an increase in negative charges within the azide subunit along path (a) is significantly smaller being localized at N2 and N3. Actually, a loss of some electron density occurs at the nitrogen atom N1, as described in Point 2, is presumably triggered by the approach of the electron richer side of the dipolarophile (imino nitrogen atom). Described changes in the electron density, primarily at the N1 atom, are the most likely reason for the weaker effect of substituents.

3. Explicit solvation with one $CHCl_3$ molecule

Table S3. Energies, zero point vibrational energies (ZPVE), relative energies for stationary points along reaction path for cycloaddition of **AZ3** and **GV** in the direction (a) with one molecule of solvent explicitly included in the calculations. The B3LYP/6-311+G(2d,p) SMD(solvent=chloroform) theoretical model was used with Integral=ultrafine option.

Structure	Energy/a.u.	ZPVE/a.u.	N_{imag} (cm^{-1})	E_{rel}/kcal mol^{-1}	ΔE_{rel}/kcal mol^{-1}
**N_2**	-109.55829	0.00555	0	-	
**NH_3**	-56.58795	0.03404	0	-	
**AZ3**	-395.95997	0.10300	0	-	
**GV · $HCCl_3$**	-1624.86732	0.09547	0	-	
**1TS3a · $HCCl_3$**	-2020.74432	0.19958	1(-513.7)	52.8	1.1
**1M3a · $HCCl_3$**	-2020.78941	0.20325	0	26.8	1.5
**2TS3a · $HCCl_3$**	-2020.77653	0.20148	1(-314.2)	33.7	2.6
**2M3a · $HCCl_3$**	-1911.29901	0.19157	0	-19.7	2.0
**5TS3a · $HCCl_3$**	-1911.28422	0.18729	1(-1417.1)	-13.1	3.1
**1P3a · $HCCl_3$**	-1911.31655	0.19300	0	-29.8	2.4
**4TS3a · $HCCl_3$**	-1911.23543	0.18619	1(-1748.3)	16.8	2.6
**2P3a · $HCCl_3$**	-1911.32447	0.19289	0	-34.8	2.0
**3TS3a · $HCCl_3$**	-2020.72844	0.19798	1(-1823.1)	61.7	1.9
**3P3a · $HCCl_3$**	-1964.25008	0.16434	0	-6.8	2.5

ΔE_{rel} with respect to SMD(solvent=Chloroform)//B3LYP/6-311+G(2d,p)

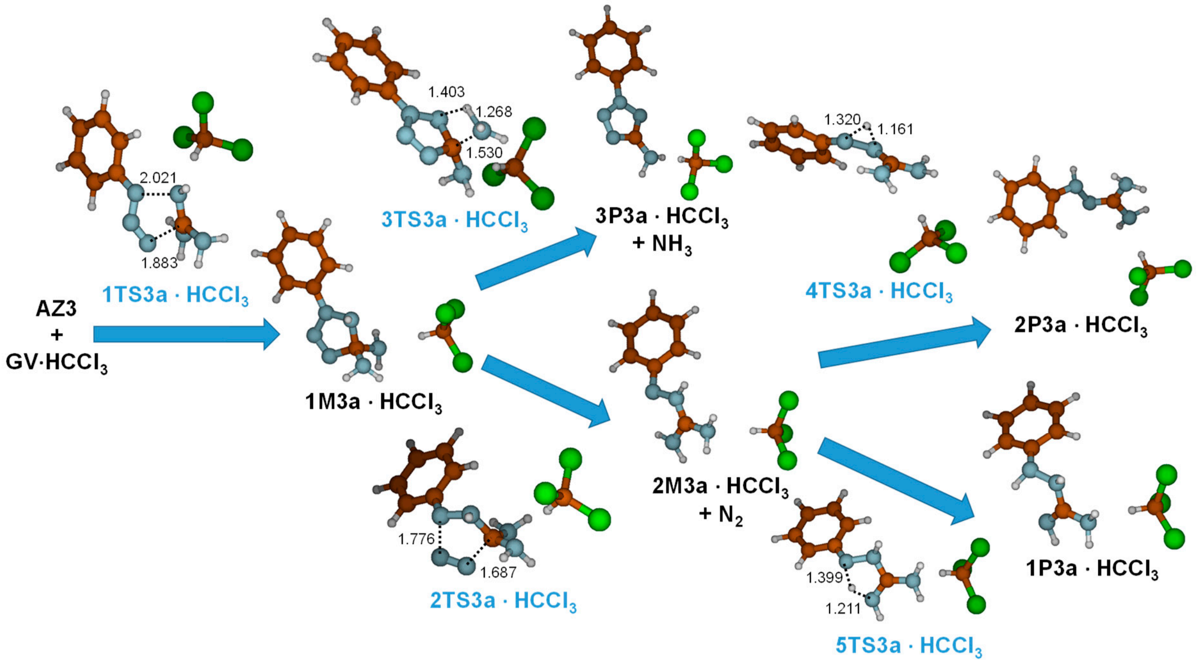

Figure S7. Stationary points along reaction path for reaction of **AZ3** with **GV** in the direction (a) with one molecule of solvent explicitly included in the calculation. The B3LYP/6-311+G(2d,p) SMD(solvent=chloroform) theoretical model was used with Integral=ultrafine option.

2. Coordinates of all structures associated with this article

Table S4. Coordinates of all structures (Å) optimized at B3LYP/6-311+G(2d,p) level of theory in the gas phase using Integral=ultrafine option.

AZ1 (az1.log)

N	0.000000	0.112484	0.000000	$H-N_3$
N	-0.255738	1.209642	0.000000	
N	0.101109	-1.122304	0.000000	
H	1.082403	-1.398749	0.000000	

AZ2 (az2.log)

N	0.000000	0.724834	0.000000	$Me-N_3$
N	-0.514926	1.732752	0.000000	
N	0.678339	-0.299513	0.000000	
C	-0.070416	-1.568906	0.000000	
H	0.669348	-2.364840	0.000000	
H	-0.695373	-1.664116	0.892327	
H	-0.695373	-1.664116	-0.892327	

AZ3 (az3.log)

N	2.201958	0.990686	0.000000	$Ph-N_3$
N	3.310874	0.773078	0.000000	
N	1.030240	1.369863	0.000000	
C	0.000000	0.391721	0.000000	
C	-1.307940	0.877272	-0.000000	
C	-2.372618	-0.012788	-0.000000	
C	-2.144959	-1.386517	-0.000000	
C	-0.838714	-1.864658	-0.000000	
C	0.236593	-0.984186	0.000000	
H	-1.471009	1.947424	-0.000000	
H	-3.386175	0.369840	-0.000000	
H	-2.978057	-2.078229	-0.000000	
H	-0.650638	-2.931693	-0.000000	
H	1.250206	-1.367800	0.000000	

AZ4 (az4.log)

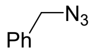

N	1.757466	-2.624131	0.000000
N	2.530890	-3.450209	0.000000
N	0.828678	-1.821575	0.000000
C	1.216927	-0.395819	0.000000
C	0.000000	0.500555	0.000000
H	1.830699	-0.184476	0.883415
H	1.830699	-0.184476	-0.883415
C	0.195617	1.883731	0.000000
C	-0.887451	2.752604	0.000000
C	-2.185798	2.248922	0.000000
C	-2.386391	0.874421	0.000000
C	-1.299670	0.002554	0.000000
H	1.204126	2.285272	0.000000
H	-0.718589	3.822941	0.000000
H	-3.032557	2.924757	0.000000
H	-3.392921	0.472890	0.000000
H	-1.460097	-1.067313	0.000000

AZ5 (az7.log)

N	-1.241706	2.877311	0.000000

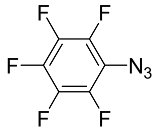

N	-2.150592	3.541269	0.000000
N	-0.127794	2.335256	0.000000
C	0.000000	0.942935	0.000000
C	1.304872	0.436128	0.000000
C	1.552696	-0.926798	0.000000
C	0.496668	-1.828184	0.000000
C	-0.806072	-1.351776	0.000000
C	-1.043846	0.013580	0.000000
F	2.338685	1.278019	0.000000
F	2.808260	-1.375862	0.000000
F	0.732523	-3.139982	0.000000
F	-1.829843	-2.206201	0.000000
F	-2.314654	0.444899	0.000000

GU (gv.log)

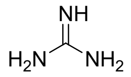

C	-0.016526	0.121706	0.000146
N	-0.212399	1.383243	0.009945
N	1.276146	-0.386454	-0.082988
N	-0.980424	-0.887126	0.078311
H	-1.201300	1.615219	-0.046940
H	-1.929445	-0.565338	-0.042018
H	-0.780825	-1.707381	-0.478872
H	1.458653	-1.182962	0.512439
H	1.968813	0.342586	0.017644

GU2 (gv2.log)

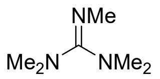

C	-0.026792	0.323984	0.018009
N	0.260345	1.562595	-0.127960
N	-1.368585	-0.064403	0.117627
N	0.891770	-0.725489	0.139791
C	1.599373	2.051107	0.126868
C	2.018079	-0.841259	-0.764918
C	0.681531	-1.851064	1.028592
C	-1.844048	-1.114259	-0.776926
C	-2.343277	0.999516	0.313159
H	1.527458	3.026909	0.614629
H	2.197192	1.390279	0.767660
H	2.155403	2.210290	-0.804602
H	1.968721	-1.791704	-1.313575
H	2.000055	-0.033175	-1.491823
H	2.978581	-0.810654	-0.236745
H	0.482963	-2.785170	0.483653
H	1.574309	-2.013185	1.643852
H	-0.160712	-1.650368	1.686384
H	-2.717704	-1.605721	-0.342221
H	-2.133110	-0.707413	-1.757100
H	-1.072549	-1.863722	-0.935416
H	-3.284325	0.551694	0.641859
H	-1.985429	1.690630	1.072340
H	-2.524751	1.574230	-0.603802

1TS1a (gv_az1a_ts1.log)

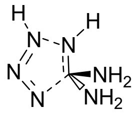

N	1.618919	-0.713225	-0.000302
N	0.590751	-1.284231	-0.059740
N	1.870649	0.550376	-0.091072
H	2.384919	0.873839	0.731643
C	-0.752492	0.194811	0.013535
N	0.074022	1.262230	0.043452

N	-1.399698	-0.200940	1.216128
N	-1.573342	-0.062494	-1.114287
H	0.168886	1.683496	-0.876769
H	-1.094566	0.122751	-1.987191
H	-1.886618	-1.027788	-1.114730
H	-0.951729	0.234796	2.013434
H	-2.375043	0.082031	1.193145

2TS1a (gv_az1a_ts3.log)

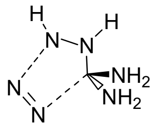

N	1.570278	-0.887176	-0.370114
N	0.448187	-1.187245	-0.508298
N	1.585243	0.839233	0.203114
H	1.699801	0.617617	1.194032
C	-0.630574	0.061672	0.018246
N	0.276066	1.161358	-0.023940
N	-1.032147	-0.458271	1.282715
N	-1.752679	0.249529	-0.806533
H	0.209789	1.647382	-0.913963
H	-1.521032	0.346453	-1.787548
H	-2.420644	-0.499584	-0.680791
H	-0.245733	-0.723652	1.862542
H	-1.603364	0.219752	1.777634

3TS1a (gv_az1a_ts6.log)

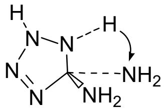

N	1.564551	-0.470135	-0.601206
N	0.361929	-0.761070	-0.933604
N	1.571446	0.328390	0.453206
H	2.384856	0.291012	1.052045
C	-0.525709	-0.130925	0.030892
N	0.304728	0.524262	1.021318
N	-1.615158	-0.921524	0.524922
N	-1.142268	1.197539	-0.571182
H	-0.410369	1.578804	0.358951
H	-0.791697	1.391231	-1.507978
H	-2.159271	1.264545	-0.536268
H	-1.880246	-1.618087	-0.166971
H	-1.305618	-1.404190	1.360696

4TS1a (gv_az1a_ts4.log)

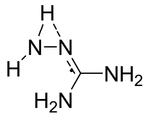

N	1.908790	0.004458	0.078721
H	2.538201	-0.534717	-0.520075
C	-0.453317	-0.014404	-0.017285
N	0.600875	-0.795104	-0.103452
N	-0.305663	1.323354	0.052094
N	-1.720844	-0.550123	-0.061181
H	1.258191	-1.055758	0.754903
H	-1.010158	1.913239	-0.363602
H	0.688125	1.566166	-0.043849
H	-1.731319	-1.560109	-0.032751
H	-2.405240	-0.120492	0.545813

5TS1a (gv_az1a_ts5.log)

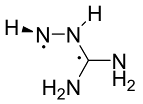

N	-1.978475	-0.156549	-0.050257
C	0.428556	0.004845	-0.007582
N	-0.690233	-0.673036	-0.035108
N	0.384111	1.360439	-0.100293
N	1.668107	-0.599109	0.118493

H	-0.606184	-1.671623	-0.222204
H	1.644971	-1.610891	0.130423
H	2.365312	-0.236496	-0.521634
H	-0.576512	1.685009	-0.196424
H	0.979574	1.900347	0.508051
H	-2.063072	0.382367	0.817434

6TS1a (gv_az1a_ts5a.log)

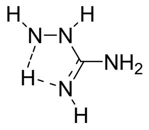

N	-1.841526	-0.098824	-0.129340
C	0.462146	0.035401	0.020628
N	-0.599357	-0.771758	0.046002
N	0.133432	1.315334	-0.042077
N	1.752143	-0.437270	0.074775
H	-0.497188	-1.727848	-0.273229
H	1.852569	-1.441983	0.030270
H	2.419127	0.053164	-0.503835
H	-1.008821	1.193860	-0.013168
H	0.756597	2.024013	0.312797
H	-2.408007	-0.365988	0.677872

1M1a (gv_az1a_m1.log)

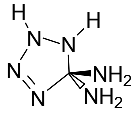

N	-1.508728	-0.680785	0.497783
N	-0.318772	-0.973928	0.682990
N	-1.660328	0.469333	-0.255192
H	-2.360276	0.302908	-0.973291
C	0.586534	-0.006869	-0.024016
N	-0.379402	0.812522	-0.806268
N	1.540054	-0.757680	-0.789019
N	1.332518	0.833619	0.886241
H	-0.211105	1.791461	-0.591427
H	0.726987	1.206325	1.610831
H	2.057420	0.282170	1.334666
H	1.068497	-1.339907	-1.471733
H	2.161883	-0.123308	-1.280695

2M1a (gv_az1a_m3.log)

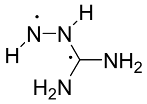

N	2.022587	-0.190254	-0.025326
H	1.990281	0.791002	-0.310687
C	-0.418319	0.005599	0.016828
N	0.735250	-0.614338	0.051614
N	-0.504567	1.381620	0.082756
N	-1.634407	-0.675279	-0.100546
H	0.682644	-1.615428	0.204385
H	-1.550794	-1.652344	-0.344490
H	-2.261742	-0.538185	0.686636
H	0.374155	1.851677	0.248783
H	-1.056671	1.817441	-0.645080

3M1a (gv_az1a_m4a.log)

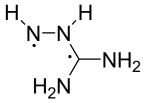

N	1.902207	-0.012526	0.070138
C	-0.434340	0.006207	-0.016855
N	0.690760	-0.662168	-0.002729
N	-0.359388	1.365638	0.057138
N	-1.692738	-0.588799	-0.067025
H	0.626940	-1.672501	0.093356
H	-1.674886	-1.585127	-0.239171
H	-2.280669	-0.371124	0.731674

H	0.613879	1.660798	-0.051843
H	-1.044968	1.866319	-0.491583
H	2.579861	-0.650620	-0.343956

1P1a (gv_az1a_m5.log)

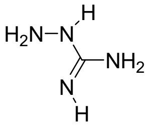

N	-1.914776	-0.111497	-0.068504
C	0.484068	0.116199	0.016818
N	-0.636320	-0.711903	0.057200
N	0.345654	1.386475	-0.011892
N	1.683975	-0.585665	0.082004
H	-0.568180	-1.530053	-0.533024
H	1.640808	-1.590692	-0.001935
H	2.464576	-0.168094	-0.399933
H	-1.780509	0.797136	-0.513323
H	1.237481	1.867730	0.064697
H	-2.248310	0.084912	0.870955

2P1a (gv_az1a_m4.log)

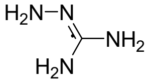

N	1.842512	-0.032004	-0.037206
H	2.447187	-0.547770	-0.671111
C	-0.425617	-0.037537	0.004567
N	0.610554	-0.800872	0.026414
N	-0.415755	1.340591	0.068839
N	-1.687226	-0.615805	-0.087037
H	2.284898	-0.126279	0.875831
H	-1.141195	1.793337	-0.469237
H	0.517727	1.700180	-0.104326
H	-1.635674	-1.622601	0.001065
H	-2.369838	-0.215008	0.543313

3P1a (gv_az1a_m6.log)

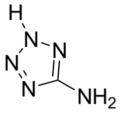

N	-1.353869	0.758420	0.007108
N	-0.096814	1.126211	-0.000997
N	-1.346695	-0.555328	0.005473
H	-2.195598	-1.100828	0.011597
C	0.632173	-0.022146	-0.008953
N	-0.130326	-1.110101	-0.002029
N	2.002675	-0.034395	-0.077356
H	2.437998	0.815789	0.248956
H	2.439765	-0.875735	0.267783

1TS1b (gv_az1b_ts1.log)

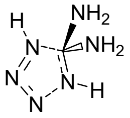

N	-1.784564	0.396458	-0.113762
N	-1.583944	-0.812596	0.174922
N	-0.788635	1.181549	-0.358065
H	-1.129458	2.069750	-0.722414
N	-0.108660	-1.164097	-0.005196
C	0.794335	-0.159917	0.048288
H	-0.034707	-1.737628	-0.837679
N	1.161746	0.290315	1.301691
N	1.762783	-0.070019	-0.931416
H	1.317005	1.289511	1.347410
H	0.506994	-0.007825	2.013799
H	1.411423	0.077569	-1.865752
H	2.551647	0.516869	-0.702306

2TS1b (gv_az1b_ts3.log)

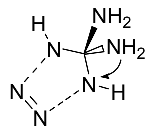

N	-2.078428	0.287065	0.277777
N	-1.895916	-0.577296	-0.408960
N	-0.037353	0.690395	1.197451
H	-0.002870	1.707152	1.177472
N	-0.004472	-1.141649	-0.237133
C	0.557304	0.176380	0.077294
H	0.253168	-1.334481	-1.213542
N	1.870378	-0.567005	0.397330
N	0.839854	0.974394	-1.109383
H	1.789709	-1.000218	1.313482
H	2.183692	-1.235065	-0.299393
H	0.006827	1.493724	-1.370634
H	1.567209	1.649279	-0.890728

3TS1b (gv_az1b_ts6.log)

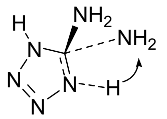

N	1.734291	-0.364454	0.381966
N	1.587390	0.447041	-0.568156
N	0.448668	-0.915146	0.670366
H	0.329378	-1.231720	1.621072
N	0.297046	0.589084	-0.973754
C	-0.512703	-0.164149	-0.015981
H	-0.408683	1.596939	-0.286814
N	-1.069118	1.178282	0.688466
N	-1.669340	-0.902807	-0.432225
H	-2.078010	1.259922	0.815020
H	-0.592784	1.369458	1.566748
H	-2.118721	-0.427992	-1.211311
H	-1.357520	-1.805714	-0.775468

1M1b (gv_az1b_m1.log)

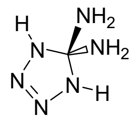

N	1.678859	0.523843	-0.356127
N	1.681763	-0.496788	0.362423
N	0.385980	0.769694	-0.809353
H	0.213909	1.761079	-0.921086
N	0.400436	-1.019359	0.449252
C	-0.575506	-0.015789	-0.000509
H	0.220476	-1.425719	1.358888
N	-1.624141	-0.673047	-0.746715
N	-1.196095	0.706783	1.085867
H	-2.168046	0.002476	-1.275444
H	-1.203292	-1.320238	-1.404019
H	-0.708256	1.545028	1.369514
H	-2.189369	0.854228	0.977770

1P1b = **1P1a**(gv_az1b_m3.log)

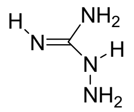

N	-0.345547	1.386402	-0.011851
H	-1.237283	1.867857	0.064190
N	0.636288	-0.711756	0.057573
C	-0.484007	0.116176	0.016795
H	0.568408	-1.531032	-0.531024
N	1.914719	-0.111610	-0.068730
N	-1.684074	-0.585728	0.082218
H	1.780247	0.796776	-0.514116
H	2.248152	0.085926	0.870606
H	-2.464431	-0.167252	-0.399447
H	-1.640743	-1.590485	-0.005448

2P1b (gv_az1b_m6.log)

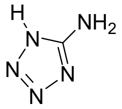

N	1.488297	0.575159	0.004605
N	1.413200	-0.702038	0.009528
N	0.198662	1.025593	-0.006507
H	0.000680	2.011538	-0.067464
N	0.118392	-1.131806	0.010782
C	-0.621135	-0.042661	-0.003394
N	-1.992764	0.008497	-0.081667
H	-2.413328	-0.901901	0.050077
H	-2.441041	0.718490	0.480560

1TS2a (gv_az2a_ts1.log)

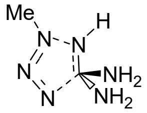

N	-0.971635	1.109213	0.280141
N	0.149306	1.336263	0.566816
N	-1.496367	0.217188	-0.464157
C	-2.622360	-0.539730	0.077282
C	1.085249	-0.292400	-0.091001
N	0.098121	-0.928058	-0.737702
N	1.530431	-0.911384	1.103679
N	2.093098	0.395230	-0.853978
H	0.140334	-0.668420	-1.719608
H	-2.766936	-1.386183	-0.589801
H	-2.432125	-0.901988	1.091179
H	-3.525237	0.073863	0.065682
H	1.896646	-0.250550	1.779135
H	0.807837	-1.484828	1.521819
H	2.357734	1.269172	-0.412521
H	2.923742	-0.187434	-0.917158

2TS2a (gv_az2a_ts3.log)

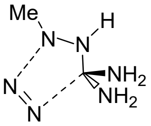

N	0.857986	1.430416	-0.475330
N	-0.268432	1.271673	-0.730062
N	1.342297	0.150352	0.705023
C	2.221363	-0.691199	-0.098746
C	-0.926318	-0.204092	0.058355
N	0.112263	-0.378112	1.004504
N	-1.033594	-1.185038	-0.953013
N	-2.170420	0.107443	0.632830
H	-0.205865	0.003099	1.889414
H	2.562927	-1.537445	0.502169
H	1.790582	-1.073613	-1.034635
H	3.088152	-0.084136	-0.360879
H	-1.447825	-0.810956	-1.800454
H	-0.150066	-1.627792	-1.164568
H	-2.343988	1.099520	0.726253
H	-2.944890	-0.374067	0.197379

3TS2a (gv_az2a_ts6.log)

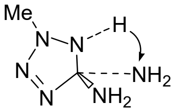

N	-0.899526	1.040093	-0.630569
N	0.382572	1.198079	-0.588774
N	-1.249297	-0.072678	-0.037153
C	-2.616326	-0.358609	0.322435
C	0.931661	0.058408	0.114301
N	-0.185315	-0.764451	0.545121
N	1.952371	0.317312	1.091065
N	1.519486	-1.001687	-0.907458
H	0.525379	-1.563020	-0.388845
H	1.398143	-0.698071	-1.872269

H	2.479726	-1.296447	-0.731001
H	2.440844	1.176542	0.850725
H	1.503320	0.453181	1.989699
H	-2.854273	-1.395962	0.081939
H	-2.770422	-0.200446	1.393587
H	-3.256767	0.308744	-0.249869

4TS2a (gv_az2a_ts4.log)

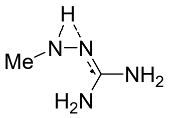

N	-1.331054	0.330917	-0.411386
C	-2.491812	-0.192271	0.290734
C	0.974830	-0.030714	0.003190
N	-0.179061	-0.659479	-0.121499
N	1.017827	1.315460	0.013093
N	2.137447	-0.744025	0.174232
H	-0.704826	-0.743449	-1.094890
H	-2.675964	-1.262235	0.109931
H	-2.428756	-0.054092	1.381283
H	-3.361054	0.362888	-0.072529
H	1.723024	1.777594	0.567175
H	0.056728	1.683072	-0.024287
H	2.017559	-1.742676	0.079161
H	2.959065	-0.383305	-0.290473

5TS2a (gv_az2a_ts5a.log)

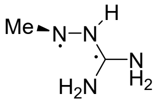

N	1.455066	-0.460137	-0.580928
C	2.071796	0.187440	0.564182
C	-0.850952	0.003637	-0.062631
N	0.147010	-0.812643	-0.302200
N	-0.697116	1.330663	-0.315720
N	-2.089575	-0.490397	0.305941
H	-0.111273	-1.790170	-0.445332
H	1.717170	1.211094	0.819730
H	1.985888	-0.402553	1.495424
H	3.135316	0.282084	0.335782
H	-1.013794	1.997510	0.371981
H	0.219291	1.521418	-0.712728
H	-2.117499	-1.112574	1.098452
H	-2.847851	0.174323	0.277738

6TS2a (gv_az2a_ts5.log)

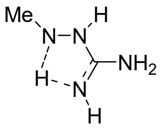

N	1.281498	0.200517	-0.503131
C	2.395301	-0.168258	0.353650
C	-0.965586	0.010957	-0.003912
N	0.176425	-0.639359	-0.218513
N	-0.813816	1.328942	-0.004305
N	-2.167175	-0.653081	0.084793
H	0.151492	-1.589021	-0.575667
H	2.184953	-0.089040	1.436268
H	2.762670	-1.193123	0.171685
H	3.220557	0.509437	0.122869
H	-1.457587	1.927928	0.489345
H	0.315569	1.369266	-0.153294
H	-2.137595	-1.558160	0.533392
H	-2.956873	-0.092613	0.365060

1M2a (gv_az2a_m1.log)

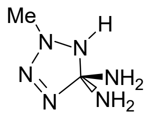

N	0.870043	1.132645	-0.456375
N	-0.359039	1.102357	-0.654267
N	1.298011	0.137023	0.368906
C	2.584130	-0.447207	0.007469
C	-0.965661	-0.100181	0.011298
N	0.219777	-0.797078	0.555972
N	-1.646937	-0.966716	-0.918898
N	-1.885641	0.323262	1.043393
H	0.073499	-0.888076	1.557265
H	2.956228	-1.032471	0.846900
H	2.500472	-1.091898	-0.873630
H	3.273247	0.371334	-0.192893
H	-2.271146	-0.425649	-1.511202
H	-0.972609	-1.442183	-1.507719
H	-1.933777	1.333662	1.105662
H	-2.810227	-0.060840	0.891914

2M2a (gv_az2a_m3.log)

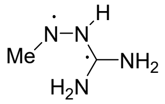

N	1.492787	-0.812979	-0.260454
C	2.075341	0.367319	0.324796
C	-0.819527	0.019582	-0.021003
N	0.154424	-0.854015	-0.200552
N	-0.709217	1.382351	-0.290611
N	-2.098520	-0.513690	0.209802
H	-0.203783	-1.797585	-0.324630
H	2.032020	1.292930	-0.285264
H	1.642348	0.613290	1.310612
H	3.136434	0.157147	0.466283
H	-1.070142	1.985894	0.440803
H	0.211636	1.679396	-0.581715
H	-2.327277	-0.687849	1.181354
H	-2.832434	0.023705	-0.237490

3M2a (gv_az2a_m5a.log)

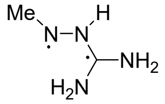

N	1.310250	0.461200	-0.121386
C	2.565879	-0.228156	0.077956
C	-0.970592	-0.026684	0.021000
N	0.286421	-0.395990	-0.049113
N	-1.237966	1.324866	-0.046619
N	-1.966817	-1.016043	-0.001260
H	0.433020	-1.406129	-0.108351
H	2.740246	-0.541675	1.122499
H	2.673372	-1.127636	-0.556587
H	3.372214	0.453626	-0.193811
H	-2.031661	1.642020	0.492921
H	-0.389689	1.874275	0.079810
H	-2.489380	-1.087353	0.866685
H	-2.623068	-0.896309	-0.768257

1P2a (gv_az2a_m5.log)

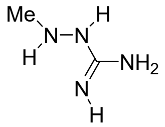

N	1.372917	-0.182674	-0.614794
C	2.254969	0.046290	0.532310
C	-0.942776	0.116427	-0.021301
N	0.137450	-0.745374	-0.203314
N	-0.782988	1.381787	-0.095257
N	-2.134405	-0.581852	0.187086
H	-0.092512	-1.562387	-0.754053

H	1.799440	0.669405	1.310688
H	2.542330	-0.912653	0.965280
H	3.157030	0.540887	0.166221
H	-1.632466	1.891133	0.131461
H	1.150697	0.717408	-1.037564
H	-2.041406	-1.417689	0.749477
H	-2.907080	-0.005612	0.486383

2P2a (gv_az2a_m4.log)

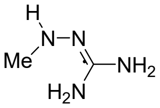

N	-1.287583	0.339364	-0.332496
C	-2.448857	-0.239226	0.346047
C	0.930974	-0.048599	-0.031956
N	-0.196915	-0.612732	-0.292485
N	1.152302	1.310772	0.050865
N	2.050449	-0.843996	0.186475
H	-1.524117	0.436847	-1.317485
H	-2.677003	-1.254937	0.000070
H	-2.258421	-0.277421	1.419426
H	-3.311784	0.407503	0.171638
H	1.840018	1.585986	0.738186
H	0.270357	1.810219	0.116350
H	1.854974	-1.816018	-0.015787
H	2.885515	-0.519089	-0.283457

3P2a (gv_az2a_m6.log)

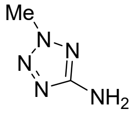

N	-0.621079	1.198997	0.006154
N	0.692618	1.204948	0.000809
N	-0.991806	-0.062317	0.001304
C	-2.378896	-0.478552	0.004940
C	1.067956	-0.096976	-0.009444
N	0.029392	-0.927352	-0.006472
N	2.380604	-0.498749	-0.076821
H	3.035513	0.194785	0.253892
H	2.558301	-1.425623	0.280555
H	-2.588935	-1.065497	-0.888845
H	-2.579629	-1.080823	0.890697
H	-2.987716	0.421635	0.015908

TS2b (gv_az2b_ts2.log)

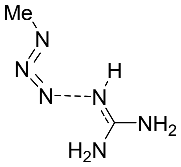

N	-1.601803	0.731134	-0.089419
N	-0.427431	1.082771	-0.115842
N	-2.084469	-0.436131	0.058799
C	-3.544685	-0.456750	0.026847
N	0.569006	-0.330160	0.075272
C	1.842153	-0.170422	0.029547
H	0.109944	-1.230181	0.069497
N	2.368646	1.083713	0.151423
N	2.749279	-1.193569	-0.145523
H	-3.889578	-1.102909	-0.787536
H	-3.929568	-0.877472	0.961830
H	-3.971604	0.541120	-0.111697
H	3.215252	1.270586	-0.364024
H	1.663232	1.810544	0.104086
H	3.632229	-1.085169	0.334070
H	2.372687	-2.127786	-0.087556

1TS2b (gv_az2b_ts1.log)

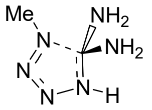

N	1.332892	0.938176	0.046908
N	0.400323	1.802722	-0.053292
N	1.070657	-0.312666	0.033376
C	2.224585	-1.191993	-0.091430
N	-0.911129	1.121116	-0.395591
C	-1.100897	-0.148795	0.002019
H	-1.109569	1.294365	-1.374082
N	-1.321673	-0.375481	1.346812
N	-1.613496	-1.111593	-0.831213
H	2.081699	-2.078197	0.532361
H	2.354986	-1.531976	-1.125832
H	3.139767	-0.681422	0.219966
H	-0.915130	-1.241469	1.676770
H	-1.017746	0.407839	1.911822
H	-2.438198	-1.594241	-0.508835
H	-1.540957	-0.966094	-1.824697

2TS2b (gv_az2b_ts3.log)

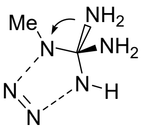

N	1.901695	0.843870	-0.025498
N	2.281604	-0.200645	-0.091848
N	-0.168003	0.786108	-0.515147
C	-0.853071	1.875770	0.127113
N	0.459214	-1.467827	-0.594532
C	-0.392786	-0.571121	0.026137
H	0.669198	-2.232351	0.045011
N	-1.750160	-0.653812	-0.667806
N	-0.627134	-0.623864	1.458800
H	-1.652395	2.264378	-0.520609
H	-1.228796	1.654920	1.128450
H	-0.117173	2.684534	0.180687
H	-2.272541	0.215002	-0.711907
H	-1.595880	-1.026298	-1.601035
H	-1.256140	-1.392348	1.665693
H	0.248364	-0.782545	1.946425

3TS2b (gv_az2b_ts6.log)

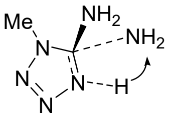

N	1.454296	0.903098	-0.034804
N	0.572858	1.796436	-0.144852
N	0.817299	-0.351395	-0.203624
C	1.503364	-1.531015	0.274468
N	-0.677421	1.288711	-0.348140
C	-0.553162	-0.145841	-0.199303
H	-1.389468	1.074826	0.804929
N	-1.335661	-0.101974	1.288049
N	-1.314397	-1.073060	-0.980052
H	-2.109774	-0.740729	1.469561
H	-0.672152	-0.154231	2.057155
H	-2.275340	-0.752249	-1.060159
H	-0.928598	-1.083625	-1.920042
H	0.973302	-2.415159	-0.077679
H	2.514361	-1.538163	-0.129974
H	1.567639	-1.562253	1.369183

1M2b (gv_az2b_m2.log)

N	1.553526	-0.827014	-0.055964
N	0.262282	-1.058330	-0.082979
N	1.958853	0.379607	0.032081

C	3.408963	0.487455	0.043610
N	-0.454949	0.167120	-0.019841
C	-1.750917	0.168972	0.011052
H	0.176665	0.980972	0.025901
N	-2.415298	-1.007718	0.137927
N	-2.492483	1.323022	-0.101706
H	3.745318	1.099365	-0.800381
H	3.738604	0.988317	0.960214
H	3.885387	-0.495623	-0.017052
H	-3.309746	-1.083793	-0.321529
H	-1.800648	-1.813337	0.068154
H	-3.307444	1.367576	0.495726
H	-1.959926	2.181156	-0.105637

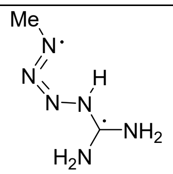

2M2b (gv_az2b_m1.log)

N	1.636519	-0.390706	-0.052056
N	1.152649	-1.538253	0.068012
N	0.636311	0.519423	-0.331128
C	0.939251	1.905237	-0.028598
N	-0.218516	-1.477237	-0.146789
C	-0.672657	-0.086739	-0.013357
H	-0.721849	-2.186492	0.369076
N	-1.742499	0.154896	-0.946988
N	-1.156278	0.354123	1.281467
H	0.171532	2.540766	-0.470588
H	0.990202	2.112507	1.044448
H	1.896625	2.148754	-0.486791
H	-2.031240	1.127038	-0.921939
H	-1.444111	-0.087947	-1.884431
H	-2.087348	-0.003399	1.462736
H	-0.530677	0.082071	2.031600

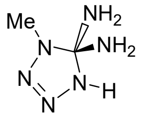

1P2b (gv_az2b_m3.log)

N	-0.629358	-0.018345	-0.182658
C	-1.369973	1.204601	0.078745
N	1.299574	-1.254803	0.064807
C	0.744682	-0.100209	-0.039590
H	2.310566	-1.178677	0.132107
N	-1.372163	-1.204630	0.039517
N	1.419696	1.123988	-0.102251
H	-2.403982	1.026365	-0.203349
H	-1.352100	1.503339	1.136924
H	-0.990148	2.022491	-0.536001
H	-1.255340	-1.789210	-0.785019
H	-0.908813	-1.723003	0.786631
H	0.948931	1.925078	0.291472
H	2.378394	1.063788	0.206403

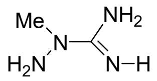

2P2b (gv_az2b_m6.log)

N	-0.779924	-1.300060	0.016124
N	-1.754599	-0.467743	0.002572
N	0.365700	-0.554271	0.007914
C	1.679412	-1.161078	-0.017357
N	-1.314147	0.819197	0.000132
C	0.002062	0.741065	0.002498
N	0.881427	1.798651	-0.078570
H	0.408950	2.687286	0.023463
H	1.696784	1.736963	0.516234

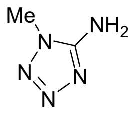

H	2.286287	-0.698372	-0.796899
H	2.180213	-1.058584	0.947913
H	1.549720	-2.217636	-0.238769

1TS3a (gv_az3a_ts1.log)

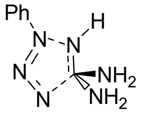

N	-0.844594	-0.948634	-1.071545
N	-1.961490	-1.181176	-0.759626
N	-0.153587	0.120249	-0.912499
C	1.172305	0.033024	-0.444168
C	-2.456158	0.288036	0.388894
N	-1.424502	1.139088	0.240609
N	-2.556571	-0.308012	1.672036
N	-3.702386	0.597483	-0.260877
H	-1.647994	1.775780	-0.520518
H	-3.014017	-1.212178	1.652560
H	-1.652950	-0.374738	2.125687
H	-4.167382	-0.244619	-0.582923
H	-4.318344	1.064675	0.398889
C	1.895185	1.226776	-0.382440
C	3.192736	1.223858	0.107706
C	3.789367	0.033578	0.515331
C	3.072315	-1.157158	0.438654
C	1.764921	-1.163913	-0.027774
H	1.424852	2.145186	-0.708369
H	3.744816	2.154319	0.162262
H	4.807400	0.032894	0.884843
H	3.532273	-2.088634	0.746556
H	1.209225	-2.090878	-0.092886

2TS3a (gv_az3a_ts3.log)

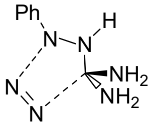

N	-1.022634	1.775593	-0.322413
N	-1.952357	1.207602	-0.749278
N	-0.320220	0.713641	0.951747
C	0.931675	0.261162	0.475816
C	-2.130021	-0.345416	-0.126136
N	-1.317642	-0.227636	1.057804
N	-1.670315	-1.137841	-1.213895
N	-3.462104	-0.680223	0.184360
H	-1.905199	0.025427	1.847529
H	-3.927373	0.015818	0.754160
H	-3.995884	-0.840335	-0.660640
H	-0.694825	-0.959933	-1.424562
H	-1.781291	-2.122754	-0.992075
C	1.314213	-1.086146	0.493584
C	2.590373	-1.456721	0.079860
C	3.500522	-0.503346	-0.360094
C	3.123363	0.838538	-0.368448
C	1.859361	1.223653	0.050875
H	0.620370	-1.829519	0.862134
H	2.872875	-2.502993	0.110426
H	4.492043	-0.797030	-0.681611
H	3.824522	1.595571	-0.700224
H	1.574751	2.267460	0.053841

3TS3a (gv_az3a_ts6.log)

N	-0.848469	1.443328	-0.046542
N	-2.126474	1.321435	-0.109962
N	-0.266857	0.260407	-0.017018

C	1.126116	0.086006	-0.017890	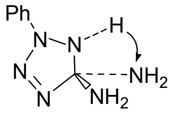
C	-2.430031	-0.101667	-0.109749	
N	-1.166320	-0.809563	-0.049380	
N	-3.377129	-0.556923	-1.088018	
N	-2.936477	-0.551537	1.305999	
H	-1.814140	-1.090487	1.189994	
H	-2.976019	0.230910	1.957320	
H	-3.809108	-1.079918	1.308163	
H	-3.996844	0.205923	-1.349474	
H	-2.867934	-0.850367	-1.913892	
C	1.650194	-1.206717	-0.078651	
C	3.029043	-1.386028	-0.074458	
C	3.887117	-0.294969	-0.008108	
C	3.352299	0.991014	0.054228	
C	1.980863	1.191901	0.051890	
H	0.975976	-2.049023	-0.136942	
H	3.430968	-2.391076	-0.124732	
H	4.960168	-0.441156	-0.004558	
H	4.010927	1.849913	0.105663	
H	1.564477	2.188016	0.099329	

4TS3a (gv_az3a_ts4.log)

N	-0.493009	-0.817423	-0.337982	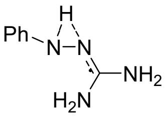
C	0.797057	-0.307736	-0.167054	
C	-2.651421	0.061620	0.018809	
N	-1.494536	0.326810	-0.560005	
N	-2.814852	-1.011315	0.816968	
N	-3.704701	0.920935	-0.160031	
H	-1.101907	-0.298342	-1.431697	
H	-3.529607	1.651890	-0.834658	
H	-4.625477	0.510106	-0.218264	
H	-1.939888	-1.523057	0.937598	
H	-3.474801	-0.963228	1.577724	
C	1.116370	1.026382	0.133374	
C	2.441656	1.409015	0.318773	
C	3.475059	0.484758	0.222487	
C	3.163970	-0.844735	-0.068272	
C	1.851192	-1.237628	-0.264882	
H	0.321907	1.754577	0.220683	
H	2.664334	2.445575	0.548256	
H	4.503649	0.789356	0.372122	
H	3.956443	-1.580848	-0.147426	
H	1.611740	-2.269142	-0.496405	

5TS3a (gv_az3a_ts5.log)

N	-0.546431	-0.667238	-0.606386	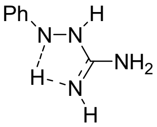
C	0.736083	-0.242725	-0.276760	
C	-2.613654	0.013998	0.102827	
N	-1.513343	0.372951	-0.586715	
N	-2.478144	-1.144526	0.704469	
N	-3.715454	0.820966	0.155618	
H	-1.619977	0.935111	-1.422863	
H	-3.708447	1.646524	-0.425097	
H	-4.612187	0.365343	0.226657	
H	-1.384037	-1.392063	0.316398	
H	-3.073132	-1.417423	1.470731	
C	1.115389	1.082063	0.005695	
C	2.439959	1.396336	0.296875	

C	3.421879	0.413970	0.323783
C	3.054116	-0.904939	0.048965
C	1.742831	-1.230138	-0.250112
H	0.368161	1.865616	0.011420
H	2.701732	2.427296	0.510790
H	4.450382	0.665067	0.551695
H	3.804026	-1.688511	0.062916
H	1.467463	-2.253425	-0.479193

1M3a (gv_az3a_m1.log)

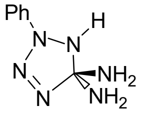

N	0.885577	1.426091	-0.007593
N	2.125668	1.289858	-0.093882
N	0.259600	0.229700	0.069282
C	-1.131569	0.073653	0.028518
C	2.471825	-0.179019	-0.014985
N	1.161380	-0.841847	-0.162191
N	3.032973	-0.515424	1.274520
N	3.365929	-0.536703	-1.077900
H	1.081439	-1.120387	-1.142576
H	3.702268	0.196200	1.556338
H	2.302853	-0.565138	1.977748
H	3.658832	0.279191	-1.601892
H	4.170649	-1.037863	-0.722575
C	-1.681639	-1.210993	0.025710
C	-3.063301	-1.365746	-0.003198
C	-3.902298	-0.258795	-0.027238
C	-3.343802	1.017883	-0.021937
C	-1.969152	1.195505	0.003856
H	-1.031463	-2.074077	0.062763
H	-3.481305	-2.365357	-0.003811
H	-4.977349	-0.385816	-0.049574
H	-3.986249	1.890219	-0.041365
H	-1.537953	2.186368	0.004940

2M3a (gv_az3a_m3.log)

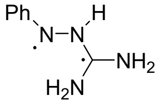

N	-0.498697	-0.775173	-0.236206
C	0.787347	-0.311802	-0.085261
C	-2.667144	0.062769	0.007030
N	-1.419979	0.237677	-0.350822
N	-3.038520	-1.151380	0.486020
N	-3.630681	1.049046	-0.105946
H	-1.163436	1.096781	-0.837157
H	-3.294738	1.939816	-0.444957
H	-4.473227	0.757008	-0.587815
H	-2.231835	-1.758014	0.622743
H	-3.741572	-1.153189	1.210958
C	1.168626	1.026905	0.165450
C	2.512351	1.384238	0.255881
C	3.517688	0.436771	0.125793
C	3.153236	-0.896763	-0.093548
C	1.827249	-1.265801	-0.199119
H	0.420830	1.790883	0.348176
H	2.768750	2.420282	0.451416
H	4.559847	0.720160	0.203421
H	3.922456	-1.655540	-0.190010
H	1.551951	-2.297274	-0.385450

1P3a (gv_az3a_m5.log)

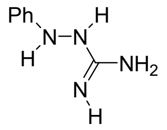

N	0.518949	-0.588706	0.944347
C	-0.739750	-0.221731	0.415018
C	2.539312	-0.072081	-0.214802
N	1.575469	0.313646	0.727872
N	2.401207	-1.163321	-0.860590
N	3.552014	0.863976	-0.358282
H	1.976650	0.615562	1.607569
H	3.554502	1.659427	0.261948
H	4.464448	0.513297	-0.601757
H	0.807707	-1.511603	0.628033
H	3.105003	-1.275138	-1.584827
C	-1.090542	1.104738	0.161581
C	-2.372097	1.412183	-0.283828
C	-3.318712	0.413561	-0.481246
C	-2.965722	-0.910095	-0.229126
C	-1.690731	-1.228148	0.214723
H	-0.357760	1.886945	0.304742
H	-2.628606	2.447120	-0.479739
H	-4.314468	0.659914	-0.828397
H	-3.687711	-1.703691	-0.383278
H	-1.423792	-2.261561	0.408359

2P3a (gv_az3a_m4.log)

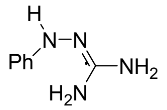

N	-0.520213	-0.850273	-0.257468
C	0.791776	-0.345111	-0.146072
C	-2.584234	0.052956	-0.028737
N	-1.499656	0.087777	-0.727590
N	-2.872318	-0.841676	0.978267
N	-3.575682	0.985491	-0.294528
H	-0.536614	-1.664296	-0.863669
H	-3.371112	1.519232	-1.129310
H	-4.514747	0.610003	-0.299021
H	-2.041137	-1.332392	1.289292
H	-3.441519	-0.472878	1.727099
C	1.039012	1.001930	0.127619
C	2.345189	1.446479	0.302685
C	3.419036	0.567551	0.211990
C	3.171283	-0.775185	-0.064806
C	1.873175	-1.229355	-0.245442
H	0.207462	1.690075	0.189032
H	2.522076	2.495716	0.510509
H	4.433186	0.921346	0.350285
H	3.995625	-1.474306	-0.146588
H	1.690438	-2.277319	-0.461822

3P3a (gv_az3a_m6.log)

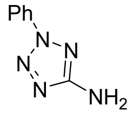

N	-1.210482	-1.385212	-0.019859
N	-2.497260	-1.152467	-0.017860
N	-0.607995	-0.207240	-0.005763
C	0.804437	-0.052291	-0.001667
C	-2.632016	0.199865	-0.003740
N	-1.465255	0.828118	0.007190
N	-3.851729	0.825922	-0.055453
H	-4.620040	0.254607	0.264366
H	-3.864714	1.767862	0.306002
C	1.350364	1.228172	-0.015733
C	2.732350	1.373503	-0.011382

C	3.560565	0.256652	0.006118
C	2.998547	-1.017111	0.019530
C	1.620563	-1.182098	0.016279
H	0.695897	2.087710	-0.030826
H	3.160525	2.368379	-0.022629
H	4.636915	0.376553	0.008911
H	3.636304	-1.892476	0.033113
H	1.175290	-2.166645	0.026852

1TS3b (gv_az3b_ts1.log)

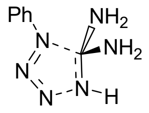

N	-0.819373	1.619366	0.095913
N	-2.049451	1.854752	-0.048809
N	-0.388411	0.398419	0.075540
C	0.996163	0.194659	0.035636
N	-2.812652	0.590882	-0.392291
C	-2.280137	-0.599235	-0.030243
H	-3.080599	0.646558	-1.368110
N	-2.419413	-0.975461	1.287521
N	-2.221857	-1.618566	-0.951813
H	-1.637080	-1.495024	1.663653
H	-2.672977	-0.198925	1.884660
H	-1.680142	-1.427069	-1.781577
H	-2.076001	-2.543342	-0.574622
C	1.453199	-1.130972	0.055064
C	2.812081	-1.416603	0.017197
C	3.746606	-0.387813	-0.034244
C	3.298781	0.932047	-0.053226
C	1.944157	1.229541	-0.025987
H	0.731405	-1.938890	0.100648
H	3.141283	-2.449399	0.031994
H	4.806448	-0.608788	-0.060497
H	4.016533	1.743662	-0.095169
H	1.604126	2.255730	-0.048587

2TS3b (gv_az3b_ts3.log)

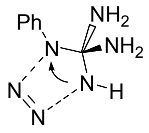

N	1.680868	1.941875	0.542449
N	2.738878	1.627512	0.325001
N	0.523254	0.382185	-0.434969
C	-0.792495	0.177063	-0.182399
N	2.787832	-0.099639	-0.589756
C	1.522559	-0.615663	-0.123127
H	3.522766	-0.502845	-0.000973
N	1.260858	-1.775558	-1.016293
N	1.641541	-1.125104	1.238386
H	0.983239	-1.435059	-1.934100
H	2.115062	-2.314738	-1.121927
H	1.893936	-0.369629	1.867174
H	0.764585	-1.509491	1.569901
C	-1.396388	-1.021460	0.292566
C	-2.773333	-1.103818	0.437716
C	-3.588708	-0.007600	0.174060
C	-3.015407	1.183079	-0.285037
C	-1.655010	1.268175	-0.484157
H	-0.799931	-1.904394	0.464737
H	-3.215282	-2.036245	0.769539
H	-4.660193	-0.078498	0.315412
H	-3.645109	2.037711	-0.502533
H	-1.199003	2.175638	-0.858676

3TS3b (gv_az3b_ts6.log)

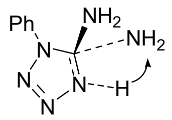

N	-1.112683	1.737727	-0.117166
N	-2.352734	1.630839	0.043437
N	-0.582938	0.417600	-0.312470
C	0.797907	0.199984	-0.154962
N	-2.808541	0.356321	-0.005256
C	-1.629715	-0.504030	-0.125429
H	-2.797270	-0.344726	1.205390
N	-1.790018	-1.087509	1.347509
N	-1.672388	-1.620641	-1.032611
H	-1.818113	-2.102937	1.431521
H	-1.094814	-0.705770	1.985364
H	-2.644521	-1.881803	-1.179890
H	-1.311500	-1.319685	-1.932627
C	1.317142	-1.099702	-0.102068
C	2.685789	-1.295453	0.045383
C	3.554375	-0.214346	0.136384
C	3.034364	1.075483	0.076820
C	1.670879	1.291908	-0.066287
H	0.657847	-1.952190	-0.198981
H	3.071534	-2.307600	0.082610
H	4.619680	-0.373155	0.247664
H	3.697151	1.930282	0.143906
H	1.270660	2.294158	-0.108100

1M3b (gv_az3b_m1.log)

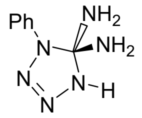

N	-1.102086	1.690575	-0.220966
N	-2.349290	1.667845	-0.146104
N	-0.596652	0.424236	-0.034434
C	0.787419	0.211852	-0.031788
N	-2.779380	0.397635	0.195871
C	-1.690506	-0.577020	0.069842
H	-3.642814	0.142082	-0.265660
N	-1.690879	-1.421638	1.241803
N	-1.826518	-1.419393	-1.098205
H	-0.815073	-1.921488	1.351350
H	-1.848446	-0.856294	2.067861
H	-1.283166	-1.107903	-1.891903
H	-1.691344	-2.402415	-0.908310
C	1.327626	-1.074755	-0.156776
C	2.705424	-1.262832	-0.124633
C	3.567944	-0.184083	0.017898
C	3.030779	1.095897	0.131319
C	1.659704	1.300595	0.114125
H	0.685226	-1.931017	-0.307140
H	3.101356	-2.266802	-0.223287
H	4.639762	-0.335498	0.038136
H	3.687338	1.950874	0.242449
H	1.250468	2.295729	0.210832

2P3b (gv_az3b_m6.log)

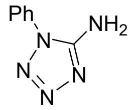

N	-1.474342	-1.443431	-0.440717
N	-2.730662	-1.227284	-0.356318
N	-0.853081	-0.260855	-0.101658
C	0.562137	-0.143415	-0.060862
N	-3.002285	0.044944	0.040477
C	-1.830535	0.627510	0.194639
N	-1.633923	1.938918	0.549111

H	-2.467400	2.358135	0.938593
H	-0.803767	2.128575	1.094049
C	1.183991	0.996314	-0.567092
C	2.570101	1.096009	-0.516371
C	3.329765	0.060174	0.016077
C	2.699092	-1.081694	0.502153
C	1.314860	-1.187285	0.472939
H	0.591464	1.784886	-1.012923
H	3.055413	1.980400	-0.910678
H	4.409446	0.139115	0.046807
H	3.286531	-1.894059	0.912075
H	0.811895	-2.068772	0.846910

3P3b (gv_az3b_m3.log)

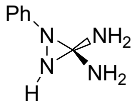

N	-0.911870	0.237523	-0.792405
C	0.450044	0.113728	-0.396582
N	-1.653610	1.358475	-0.105559
C	-1.986673	-0.030477	0.149148
H	-2.271037	1.744465	-0.820298
N	-1.679169	-0.612485	1.406860
N	-3.227728	-0.529829	-0.337894
H	-0.817336	-0.236176	1.784827
H	-2.432144	-0.435779	2.063456
H	-3.345068	-0.308639	-1.320551
H	-3.259594	-1.538601	-0.229915
C	1.043829	-1.148139	-0.484145
C	2.390037	-1.303818	-0.181215
C	3.159503	-0.206834	0.198606
C	2.566479	1.048656	0.277860
C	1.215787	1.213613	-0.012836
H	0.446087	-1.996792	-0.793467
H	2.841073	-2.287091	-0.245991
H	4.210688	-0.330768	0.428641
H	3.156479	1.909318	0.571112
H	0.743455	2.183904	0.060153

1TS4a (gv_az4e_ts1.log)

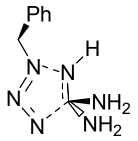

N	-1.147198	1.360684	-0.822061
N	-1.795946	0.555150	-1.388551
N	-0.959199	1.577979	0.420872
C	0.424390	1.776974	0.895592
C	-2.238985	-0.719817	0.076360
N	-1.815232	-0.049377	1.158003
N	-1.488258	-1.869980	-0.265227
N	-3.650675	-0.783288	-0.191839
H	-2.595752	0.493232	1.518673
H	0.330437	1.778605	1.980570
C	1.430005	0.744886	0.442699
H	0.733491	2.781639	0.595220
C	2.151944	0.926253	-0.737850
C	3.070209	-0.028663	-1.162429
C	3.280451	-1.177360	-0.406807
C	2.568842	-1.365928	0.774611
C	1.648619	-0.411277	1.195327
H	1.996248	1.821748	-1.329696
H	3.623806	0.126851	-2.080804
H	3.998650	-1.919812	-0.733876
H	2.732894	-2.255517	1.371384

H	1.088250	-0.563520	2.110146
H	-1.548768	-2.095355	-1.251494
H	-0.517183	-1.781202	0.013066
H	-3.839551	-0.728735	-1.186852
H	-4.019800	-1.666515	0.150265

2TS4a (gv_az4e_ts3.log)

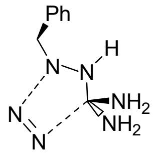

N	1.579998	1.308403	1.201141
N	1.984749	0.241943	1.416215
N	1.285210	1.452559	-0.577248
C	-0.133072	1.784752	-0.763735
C	1.905735	-0.822388	-0.106739
N	1.767328	0.246245	-1.025046
N	0.815189	-1.675019	0.082190
N	3.122300	-1.510030	-0.223366
H	2.683530	0.398215	-1.434727
H	-0.265286	2.033576	-1.821797
C	-1.226618	0.818217	-0.352307
H	-0.250799	2.717989	-0.207807
H	0.837214	-2.132962	0.986370
H	-0.078703	-1.222557	-0.065622
H	3.878669	-1.107113	0.313572
H	3.040670	-2.506764	-0.080332
C	-1.662907	0.741375	0.974798
C	-2.675446	-0.137852	1.342460
C	-3.274147	-0.955804	0.387133
C	-2.857517	-0.883286	-0.937839
C	-1.844647	0.000190	-1.302323
H	-1.201099	1.374936	1.723227
H	-3.002313	-0.181125	2.374904
H	-4.064668	-1.639348	0.673317
H	-3.322302	-1.510664	-1.689309
H	-1.526619	0.055885	-2.337684

3TS4a (gv_az4e_ts6.log)

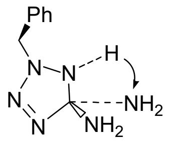

N	-1.384713	-1.092664	1.091456
N	-2.292346	-0.195424	1.277181
N	-0.893352	-1.015605	-0.124107
C	0.387135	-1.623381	-0.456893
C	-2.394297	0.574341	0.052861
N	-1.406558	0.048065	-0.870226
N	-2.460394	2.003399	0.181109
N	-3.654750	0.117355	-0.788109
H	-2.668048	-0.243938	-1.464591
H	-4.163705	-0.632196	-0.322362
H	-4.283070	0.866337	-1.078821
H	-2.861392	2.246646	1.083761
H	-1.514072	2.366195	0.150004
H	0.342494	-1.930578	-1.502858
C	1.574472	-0.710822	-0.227363
H	0.457672	-2.518956	0.160840
C	2.003632	-0.428595	1.070689
C	3.089333	0.411469	1.287480
C	3.760678	0.978600	0.207328
C	3.339061	0.702586	-1.088417
C	2.249908	-0.137073	-1.302239
H	1.478870	-0.864463	1.913659
H	3.414664	0.621239	2.299683

H	4.608589	1.631867	0.376267
H	3.855148	1.142594	-1.933642
H	1.918103	-0.343376	-2.313741

4TS4a (gv_az4e_ts4b.log)

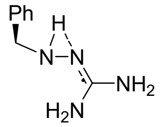

N	1.171456	1.533027	0.047800
C	-0.065941	1.747130	-0.701522
C	2.498702	-0.432770	0.195786
N	1.911957	0.373744	-0.668192
N	2.289429	-0.292855	1.518848
N	3.293315	-1.458282	-0.252113
H	2.247769	1.420174	-0.851009
H	0.103800	1.756671	-1.788647
C	-1.146499	0.733251	-0.378852
H	-0.406724	2.747987	-0.418155
H	2.285908	-1.118636	2.098663
H	1.596905	0.448871	1.679207
H	3.462421	-1.442776	-1.247628
H	4.106086	-1.679961	0.304942
C	-1.940074	0.880562	0.762084
C	-2.901613	-0.067033	1.093620
C	-3.084899	-1.187538	0.286810
C	-2.303091	-1.346366	-0.852371
C	-1.344501	-0.391354	-1.181421
H	-1.800617	1.750816	1.395170
H	-3.513372	0.070069	1.978263
H	-3.835544	-1.926641	0.541623
H	-2.442377	-2.212865	-1.489098
H	-0.739854	-0.518447	-2.072537

5TS4a (gv_az4e_ts5.log)

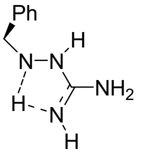

N	1.155336	-1.045346	-0.398793
C	0.071151	-1.351187	0.521559
C	2.893216	0.432047	-0.043433
N	1.575832	0.299609	-0.197741
N	3.523682	-0.730776	0.005505
N	3.494017	1.667245	-0.049348
H	1.034492	1.063164	-0.587534
H	0.339690	-1.195464	1.582218
C	-1.227466	-0.598780	0.252836
H	-0.114660	-2.424434	0.401340
H	4.434751	-0.822965	0.426059
H	2.614950	-1.421714	-0.085420
H	2.949183	2.425244	0.337299
H	4.464389	1.688043	0.222694
C	-1.707333	-0.461100	-1.053043
C	-2.895883	0.213451	-1.308118
C	-3.627607	0.768902	-0.260329
C	-3.158034	0.643854	1.042121
C	-1.966071	-0.033051	1.292003
H	-1.128172	-0.886005	-1.864988
H	-3.255630	0.305469	-2.326986
H	-4.553542	1.295912	-0.459441
H	-3.716286	1.076645	1.864784
H	-1.603075	-0.123840	2.311034

1M4a (gv_az4e_m1.log)

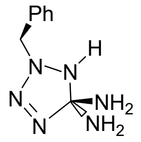

N	1.371545	1.255053	0.972915
N	2.019521	0.230781	1.242580
N	1.082541	1.365723	-0.365356
C	-0.294410	1.812874	-0.652368
C	2.132933	-0.650396	0.021309
N	1.526384	0.177942	-1.036798
N	1.383533	-1.872441	0.182269
N	3.519316	-0.941149	-0.248685
H	2.284109	0.456060	-1.653944
H	-0.323585	2.071467	-1.710624
C	-1.374815	0.808725	-0.317087
H	-0.423580	2.730812	-0.078126
C	-1.881333	0.720531	0.981849
C	-2.860068	-0.215000	1.299043
C	-3.345588	-1.076632	0.319594
C	-2.848431	-0.996809	-0.977255
C	-1.869457	-0.059278	-1.291895
H	-1.502193	1.385882	1.749271
H	-3.245945	-0.269159	2.310169
H	-4.109895	-1.804333	0.565421
H	-3.222844	-1.663584	-1.744993
H	-1.481548	-0.003523	-2.302391
H	1.576308	-2.287917	1.089871
H	0.388025	-1.686177	0.115176
H	4.121390	-0.566394	0.474917
H	3.666888	-1.938592	-0.342361

2M4a (gv_az4e_m3.log)

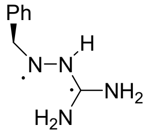

N	-1.195755	-1.270831	0.016207
C	0.060760	-1.596256	-0.642284
C	-2.745890	0.473874	0.019733
N	-1.551638	0.002556	-0.237199
N	-3.643366	-0.371979	0.629946
N	-2.998393	1.837190	-0.202423
H	-0.873535	0.692734	-0.565537
H	-0.028167	-1.602291	-1.743511
C	1.242581	-0.708927	-0.268671
H	0.279977	-2.626282	-0.350579
H	-4.614761	-0.236778	0.387830
H	-3.317924	-1.337032	0.605273
H	-3.635367	2.013052	-0.973292
H	-3.343947	2.312893	0.626100
C	1.612784	-0.554318	1.071616
C	2.687102	0.251329	1.424383
C	3.409679	0.928501	0.442451
C	3.047651	0.791238	-0.891160
C	1.970167	-0.022540	-1.240472
H	1.040770	-1.069176	1.835279
H	2.965327	0.353519	2.467233
H	4.246498	1.559160	0.718986
H	3.599879	1.316778	-1.661895
H	1.696317	-0.132518	-2.285180

1P4a (gv_az4e_m5.log)

N	1.076494	-1.234463	-0.192164
C	0.122872	-1.015519	0.903957
C	2.920418	0.308072	-0.145937

N	1.653721	-0.013906	-0.624076
N	3.472235	-0.406945	0.758679
N	3.457432	1.417989	-0.801012
H	1.551008	0.112865	-1.622040
H	0.552710	-0.395618	1.699459
C	-1.183563	-0.413324	0.435171
H	-0.065153	-2.005946	1.332703
H	4.358997	-0.019895	1.068507
H	1.842972	-1.807028	0.158414
H	2.787941	2.143754	-1.021857
H	4.293529	1.788284	-0.373955
C	-1.841722	-0.917472	-0.689023
C	-3.059631	-0.386024	-1.095796
C	-3.641881	0.659928	-0.383567
C	-2.994435	1.170025	0.735476
C	-1.772383	0.636686	1.137439
H	-1.385318	-1.726078	-1.247614
H	-3.558173	-0.789149	-1.969947
H	-4.591561	1.074179	-0.701168
H	-3.436038	1.987313	1.293945
H	-1.268145	1.044355	2.007241

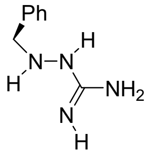

2P4a (gv_az4e_m4.log)

N	1.265250	1.503609	0.057095
C	0.004472	1.781693	-0.654367
C	2.378802	-0.477931	0.143458
N	1.995303	0.467100	-0.644529
N	2.273321	-0.479019	1.517907
N	2.965282	-1.611695	-0.403852
H	1.843991	2.336128	-0.024820
H	0.171964	1.819795	-1.737884
C	-1.067160	0.763341	-0.340772
H	-0.325565	2.771833	-0.326149
H	2.075036	-1.382246	1.925138
H	1.653645	0.262526	1.827921
H	3.143970	-1.496065	-1.392856
H	3.780221	-1.939647	0.097712
C	-1.747793	0.804462	0.878496
C	-2.727660	-0.133601	1.180672
C	-3.042479	-1.133045	0.263204
C	-2.372007	-1.184186	-0.953524
C	-1.392099	-0.240769	-1.251305
H	-1.506156	1.581524	1.596110
H	-3.250554	-0.083084	2.128887
H	-3.807866	-1.864265	0.495532
H	-2.610357	-1.959252	-1.672712
H	-0.866882	-0.287002	-2.198393

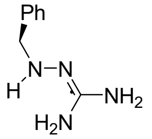

3P4a (gv_az4e_m6.log)

N	1.845481	-1.420560	0.140345
N	2.868071	-0.894058	-0.494208
N	1.168543	-0.419383	0.659911
C	-0.076194	-0.605278	1.400580
C	2.757423	0.446111	-0.324307
N	1.692806	0.783008	0.396398
N	3.688860	1.341312	-0.795371
H	4.232882	0.989096	-1.569628
H	3.336983	2.275658	-0.943292

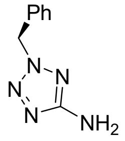

H	-0.011441	0.005843	2.300364
C	-1.307184	-0.249616	0.596655
H	-0.082522	-1.655526	1.690305
C	-1.720990	-1.067974	-0.455721
C	-2.853042	-0.745164	-1.193495
C	-3.586112	0.397589	-0.884031
C	-3.180509	1.215713	0.163979
C	-2.043899	0.893001	0.899387
H	-1.151042	-1.957653	-0.700031
H	-3.166184	-1.387208	-2.008130
H	-4.470510	0.647407	-1.457929
H	-3.746033	2.106753	0.409162
H	-1.725426	1.537116	1.711365

TS4b (gv_az4h_ts2.log)

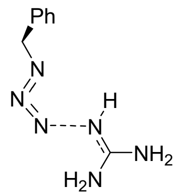

N	-0.949914	-1.413042	0.557848
N	-2.114600	-1.120363	0.787333
N	-0.172395	-0.961870	-0.346283
C	1.162197	-1.560879	-0.303978
N	-2.607058	0.101011	-0.360914
C	-3.791219	0.584755	-0.241838
H	-1.894610	0.497424	-0.957829
N	-4.716558	-0.075220	0.514604
N	-4.214164	1.756176	-0.830143
H	1.337447	-2.089330	-1.247823
C	2.260588	-0.531296	-0.120255
H	1.219785	-2.306254	0.498717
C	2.139080	0.485393	0.829407
C	3.163488	1.405043	1.022498
C	4.329769	1.326436	0.265568
C	4.460012	0.321641	-0.686474
C	3.430428	-0.595936	-0.876960
H	1.230344	0.554945	1.415272
H	3.052563	2.186306	1.766089
H	5.127701	2.044447	0.415054
H	5.360516	0.254332	-1.286327
H	3.536098	-1.372934	-1.627177
H	-4.314284	-0.827329	1.062263
H	-5.395698	0.493268	0.997181
H	-3.581186	2.165779	-1.500542
H	-5.181909	1.781576	-1.119804

1TS4b (gv_az4h_ts1.log)

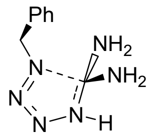

N	1.588352	-1.608792	0.348718
N	2.516471	-1.559876	-0.518078
N	1.011089	-0.537696	0.761775
C	-0.228604	-0.754881	1.506674
N	2.636612	-0.167521	-1.103333
C	2.241478	0.889574	-0.374016
H	2.271585	-0.186781	-2.048375
N	3.054264	1.293709	0.661818
N	1.447802	1.855001	-0.948410
H	-0.206361	-0.149141	2.418200
C	-1.454025	-0.372352	0.696683
H	-0.288557	-1.806105	1.807364
C	-1.740553	-1.027466	-0.505120
C	-2.855068	-0.676808	-1.257219
C	-3.704009	0.339496	-0.822270

C	-3.429467	0.997574	0.370383
C	-2.310744	0.642193	1.121606
H	-1.083816	-1.819697	-0.847045
H	-3.067078	-1.200515	-2.182321
H	-4.573639	0.611746	-1.408560
H	-4.084777	1.787589	0.718811
H	-2.104262	1.157888	2.053911
H	3.742472	0.590666	0.900105
H	2.552954	1.610014	1.481798
H	0.554465	1.529961	-1.289369
H	1.390842	2.726633	-0.442272

2TS4b (gv_az4h_ts3.log)

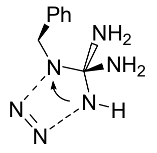

N	-1.784856	2.083603	0.323211
N	-2.718951	1.941988	-0.266195
N	-1.280622	-0.009855	0.961530
C	-0.032716	-0.529590	1.433347
N	-3.125920	-0.180421	-0.464959
C	-1.769935	-0.572031	-0.268382
H	-3.276479	-0.081669	-1.474213
N	-1.950867	-2.040693	0.042080
N	-0.989820	-0.501827	-1.496826
H	-0.138906	-1.635593	1.451867
C	1.254524	-0.209658	0.674002
H	0.084219	-0.205384	2.471803
C	1.632697	1.118961	0.463303
C	2.822931	1.420102	-0.187797
C	3.657601	0.398331	-0.634859
C	3.294103	-0.926042	-0.422646
C	2.099594	-1.225941	0.228893
H	0.986334	1.917832	0.806998
H	3.102371	2.455505	-0.344690
H	4.585246	0.634902	-1.142154
H	3.938247	-1.728551	-0.762691
H	1.822678	-2.262327	0.391488
H	-2.586724	-2.439202	-0.643315
H	-2.384473	-2.128716	0.960546
H	-0.865260	0.469261	-1.766283
H	-0.062796	-0.890407	-1.356398

3TS4b (gv_az4h_ts6.log)

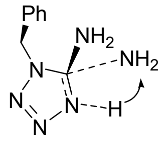

N	-1.418808	1.683600	0.394356
N	-2.411466	1.746373	-0.392093
N	-0.990813	0.375052	0.422089
C	0.102228	-0.043641	1.293517
N	-2.638412	0.525205	-0.949593
C	-1.800905	-0.388827	-0.337618
H	-3.632106	0.088020	-0.853645
N	-3.829917	-1.121853	0.306847
N	-1.377577	-1.636808	-0.768672
H	-4.258451	-2.030751	0.498031
H	-4.017783	-0.579809	1.153262
H	-2.160061	-2.193673	-1.082962
H	-0.632125	-1.581781	-1.459271
H	-0.147134	-1.042522	1.649851
C	1.457094	-0.041934	0.616839
H	0.087606	0.641212	2.142535
C	2.016426	1.149785	0.150391

C	3.264650	1.148063	-0.459675
C	3.971359	-0.043157	-0.604726
C	3.422655	-1.232154	-0.138991
C	2.169029	-1.230482	0.466787
H	1.470546	2.079855	0.262596
H	3.689056	2.078033	-0.818635
H	4.945999	-0.042428	-1.077835
H	3.967427	-2.162436	-0.247249
H	1.740762	-2.160611	0.823650

1M4b (gv_az4h_m2.log)

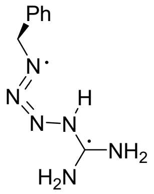

N	-0.985362	-1.482917	0.514911
N	-2.193593	-1.021338	0.712654
N	-0.266920	-0.947880	-0.398579
C	1.060274	-1.544657	-0.496312
N	-2.456415	0.061824	-0.172224
C	-3.621716	0.631279	-0.183271
H	-1.646503	0.277749	-0.772045
N	-4.635822	0.086820	0.534041
N	-3.869312	1.788920	-0.881804
H	1.198811	-1.921923	-1.515278
C	2.166185	-0.552303	-0.193227
H	1.129113	-2.400158	0.186164
C	2.166763	0.164103	1.006529
C	3.189755	1.056980	1.301270
C	4.232235	1.253266	0.397936
C	4.240545	0.549129	-0.800228
C	3.212384	-0.344681	-1.090857
H	1.355426	0.017239	1.710058
H	3.176458	1.601135	2.238944
H	5.029835	1.950156	0.627718
H	5.044970	0.696305	-1.511953
H	3.223224	-0.888866	-2.029626
H	-4.331632	-0.677262	1.129668
H	-5.318728	0.718139	0.923564
H	-3.114524	2.114710	-1.468335
H	-4.773038	1.836083	-1.332919

2M4b (gv_az4h_m1.log)

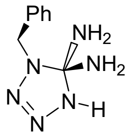

N	-1.510473	1.516753	0.664078
N	-2.400379	1.732425	-0.188381
N	-1.268533	0.166993	0.764181
C	-0.049358	-0.251823	1.433280
N	-2.852310	0.523061	-0.700915
C	-1.924835	-0.558451	-0.352097
H	-3.085639	0.589171	-1.683480
N	-2.685319	-1.728496	0.031600
N	-1.060399	-0.940811	-1.445522
H	-0.174120	-1.299855	1.717375
C	1.260562	-0.088507	0.675472
H	-0.007047	0.318933	2.363773
C	1.641926	1.151300	0.154591
C	2.853117	1.292554	-0.514288
C	3.705984	0.202385	-0.663076
C	3.340177	-1.032175	-0.138605
C	2.124259	-1.173574	0.523698
H	0.985424	2.004863	0.273434
H	3.134164	2.259267	-0.915228

H	4.649946	0.315768	-1.182560
H	3.998052	-1.886445	-0.247262
H	1.844311	-2.141474	0.927106
H	-3.404266	-1.458772	0.693193
H	-2.093355	-2.432102	0.462360
H	-0.166084	-0.469334	-1.447351
H	-0.950493	-1.939756	-1.550499

2P4b (gv_az4h_m6.log)

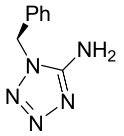

N	2.320217	-1.174012	0.620445
N	3.245941	-0.929730	-0.231332
N	1.438985	-0.130427	0.544345
C	0.265525	-0.042990	1.399573
N	3.020879	0.236315	-0.895847
C	1.896377	0.714661	-0.399685
N	1.307809	1.916392	-0.718191
H	1.737731	2.344411	-1.527404
H	0.298097	1.896089	-0.786345
H	0.320156	0.884734	1.973905
C	-1.042931	-0.111428	0.638142
H	0.356894	-0.874091	2.100230
C	-1.282290	-1.140272	-0.275947
C	-2.494004	-1.211396	-0.951294
C	-3.482625	-0.257543	-0.720580
C	-3.252064	0.768905	0.187155
C	-2.035150	0.842171	0.860804
H	-0.516227	-1.885349	-0.458940
H	-2.668866	-2.014006	-1.657626
H	-4.427045	-0.315364	-1.248081
H	-4.015303	1.515521	0.370951
H	-1.859287	1.645647	1.568353

3P4b (gv_az4h_m3.log)

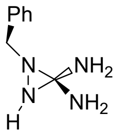

N	-1.315242	-0.819342	0.285785
C	-0.331207	-1.019501	-0.790184
N	-2.728776	-1.143664	-0.173204
C	-2.328240	0.203242	0.166768
H	-3.173679	-1.599746	0.623371
N	-2.277036	1.184213	-0.872836
N	-2.821832	0.793996	1.372477
H	-0.657484	-0.592816	-1.744193
C	1.007222	-0.442102	-0.388569
H	-0.245075	-2.098051	-0.932802
C	2.014029	-1.267748	0.111194
C	3.245567	-0.741520	0.488359
C	3.483973	0.623634	0.372067
C	2.484850	1.457010	-0.122446
C	1.255703	0.927636	-0.500128
H	1.832589	-2.332936	0.205001
H	4.018296	-1.397771	0.871485
H	4.442903	1.035905	0.663335
H	2.664518	2.521757	-0.216759
H	0.475473	1.579105	-0.877275
H	-3.116538	1.753308	-0.820802
H	-2.279759	0.733538	-1.780459
H	-2.621514	0.180197	2.155780
H	-2.330909	1.667185	1.545387

**1TS5a**	(gv_az7a_ts1.log)		
N	-1.844437	-0.346633	-1.354800
N	-2.969855	-0.625024	-1.136046
N	-1.097806	0.508568	-0.733076
C	0.234945	0.214401	-0.451231
C	-3.352470	0.148367	0.593298
N	-2.256514	0.898255	0.825156
N	-3.492208	-0.980407	1.434015
N	-4.570193	0.813178	0.218001
H	-2.435920	1.840472	0.485851
H	-4.041345	-1.722628	1.016752
H	-2.598749	-1.332172	1.756497
H	-5.099165	0.264466	-0.451344
H	-5.148753	0.951349	1.042031
C	1.103123	1.292981	-0.244684
C	2.431042	1.100314	0.099227
C	2.930371	-0.188657	0.229377
C	2.092463	-1.278242	0.027791
C	0.760580	-1.073484	-0.289295
F	0.652725	2.539602	-0.379913
F	3.236017	2.145918	0.291316
F	4.207632	-0.381103	0.550035
F	2.570648	-2.516708	0.160939
F	-0.029168	-2.140057	-0.445648

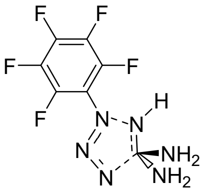

**2TS5a**	(gv_az7a_ts3.log)		
N	-1.795323	-1.685431	0.671237
N	-2.785385	-1.135571	0.986872
N	-1.170174	-0.822726	-0.715528
C	0.100471	-0.327467	-0.389629
C	-3.103985	0.223158	0.138654
N	-2.196743	0.058906	-0.985521
N	-2.854443	1.246628	1.097775
N	-4.437129	0.316848	-0.310972
H	-2.718628	-0.320655	-1.770921
H	-4.768546	-0.521693	-0.771826
H	-5.057775	0.563285	0.449360
H	-1.910314	1.205727	1.461823
H	-2.988714	2.155543	0.665191
C	0.505378	1.011320	-0.299856
C	1.826754	1.365658	-0.054931
C	2.793478	0.394353	0.133724
C	2.425828	-0.942194	0.055506
C	1.113293	-1.287079	-0.210336
F	-0.366146	2.022131	-0.459743
F	2.160033	2.658918	0.015539
F	4.059391	0.735376	0.387280
F	3.348021	-1.894182	0.219495
F	0.815482	-2.584384	-0.326957

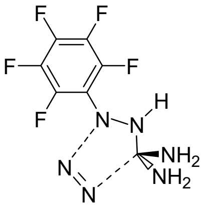

**3TS5a**	(gv_az7a_ts6.log)		
N	-1.980992	-1.164602	-1.281279
N	-3.207852	-1.231923	-1.033972
N	-1.415436	-0.233880	-0.330404
C	-0.033533	-0.123130	-0.186333
N	-3.609625	-0.494183	0.023624
C	-2.433152	0.237618	0.518925
H	-3.958405	0.849028	-0.161331

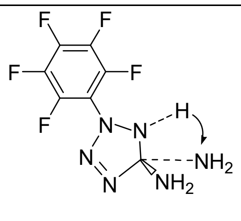

N	-3.038176	1.643782	0.157342
N	-2.097253	0.254232	1.916617
H	-3.086345	2.334534	0.906796
H	-2.549578	2.046632	-0.639264
H	-2.932585	0.430794	2.469523
H	-1.772614	-0.671866	2.180882
C	0.603507	1.117080	-0.263440
C	1.976283	1.250832	-0.147351
C	2.761674	0.119544	0.025803
C	2.161148	-1.129685	0.096815
C	0.781414	-1.243440	0.007255
F	-0.131070	2.225296	-0.466147
F	2.546977	2.455681	-0.221311
F	4.085190	0.233917	0.130446
F	2.913467	-2.215655	0.282962
F	0.234416	-2.452131	0.137366

4TS5a (gv_az7a_ts5.log)

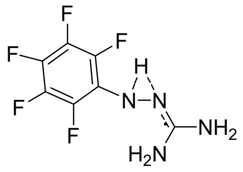

N	-1.383759	-1.010094	0.276758
C	-0.106592	-0.478070	0.113267
C	-3.525430	-0.068649	-0.133795
N	-2.352781	-0.288528	-0.704629
N	-3.740597	-0.319418	1.168410
N	-4.528696	0.476262	-0.881950
H	-1.954249	-1.346789	-0.889254
H	-4.319493	0.576436	-1.864329
H	-5.476907	0.206570	-0.667247
H	-2.898333	-0.638886	1.644639
H	-4.425211	0.220535	1.673921
C	0.193177	0.893483	0.123515
C	1.490078	1.371150	0.029013
C	2.554193	0.484970	-0.051273
C	2.300289	-0.879134	-0.040888
C	0.996903	-1.342206	0.030745
F	-0.795367	1.791688	0.279251
F	1.725379	2.690256	0.046803
F	3.810682	0.940113	-0.132053
F	3.319320	-1.743919	-0.127487
F	0.795464	-2.668658	0.007780

5TS5a (gv_az7a_ts4.log)

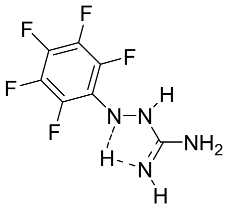

N	-1.493946	0.387114	0.725642
C	-0.185145	0.147985	0.366577
C	-3.526451	0.040440	-0.246194
N	-2.428762	-0.576456	0.252996
N	-3.395485	1.340307	-0.306031
N	-4.601265	-0.687961	-0.658616
H	-2.540981	-1.428047	0.786987
H	-4.593114	-1.681218	-0.482738
H	-5.507477	-0.249668	-0.616705
H	-2.297153	1.405040	0.185893
H	-4.014233	1.914929	-0.855360
C	0.422433	-1.110117	0.228657
C	1.770053	-1.267209	-0.053382
C	2.589613	-0.159151	-0.193973
C	2.031819	1.104574	-0.049923
C	0.682405	1.244837	0.215476
F	-0.309588	-2.234267	0.427311

F	2.288471	-2.498048	-0.167062
F	3.895889	-0.302944	-0.461857
F	2.802836	2.190117	-0.197065
F	0.175814	2.488450	0.318736

1M5a (gv_az7a_m1.log)

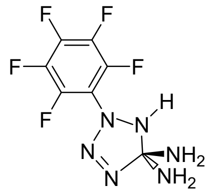

N	-1.801604	-0.440867	1.408057
N	-2.970840	-0.088227	1.225963
N	-1.163448	-0.759414	0.210934
C	0.195543	-0.359070	0.113422
C	-3.278584	0.039052	-0.249948
N	-2.028988	-0.431681	-0.889617
N	-3.531596	1.405961	-0.616099
N	-4.401794	-0.791076	-0.592964
H	-2.257841	-1.309685	-1.348310
H	-4.208767	1.826587	0.014062
H	-2.674888	1.947591	-0.588041
H	-4.782574	-1.265924	0.216707
H	-5.123049	-0.258395	-1.062911
C	1.189637	-1.329926	0.015956
C	2.530305	-0.981439	-0.083260
C	2.894049	0.357131	-0.089120
C	1.920592	1.344485	0.004414
C	0.589011	0.978915	0.105807
F	0.868757	-2.622558	0.009753
F	3.468307	-1.925028	-0.175662
F	4.176696	0.697879	-0.184284
F	2.275444	2.630474	0.001007
F	-0.334572	1.941685	0.198180

2M5a (gv_az7a_m3.log)

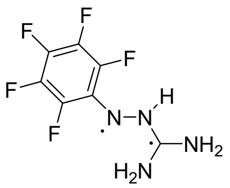

N	-1.441884	0.674231	0.021391
C	-0.148771	0.271188	0.008927
C	-3.662039	0.005654	-0.002636
N	-2.395153	-0.325525	0.014305
N	-3.998275	1.309723	0.121166
N	-4.663777	-0.928823	-0.159798
H	-2.129433	-1.304390	0.047596
H	-4.357650	-1.889133	-0.230880
H	-5.442758	-0.821225	0.478318
H	-3.187962	1.923567	0.086669
H	-4.829809	1.619007	-0.358224
C	0.401659	-1.029556	0.014059
C	1.762230	-1.293084	0.007169
C	2.677336	-0.257701	-0.005677
C	2.189818	1.047017	-0.011357
C	0.835120	1.296570	-0.004263
F	-0.431590	-2.124336	0.030178
F	2.187111	-2.565594	0.013866
F	3.999032	-0.499182	-0.012502
F	3.055545	2.070391	-0.024967
F	0.424250	2.576988	-0.011048

1P5a (gv_az7a_m4.log)

N	-1.487245	0.207445	1.044942
C	-0.179156	0.087444	0.546968
C	-3.391020	0.121728	-0.386239
N	-2.451739	-0.587875	0.377500

N	-3.290989	1.388507	-0.494166
N	-4.319193	-0.706109	-0.988755
H	-2.868729	-1.249318	1.020708
H	-4.286787	-1.694098	-0.790290
H	-5.236249	-0.327299	-1.159933
H	-1.805950	1.176488	1.016238
H	-3.954795	1.781047	-1.154831
C	0.438427	-1.149398	0.350711
C	1.754313	-1.247951	-0.074654
C	2.506843	-0.102829	-0.290894
C	1.927032	1.138862	-0.078001
C	0.604916	1.221044	0.328318
F	-0.229408	-2.285391	0.606974
F	2.308430	-2.450894	-0.255974
F	3.778028	-0.195872	-0.692539
F	2.637837	2.251693	-0.281816
F	0.063840	2.434463	0.524933

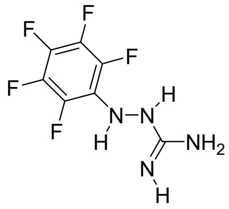

2P5a (gv_az7a_m5.log)

N	-1.397945	-1.019087	0.322830
C	-0.106612	-0.496871	0.158139
C	-3.423667	-0.106478	-0.178373
N	-2.332404	-0.571092	-0.694452
N	-3.775431	-0.136638	1.148967
N	-4.348559	0.476381	-1.024479
H	-1.357757	-2.031696	0.272519
H	-4.099842	0.364373	-1.998266
H	-5.311705	0.235335	-0.834225
H	-2.994328	-0.368690	1.749860
H	-4.326798	0.647579	1.466699
C	0.143488	0.877535	0.151480
C	1.428357	1.383047	0.028182
C	2.513166	0.522913	-0.071719
C	2.296998	-0.846960	-0.051144
C	1.004901	-1.334204	0.053201
F	-0.866771	1.744647	0.306389
F	1.629264	2.704537	0.029941
F	3.753163	1.009800	-0.179012
F	3.329918	-1.689314	-0.151093
F	0.813428	-2.668308	0.053192

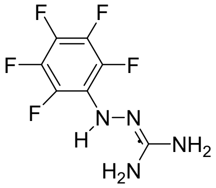

3P5a (gv_az7a_m6.log)

N	2.291627	-0.989226	-1.132213
N	3.539595	-0.878716	-0.909601
N	1.666093	-0.163394	-0.208992
C	0.265605	-0.092806	-0.117294
N	3.809517	-0.011946	0.105233
C	2.643250	0.424178	0.529384
N	2.429748	1.260985	1.591345
H	3.295055	1.632213	1.960833
H	1.729866	1.977981	1.456556
C	-0.496313	-1.241756	0.103434
C	-1.881544	-1.177749	0.154623
C	-2.526570	0.043161	0.005211
C	-1.785747	1.199520	-0.198854
C	-0.404414	1.119616	-0.265771
F	0.096510	-2.416171	0.282029
F	-2.595357	-2.281214	0.364532

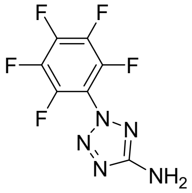

F	-3.852518	0.106682	0.066048
F	-2.400832	2.373253	-0.335636
F	0.300355	2.241996	-0.466106

TS5b (gv_az7b_ts2.log)

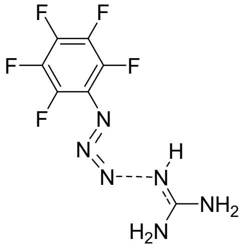

N	-1.440706	-1.082359	0.147517
N	-2.634967	-1.256181	0.156248
N	-0.812590	0.034995	-0.034526
C	0.575025	-0.032474	-0.004133
N	-3.439470	0.275420	-0.119613
C	-4.727149	0.279393	-0.046505
H	-2.884808	1.119575	-0.103332
N	-5.414958	-0.891028	-0.147555
N	-5.477699	1.413985	0.130178
H	-6.293222	-0.953737	0.343609
H	-4.830732	-1.716624	-0.104736
H	-4.986880	2.293005	0.069597
H	-6.394272	1.423039	-0.293886
C	1.249450	1.200865	-0.003927
C	2.628859	1.302853	-0.002531
C	3.410028	0.156884	-0.001646
C	2.783339	-1.078785	-0.005950
C	1.398999	-1.171290	-0.009422
F	0.536603	2.340034	-0.001328
F	3.213770	2.507836	0.003387
F	4.746211	0.243579	0.002307
F	3.523441	-2.195105	-0.013371
F	0.871233	-2.404539	-0.034584

1TS5b (gv_az7b_ts1.log)

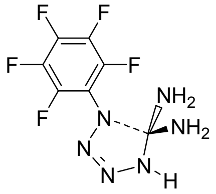

N	1.781682	1.601102	-0.620012
N	3.003198	1.802413	-0.442821
N	1.299518	0.419553	-0.305090
C	-0.071597	0.272809	-0.195979
N	3.676025	0.663355	0.251137
C	3.045127	-0.550426	0.317887
H	4.011722	1.010029	1.141791
N	3.244723	-1.416074	-0.729691
N	2.859400	-1.127118	1.550079
H	2.462500	-2.026209	-0.931225
H	3.595495	-0.953716	-1.557355
H	2.346093	-0.578643	2.223765
H	2.617523	-2.107547	1.549234
C	-0.589290	-1.031738	-0.220609
C	-1.936332	-1.317467	-0.097799
C	-2.846868	-0.282974	0.056118
C	-2.378459	1.022154	0.095476
C	-1.022738	1.295320	-0.015646
F	0.261764	-2.073266	-0.380396
F	-2.361191	-2.585323	-0.137045
F	-4.152998	-0.540475	0.172954
F	-3.244574	2.025901	0.264367
F	-0.645692	2.573984	0.081440

2TS5b (gv_az7b_ts3.log)

N	-2.495200	1.672841	-1.268934
N	-3.535191	1.522962	-0.878304
N	-1.345700	0.428228	0.202770

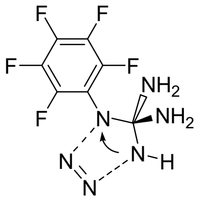

C	-0.040539	0.135551	0.095768
N	-3.614753	0.277130	0.588256
C	-2.423295	-0.510099	0.304843
H	-4.415298	-0.242550	0.213460
N	-2.263008	-1.251684	1.581246
N	-2.696871	-1.481479	-0.740069
H	-1.990977	-0.605372	2.318418
H	-3.142070	-1.690668	1.837844
H	-2.829522	-1.013741	-1.630593
H	-1.932375	-2.137036	-0.836400
C	0.637405	-1.101744	-0.106225
C	2.015956	-1.195857	-0.169689
C	2.811229	-0.063758	-0.063506
C	2.206306	1.175252	0.123860
C	0.834989	1.263638	0.213717
F	-0.030242	-2.261229	-0.237540
F	2.593563	-2.388476	-0.343731
F	4.136559	-0.162778	-0.134606
F	2.966139	2.267938	0.232904
F	0.302092	2.466266	0.406297

3TS5b (gv_az7b_ts6.log)

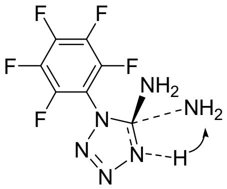

N	1.808196	-0.821070	-1.191680
N	3.072932	-0.669053	-1.102296
N	1.199559	-0.198594	-0.188229
C	-0.192092	-0.082106	-0.108836
C	3.349697	0.143822	0.083092
N	2.071288	0.437059	0.706579
N	4.251074	1.243466	-0.100709
N	3.890965	-0.757016	1.236162
H	2.752526	-0.426609	1.622983
H	3.980346	-1.729904	0.946451
H	4.746059	-0.422039	1.680791
H	4.883007	1.054999	-0.874932
H	3.710310	2.072102	-0.320461
C	-0.817913	1.167171	-0.068805
C	-2.195488	1.274924	0.041415
C	-2.979913	0.130123	0.087381
C	-2.380430	-1.120027	0.037232
C	-0.999903	-1.219739	-0.046024
F	-0.099161	2.284151	-0.164429
F	-2.775119	2.476953	0.071462
F	-4.305469	0.232602	0.176717
F	-3.132268	-2.221297	0.096980
F	-0.447325	-2.434310	-0.039213

1M5b (gv_az7b_m2.log)

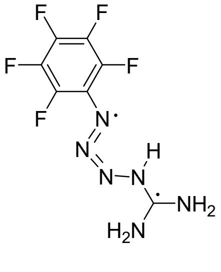

N	-1.460688	-1.162960	-0.325278
N	-2.744662	-1.172937	-0.304133
N	-0.866589	-0.044135	-0.022841
C	0.518506	-0.070467	-0.030774
N	-3.274232	0.100320	0.078792
C	-4.565000	0.275856	0.101138
H	-2.546255	0.800659	0.261884
N	-5.375734	-0.698480	-0.361520
N	-5.126848	1.416411	0.599207
H	-6.305124	-0.772112	0.020323
H	-4.894848	-1.560743	-0.594555

H	-4.495081	2.165126	0.841803
H	-5.985802	1.723309	0.165866
C	1.167762	1.175375	-0.086721
C	2.543853	1.310607	-0.062611
C	3.347989	0.184164	0.022307
C	2.747574	-1.062999	0.090278
C	1.365805	-1.190051	0.068230
F	0.430564	2.297521	-0.172744
F	3.103371	2.526522	-0.130152
F	4.682029	0.300251	0.042121
F	3.513463	-2.157543	0.192106
F	0.871622	-2.428828	0.184670

2M5b (gv_az7b_m1.log)

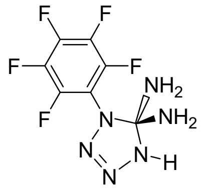

N	-1.985799	-0.603327	-1.527613
N	-3.216358	-0.712143	-1.360762
N	-1.448999	0.155210	-0.484078
C	-0.063363	0.060437	-0.274318
N	-3.611646	-0.031298	-0.226836
C	-2.460962	0.274177	0.628038
H	-4.395262	-0.473681	0.236156
N	-2.607555	1.588021	1.188425
N	-2.300767	-0.660407	1.707460
H	-1.763815	1.892610	1.663053
H	-2.822290	2.251054	0.452813
H	-1.819639	-1.513426	1.456576
H	-1.950757	-0.262269	2.567276
C	0.726718	1.212647	-0.281035
C	2.102309	1.156148	-0.110187
C	2.724788	-0.072525	0.058896
C	1.967841	-1.236399	0.067726
C	0.592702	-1.159636	-0.087258
F	0.163937	2.409510	-0.467798
F	2.830876	2.273723	-0.121809
F	4.044366	-0.134922	0.219539
F	2.565073	-2.415753	0.245960
F	-0.114316	-2.294310	-0.038472

2P5b (gv_az7b_m6.log)

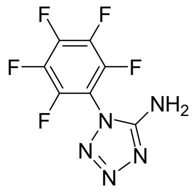

N	-2.142014	-0.616594	1.225408
N	-3.412362	-0.509023	0.969137
N	-1.504857	-0.106852	0.176622
C	-0.097362	-0.045875	0.086746
C	-3.510284	0.078837	-0.258448
N	-2.326993	0.349097	-0.788506
N	-4.711071	0.298191	-0.878209
H	-5.500789	0.343904	-0.251458
H	-4.706044	1.011044	-1.592003
C	0.561601	1.180881	0.116277
C	1.943467	1.246303	0.024822
C	2.681329	0.074556	-0.084745
C	2.039654	-1.156449	-0.110000
C	0.655615	-1.211564	-0.031085
F	-0.131445	2.308925	0.245220
F	2.566613	2.423675	0.055867
F	4.006944	0.132524	-0.165264
F	2.753109	-2.275661	-0.223511
F	0.054089	-2.395998	-0.084435

3P5b (gv_az7b_m3.log)

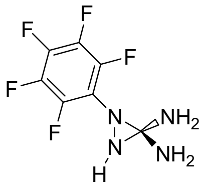

N	-1.720007	0.389850	-0.693784
C	-0.364231	0.212696	-0.352592
N	-2.442672	1.434790	0.128989
C	-2.783554	0.039560	0.258627
H	-3.058264	1.909862	-0.531527
N	-2.446388	-0.628623	1.464280
N	-4.015957	-0.435852	-0.257871
H	-1.723670	-0.126542	1.967178
H	-3.266151	-0.688703	2.058955
H	-4.158521	-0.114179	-1.208790
H	-4.019895	-1.451504	-0.265043
C	0.168183	-1.078996	-0.315676
C	1.515903	-1.308900	-0.089470
C	2.377915	-0.238366	0.099593
C	1.880174	1.054561	0.042505
C	0.529445	1.274207	-0.190627
F	-0.630213	-2.130389	-0.539155
F	1.987762	-2.558336	-0.053896
F	3.676784	-0.451232	0.324766
F	2.707565	2.091788	0.201420
F	0.109065	2.539427	-0.291605

1TS6a (gv2_az7a_ts1a.log)

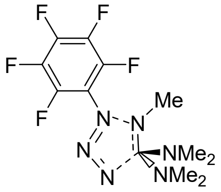

N	0.818541	-0.314933	1.758039
N	1.940093	-0.674622	1.659436
N	0.209963	0.548225	0.951626
C	-1.111855	0.273035	0.574951
C	2.448214	-0.036278	-0.249159
N	1.437140	0.859950	-0.424789
N	2.344270	-1.218397	-0.992618
N	3.806276	0.331719	-0.032214
C	1.746259	2.277116	-0.252381
C	3.069341	-2.400641	-0.545775
C	1.154857	-1.517728	-1.769397
C	4.245059	1.062782	1.141697
C	4.632899	0.554850	-1.211568
C	-1.908896	1.340395	0.142268
C	-3.213748	1.159511	-0.284891
C	-3.778105	-0.107679	-0.264742
C	-3.022192	-1.184743	0.175567
C	-1.707362	-0.996520	0.570217
F	-1.420042	2.583975	0.149760
F	-3.938048	2.203184	-0.695559
F	-5.035046	-0.289923	-0.667288
F	-3.559451	-2.407014	0.196111
F	-1.018168	-2.071434	0.964725
H	2.572125	2.543473	-0.915728
H	0.875291	2.859077	-0.543417
H	2.015068	2.552722	0.769008
H	4.230614	2.153754	1.011976
H	3.629891	0.804397	1.999775
H	5.277133	0.774645	1.366759
H	4.623294	1.602207	-1.550119
H	5.670231	0.293096	-0.981753
H	4.290100	-0.075961	-2.028949
H	3.438804	-2.954486	-1.413534
H	3.913496	-2.104287	0.069937

H	2.426447	-3.069648	0.039197
H	1.435944	-2.181363	-2.591310
H	0.383052	-2.031039	-1.179880
H	0.734502	-0.603855	-2.178796

2TS6a (gv2_az7a_ts3.log)

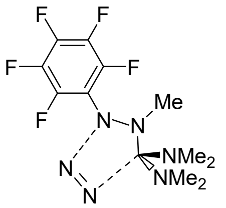

N	0.951829	1.961503	0.089342
N	2.066867	1.692680	0.349848
N	0.281512	0.616299	-0.672208
C	-1.021215	0.268027	-0.353279
C	2.406044	0.046586	0.146660
N	1.311349	-0.309644	-0.749191
N	2.305752	-0.516080	1.443068
N	3.720761	-0.056105	-0.344615
C	1.669309	-0.557577	-2.155159
C	3.181544	0.053714	2.469998
C	1.008393	-0.903991	1.973697
C	4.266595	0.893343	-1.296979
C	4.463287	-1.300847	-0.229540
C	-1.988114	1.277276	-0.162702
C	-3.323299	0.997722	0.070998
C	-3.779739	-0.311033	0.102070
C	-2.869124	-1.327050	-0.121395
C	-1.529023	-1.044383	-0.346202
F	-1.640068	2.568208	-0.240769
F	-4.184032	2.003793	0.258286
F	-5.068530	-0.582464	0.338350
F	-3.278905	-2.601365	-0.118587
F	-0.733697	-2.103434	-0.599313
H	2.498222	-1.259843	-2.200782
H	0.808015	-1.021234	-2.632433
H	1.919147	0.358483	-2.696628
H	4.271632	0.510618	-2.325600
H	3.705483	1.824662	-1.275753
H	5.301908	1.120831	-1.023093
H	4.531433	-1.824125	-1.193497
H	5.485267	-1.101209	0.110276
H	3.979676	-1.957993	0.488025
H	3.407899	-0.717398	3.209663
H	4.110979	0.396862	2.024416
H	2.710798	0.899140	2.985878
H	1.178293	-1.479187	2.885195
H	0.374863	-0.043777	2.234459
H	0.489034	-1.539761	1.265455

3TS6a (gv2_az7a_ts5.log)

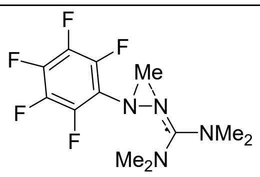

N	0.383629	-0.923847	0.227158
C	-0.855092	-0.358544	0.129654
C	2.563375	-0.061345	-0.003804
N	1.433090	0.002574	0.669123
N	2.669790	-0.488365	-1.302157
N	3.747965	0.363439	0.584765
C	0.915071	-1.037421	2.144668
C	3.790757	-1.296898	-1.757882
C	1.575058	-0.343163	-2.250315
C	3.806165	0.490370	2.033379
C	4.523004	1.388795	-0.119543
C	-1.957965	-1.249731	0.142781

C	-3.268996	-0.835810	0.027456
C	-3.568979	0.515223	-0.101128
C	-2.525300	1.424727	-0.121067
C	-1.207586	1.004480	-0.008881
F	-1.719715	-2.569190	0.262403
F	-4.262445	-1.735419	0.051093
F	-4.841904	0.926772	-0.200182
F	-2.792010	2.731340	-0.266362
F	-0.262097	1.963782	-0.111004
H	1.472470	-0.407122	2.828586
H	-0.138090	-1.048684	2.404497
H	1.334858	-2.026062	2.015637
H	3.146206	1.278439	2.419353
H	3.550185	-0.454254	2.506850
H	4.831738	0.732631	2.313991
H	4.197911	2.396921	0.169015
H	5.581737	1.281626	0.124262
H	4.400249	1.284673	-1.193632
H	4.297509	-0.827979	-2.608542
H	4.508178	-1.428720	-0.952140
H	3.435716	-2.283486	-2.076758
H	2.002237	-0.097522	-3.227608
H	0.977653	-1.253467	-2.338119
H	0.926591	0.476740	-1.953053

4TS6a (gv2_az7a_ts6.log)

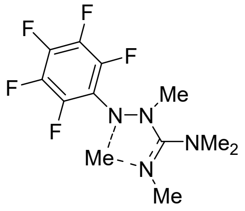

N	0.410612	-0.853056	0.448956
C	-0.845909	-0.319012	0.239632
C	2.627311	-0.214920	0.091275
N	1.418230	0.112372	0.626121
N	2.737028	-1.358139	-0.526002
N	3.646133	0.714990	0.217670
C	1.325352	0.825244	1.901094
C	3.811757	-1.591646	-1.462234
C	0.879759	-1.959502	-1.339521
C	4.989425	0.318408	0.603151
C	3.433352	2.137564	0.000283
C	-1.934288	-1.197233	0.444171
C	-3.251895	-0.827872	0.242673
C	-3.560885	0.456680	-0.182321
C	-2.526315	1.348339	-0.414553
C	-1.207889	0.962377	-0.228036
F	-1.698024	-2.442075	0.882451
F	-4.237777	-1.703493	0.473096
F	-4.833549	0.823770	-0.374302
F	-2.803393	2.585762	-0.848321
F	-0.265470	1.881671	-0.529090
H	2.277152	1.291986	2.135921
H	0.556826	1.597450	1.856645
H	1.069856	0.111639	2.689020
H	5.318982	0.912595	1.463352
H	5.001676	-0.728987	0.895398
H	5.714891	0.465844	-0.205093
H	3.591924	2.722721	0.914750
H	4.134370	2.506013	-0.757391
H	2.420212	2.313040	-0.353283
H	4.193814	-0.675841	-1.927626
H	4.647179	-2.118887	-0.992217

H	3.446660	-2.246677	-2.261028
H	1.550409	-2.045529	-2.187683
H	0.739301	-2.880693	-0.797625
H	-0.042011	-1.479131	-1.648541

TS6a (gv2_az7a_ts4.log)

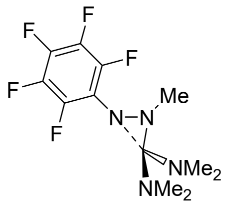

N	-0.762206	0.416754	-0.988151
C	0.551772	0.221824	-0.585152
C	-2.112114	0.199867	0.231794
N	-1.469368	1.389980	-0.119780
N	-1.829549	-0.315608	1.465191
N	-3.260966	-0.276984	-0.385629
C	-2.185335	2.432818	-0.839261
C	-1.814810	-1.759772	1.672191
C	-1.134457	0.457198	2.487573
C	-3.395318	-0.443881	-1.826765
C	-4.379119	-0.797035	0.385023
C	1.102724	-1.067855	-0.663991
C	2.429940	-1.337416	-0.375742
C	3.276853	-0.311864	0.017290
C	2.774273	0.978990	0.101485
C	1.446898	1.236222	-0.199911
F	0.312751	-2.100127	-1.014118
F	2.895383	-2.591968	-0.452958
F	4.561568	-0.561552	0.303221
F	3.589319	1.983074	0.453700
F	1.040422	2.516443	-0.157921
H	-2.883130	2.910492	-0.147634
H	-1.452820	3.173975	-1.157466
H	-2.734002	2.079043	-1.715558
H	-4.214906	0.169198	-2.217212
H	-2.466184	-0.176591	-2.316308
H	-3.619089	-1.493125	-2.054131
H	-5.309223	-0.387868	-0.021064
H	-4.449647	-1.891441	0.337840
H	-4.300621	-0.495216	1.426307
H	-2.494093	-2.052586	2.478472
H	-2.101147	-2.273844	0.759001
H	-0.805211	-2.084827	1.940199
H	-1.590209	0.247800	3.459727
H	-0.073891	0.191455	2.541542
H	-1.214056	1.517135	2.270358

1M6a (gv2_az7a_m1a.log)

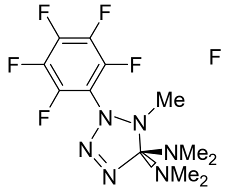

N	0.799131	0.136915	-1.622763
N	1.995420	0.421794	-1.474467
N	0.266978	-0.446219	-0.488760
C	-1.114049	-0.197073	-0.269609
C	2.469826	0.104373	-0.078550
N	1.160458	-0.163224	0.608913
N	3.109295	1.246099	0.545887
N	3.415701	-0.978827	-0.173590
C	1.145293	-1.212927	1.619052
C	4.326940	1.701775	-0.137579
C	2.242578	2.389870	0.836951
C	3.134756	-2.108822	-1.043477
C	4.266448	-1.318861	0.957266
C	-1.981169	-1.278239	-0.117729

C	-3.342206	-1.093387	0.082196
C	-3.859991	0.192596	0.126807
C	-3.018249	1.286883	-0.024901
C	-1.659806	1.087155	-0.216532
F	-1.514083	-2.526476	-0.162182
F	-4.153244	-2.143061	0.227476
F	-5.164177	0.378801	0.315108
F	-3.522592	2.521511	0.015997
F	-0.876256	2.154517	-0.355434
H	1.853362	-0.933242	2.397607
H	0.155571	-1.255092	2.076967
H	1.396454	-2.203740	1.230375
H	2.549182	-2.901113	-0.553941
H	2.607428	-1.789062	-1.938681
H	4.083005	-2.551251	-1.361887
H	3.884005	-2.177285	1.527136
H	5.266405	-1.585266	0.597537
H	4.356094	-0.470259	1.630389
H	4.850316	2.397959	0.520559
H	4.984630	0.865547	-0.355000
H	4.104311	2.217017	-1.080667
H	2.818032	3.102119	1.430684
H	1.896236	2.902438	-0.071566
H	1.377040	2.077774	1.411890

2M6a (gv2_az7a_m3.log)

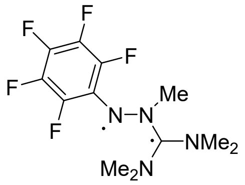

N	0.396751	0.891586	-0.353190
C	-0.861898	0.383116	-0.228481
C	2.534757	0.008905	0.040572
N	1.406469	-0.030013	-0.657355
N	2.543815	0.274579	1.374434
N	3.740348	-0.206050	-0.579476
C	1.315049	-0.743752	-1.937117
C	3.484663	1.240235	1.932771
C	1.395913	-0.022652	2.223702
C	4.084545	0.435788	-1.839865
C	4.794903	-0.982774	0.053840
C	-1.927798	1.322920	-0.263052
C	-3.253621	0.975634	-0.117816
C	-3.627140	-0.351984	0.070893
C	-2.629163	-1.305637	0.123740
C	-1.295482	-0.945223	-0.010221
F	-1.635449	2.622205	-0.451154
F	-4.202710	1.922783	-0.174491
F	-4.921898	-0.692056	0.198742
F	-2.947484	-2.594958	0.328875
F	-0.382372	-1.951771	0.125797
H	2.068394	-1.527022	-1.985752
H	0.331108	-1.198954	-2.006740
H	1.433620	-0.048677	-2.770230
H	4.194119	-0.287994	-2.654463
H	3.320944	1.160676	-2.111355
H	5.035197	0.968306	-1.729970
H	5.162564	-1.742179	-0.643405
H	5.644310	-0.356040	0.348752
H	4.405592	-1.482566	0.937591
H	3.963674	0.834143	2.827422
H	4.251818	1.488828	1.204054

H	2.958957	2.161029	2.206817
H	1.768554	-0.256310	3.224032
H	0.705580	0.821399	2.278726
H	0.864730	-0.888636	1.839912

1P6a (gv2_az7a_m5.log)

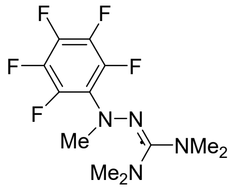

N	0.470743	-0.394750	0.735109
C	-0.895498	-0.181931	0.396395
C	2.531245	0.167176	-0.178144
N	1.279121	0.483149	-0.070822
N	3.131640	-1.035067	0.150319
N	3.396747	1.150251	-0.657313
C	0.742048	-0.264981	2.173977
C	4.421748	-1.098113	0.815699
C	2.451452	-2.305390	-0.035686
C	2.898069	2.518035	-0.726298
C	4.280136	0.805839	-1.768256
C	-1.649097	-1.248627	-0.091848
C	-2.981271	-1.106143	-0.458264
C	-3.594651	0.131111	-0.348416
C	-2.873744	1.214637	0.133739
C	-1.546987	1.051502	0.502548
F	-1.095358	-2.459566	-0.228554
F	-3.671901	-2.153227	-0.922662
F	-4.873951	0.280233	-0.698442
F	-3.468878	2.406777	0.250778
F	-0.900405	2.117811	0.984700
H	0.663342	0.770231	2.526858
H	0.034266	-0.889253	2.721325
H	1.747594	-0.631759	2.379268
H	2.223246	2.675304	-1.576460
H	2.351380	2.758127	0.181987
H	3.752883	3.190946	-0.827907
H	3.783506	0.961093	-2.736300
H	5.172975	1.434451	-1.738219
H	4.586494	-0.235010	-1.705376
H	5.153364	-1.660001	0.222673
H	4.804326	-0.093686	0.979351
H	4.328232	-1.599411	1.787107
H	3.170858	-3.030394	-0.430984
H	2.036199	-2.705047	0.896138
H	1.637329	-2.197622	-0.745571

2P6a (gv2_az7a_m6.log)

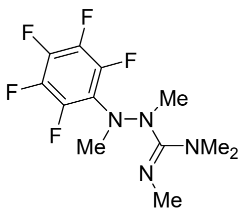

N	-0.454610	-0.866916	0.523854
C	0.879728	-0.396803	0.303814
C	-2.762918	-0.226216	-0.026670
N	-1.374226	-0.054593	-0.290502
N	-3.210289	-1.275616	0.537900
N	-3.516318	0.864088	-0.471632
C	-1.085917	-0.285049	-1.714755
C	-4.560954	-1.329248	1.059842
C	-0.769277	-0.857723	1.964824
C	-4.703138	0.645654	-1.283280
C	-2.968537	2.208758	-0.552957
C	1.843301	-1.305783	-0.137556
C	3.164488	-0.932725	-0.352808
C	3.554465	0.379089	-0.136190

C	2.621810	1.308267	0.303088
C	1.310060	0.916757	0.524575
F	1.514465	-2.576458	-0.374757
F	4.058705	-1.829822	-0.777425
F	4.819137	0.747267	-0.343655
F	2.998413	2.571571	0.525752
F	0.457024	1.844908	0.976506
H	-1.819157	0.257309	-2.307946
H	-0.098589	0.096761	-1.974199
H	-1.137573	-1.352307	-1.958051
H	-4.538444	1.010653	-2.306371
H	-4.937504	-0.413526	-1.338475
H	-5.571326	1.173641	-0.875505
H	-2.607963	2.456764	-1.561476
H	-3.752777	2.927420	-0.295270
H	-2.143010	2.322551	0.142887
H	-5.009015	-0.342534	1.229143
H	-5.221688	-1.893331	0.392544
H	-4.546323	-1.871066	2.009541
H	-0.979222	0.149299	2.344963
H	-1.620931	-1.502793	2.148191
H	0.103272	-1.254647	2.483334

3P6a (gv2_az7a_m4.log)

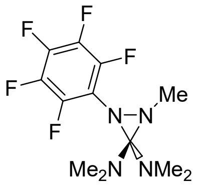

N	-0.848648	0.507523	-0.731729
C	0.498877	0.267389	-0.421559
C	-1.958043	0.076967	0.187629
N	-1.536161	1.467279	0.171269
N	-1.791529	-0.554668	1.440580
N	-3.082183	-0.436327	-0.512511
C	-2.329672	2.445355	-0.564007
C	-1.809089	-2.009764	1.512752
C	-1.049441	0.075697	2.522519
C	-3.076705	-0.641411	-1.949921
C	-4.389570	-0.185356	0.071982
C	1.021661	-1.022847	-0.536528
C	2.367199	-1.297177	-0.352527
C	3.247431	-0.267301	-0.056750
C	2.764235	1.029776	0.034950
C	1.414845	1.289914	-0.152563
F	0.201809	-2.039793	-0.851958
F	2.818044	-2.551475	-0.459438
F	4.547083	-0.519467	0.125226
F	3.607694	2.033653	0.296230
F	1.013170	2.566426	-0.101417
H	-3.044927	2.883132	0.135273
H	-1.660276	3.233847	-0.909296
H	-2.864377	2.030439	-1.420014
H	-3.372169	0.245662	-2.528717
H	-2.089437	-0.946796	-2.283424
H	-3.786566	-1.440562	-2.184804
H	-4.856935	0.735890	-0.306196
H	-5.062597	-1.016833	-0.156840
H	-4.299483	-0.109545	1.154534
H	-2.309933	-2.320873	2.434326
H	-2.355777	-2.415742	0.666179
H	-0.800020	-2.440148	1.516900
H	-1.451143	-0.285804	3.473313

H	0.022444	-0.165143	2.502056
H	-1.159724	1.154298	2.479808

TS6b (gv2_az7b_ts2.log)

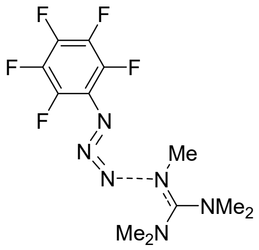

N	0.507112	-1.873612	-0.041046
N	1.602060	-2.382316	-0.102669
N	0.176308	-0.620427	-0.174617
C	-1.183874	-0.351150	-0.097484
N	2.843351	-1.212273	-0.526976
C	2.920712	-0.034718	0.049374
C	3.374617	-1.535222	-1.831251
N	2.853160	0.053379	1.406510
N	3.152847	1.115558	-0.654048
C	2.136212	1.145813	2.057015
C	3.092290	-1.113212	2.247306
C	2.486603	1.363100	-1.926800
C	3.989801	2.191303	-0.144125
C	-1.542673	1.009126	-0.085865
C	-2.852902	1.447789	-0.034458
C	-3.890623	0.529291	0.009943
C	-3.583227	-0.821735	-0.002755
C	-2.265050	-1.252115	-0.057947
F	-0.572893	1.945948	-0.125134
F	-3.121706	2.760669	-0.021912
F	-5.164164	0.940427	0.063424
F	-4.573053	-1.723041	0.032416
F	-2.063026	-2.577735	-0.085819
H	4.279143	-0.956168	-2.032396
H	3.630318	-2.596162	-1.835648
H	2.657751	-1.362841	-2.639441
H	3.165945	1.257628	-2.778557
H	1.647437	0.681093	-2.032913
H	2.092587	2.382744	-1.924080
H	4.704027	2.489555	-0.917115
H	3.402973	3.073513	0.133796
H	4.546418	1.850250	0.725606
H	2.792882	1.704055	2.730799
H	1.716322	1.817367	1.314674
H	1.307294	0.736673	2.640449
H	3.570361	-0.782735	3.172732
H	2.161286	-1.630188	2.496801
H	3.743656	-1.812983	1.730723

1TS6b (gv2_az7b_ts1.log)

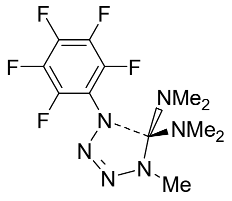

N	0.797731	-1.745512	-1.075695
N	2.020463	-1.996603	-1.134914
N	0.482414	-0.689807	-0.321956
C	-0.864458	-0.366459	-0.256795
N	2.822565	-1.093859	-0.392006
C	2.256250	0.055669	0.207369
C	4.189539	-1.042477	-0.890211
N	2.213309	0.057901	1.595530
N	2.536691	1.250599	-0.407965
C	1.182351	0.861296	2.256284
C	2.407368	-1.220759	2.279676
C	2.326972	1.381552	-1.843235
C	2.856970	2.487814	0.285138
C	-1.297996	0.965599	-0.266277

C	-2.628986	1.328749	-0.144096
C	-3.604982	0.353753	-0.020748
C	-3.219500	-0.979329	-0.005815
C	-1.882752	-1.329431	-0.108696
F	-0.393392	1.959469	-0.405142
F	-2.973722	2.623020	-0.166739
F	-4.895164	0.691060	0.089313
F	-4.148071	-1.931333	0.139998
F	-1.576263	-2.627799	-0.012625
H	4.827142	-0.559421	-0.149663
H	4.524316	-2.069647	-1.028229
H	4.289616	-0.516185	-1.845023
H	3.223528	1.791834	-2.316502
H	2.105410	0.415003	-2.286991
H	1.486879	2.050454	-2.047670
H	3.630993	3.008485	-0.284682
H	1.995225	3.158923	0.370549
H	3.246787	2.271696	1.276510
H	1.544142	1.188215	3.232811
H	0.921429	1.734776	1.670112
H	0.274430	0.265732	2.403273
H	2.519479	-1.013110	3.344557
H	1.550932	-1.890932	2.139433
H	3.302340	-1.720474	1.921730

2TS6b (gv2_az7b_ts3.log)

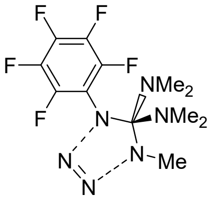

N	1.000836	-0.435673	-2.411495
N	1.802001	-1.192428	-2.190208
N	0.624754	0.546890	-0.477195
C	-0.667890	0.219497	-0.236878
N	2.091641	-1.347832	-0.270409
C	1.831228	0.019457	0.134096
C	3.405889	-1.875858	-0.038389
N	1.634119	-0.002114	1.627272
N	2.857352	1.044370	-0.111686
C	1.143339	1.243501	2.214309
C	1.043938	-1.167817	2.269730
C	2.976449	1.665955	-1.416864
C	4.108052	1.036088	0.625820
C	-1.578998	1.316420	-0.230913
C	-2.946021	1.168167	-0.106869
C	-3.492420	-0.103277	0.011948
C	-2.655410	-1.210501	-0.004798
C	-1.284956	-1.058083	-0.127819
F	-1.105731	2.555594	-0.340998
F	-3.749273	2.236166	-0.088849
F	-4.807989	-0.259500	0.138644
F	-3.187428	-2.433984	0.085897
F	-0.568920	-2.178452	-0.195375
H	3.635958	-1.805047	1.034704
H	3.420732	-2.928893	-0.322981
H	4.208313	-1.349176	-0.564997
H	3.547685	1.059215	-2.137301
H	1.993277	1.869780	-1.831019
H	3.504000	2.616375	-1.297016
H	4.939097	0.582460	0.066238
H	4.398064	2.069442	0.848085
H	3.992488	0.510962	1.570096

H	1.507952	1.288140	3.244811
H	1.535415	2.090967	1.659947
H	0.048072	1.305449	2.242877
H	1.274248	-1.106264	3.336671
H	-0.049600	-1.206434	2.174691
H	1.444267	-2.089174	1.857123

1M6b (gv2_az7b_m2.log)

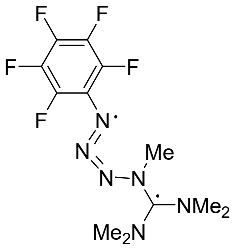

N	0.602490	-1.283422	-1.309527
N	1.818000	-1.533374	-1.579030
N	0.260100	-0.233083	-0.613882
C	-1.109280	-0.125956	-0.403639
N	2.780097	-0.506880	-1.085637
C	2.796949	-0.063530	0.167470
C	3.613789	0.009847	-2.155125
N	2.553710	-0.905255	1.196984
N	3.130793	1.227223	0.434862
C	1.773950	-0.471705	2.354299
C	2.701652	-2.351120	1.058020
C	2.653234	2.316845	-0.412527
C	3.901279	1.608001	1.610582
C	-1.659397	1.162377	-0.307256
C	-2.995296	1.393952	-0.030136
C	-3.856391	0.325688	0.170186
C	-3.351300	-0.963482	0.099276
C	-2.009533	-1.182891	-0.175365
F	-0.865853	2.234446	-0.501753
F	-3.463713	2.648780	0.031745
F	-5.152878	0.536391	0.435509
F	-4.166238	-2.003953	0.322691
F	-1.581938	-2.454846	-0.152353
H	4.499002	0.493104	-1.742219
H	3.917270	-0.838162	-2.770100
H	3.073751	0.715864	-2.790834
H	3.443902	2.715231	-1.055431
H	1.819261	1.963346	-1.013483
H	2.291358	3.124163	0.228485
H	4.713556	2.274006	1.306945
H	3.288441	2.134674	2.349204
H	4.334051	0.725135	2.075180
H	2.358343	-0.550687	3.275223
H	1.440128	0.552710	2.219449
H	0.888688	-1.104552	2.446143
H	3.055658	-2.751087	2.010855
H	1.753036	-2.826692	0.796844
H	3.424879	-2.579025	0.280086

2M6b (gv2_az7b_m1.log)

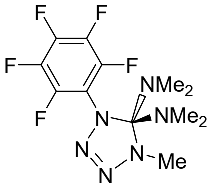

N	0.976715	-1.117852	-1.783724
N	2.185793	-1.425292	-1.697015
N	0.651732	-0.267389	-0.723070
C	-0.707641	-0.104463	-0.433723
N	2.740313	-0.907263	-0.574399
C	1.839961	0.001141	0.154358
C	4.172550	-0.983770	-0.400681
N	1.733904	-0.405005	1.541042
N	2.192926	1.413639	0.095905
C	0.861937	0.387464	2.410137

C	1.574731	-1.833342	1.799091
C	2.347225	1.956081	-1.250378
C	3.260274	1.900394	0.971995
C	-1.316581	1.154727	-0.360367
C	-2.672062	1.300385	-0.105563
C	-3.475521	0.181049	0.049145
C	-2.909994	-1.081832	-0.043662
C	-1.549031	-1.214723	-0.270464
F	-0.605477	2.268204	-0.552855
F	-3.213208	2.520147	-0.037233
F	-4.780837	0.317808	0.281995
F	-3.674759	-2.165250	0.114328
F	-1.043518	-2.449664	-0.303912
H	4.406141	-0.963059	0.663632
H	4.515357	-1.929973	-0.818330
H	4.705803	-0.167560	-0.898066
H	3.296971	1.659743	-1.720623
H	1.530446	1.640678	-1.892586
H	2.327643	3.045683	-1.191311
H	4.262191	1.743367	0.548239
H	3.130197	2.976364	1.114714
H	3.223396	1.414426	1.941598
H	1.205149	0.289546	3.443513
H	0.890943	1.436818	2.131509
H	-0.182273	0.048656	2.375135
H	1.778334	-2.012316	2.856712
H	0.564150	-2.202371	1.581469
H	2.280979	-2.415730	1.212987

1P6b (gv2_az7b_m4.log)

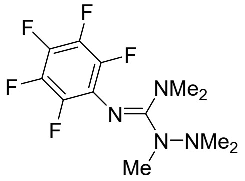

N	0.518999	0.667424	-0.499735
C	-0.805012	0.349959	-0.283411
N	2.719237	0.039107	-0.696578
C	1.515342	-0.003711	-0.002287
C	2.699812	0.633681	-2.035772
N	1.494615	-0.733277	1.153732
N	3.881286	0.235154	0.100885
C	0.601956	-0.391268	2.248137
C	2.353029	-1.884068	1.380742
C	4.173490	1.648170	0.346451
C	5.031744	-0.497308	-0.409139
C	-1.735209	1.359213	0.009689
C	-3.095770	1.116410	0.101918
C	-3.591535	-0.163730	-0.101082
C	-2.705758	-1.188571	-0.396602
C	-1.346913	-0.929740	-0.481414
F	-1.298940	2.610018	0.225043
F	-3.938422	2.114221	0.396848
F	-4.905441	-0.405702	-0.011447
F	-3.168367	-2.428647	-0.604746
F	-0.528875	-1.950678	-0.802420
H	3.653979	0.427582	-2.520054
H	1.906550	0.169829	-2.616131
H	2.521206	1.711842	-2.019116
H	4.523982	2.185323	-0.546927
H	3.279889	2.143948	0.726064
H	4.952307	1.710664	1.108381
H	5.430496	-0.096723	-1.353767

H	5.828854	-0.447235	0.334798
H	4.763148	-1.542061	-0.561394
H	1.158395	-0.446457	3.188318
H	0.233769	0.625077	2.131150
H	-0.254170	-1.071070	2.317108
H	3.177526	-1.651865	2.060819
H	1.752248	-2.690117	1.812317
H	2.762213	-2.234728	0.437433

N_2 (n2.log)

N	0.000000	0.000000	0.545750
N	0.000000	0.000000	-0.545750

NH_3 (nh3.log)

H	0.546978	-0.769411	0.261687
N	-0.000077	-0.000037	-0.112163
H	0.393409	0.858215	0.261593
H	-0.939848	-0.088547	0.261863

$NH(CH_3)_2$ (NHMe2.log)

N	0.000028	0.562208	-0.147784
C	-1.215787	-0.222784	0.020251
C	1.215757	-0.222805	0.020273
H	-0.000224	1.336641	0.506173
H	1.284504	-0.961837	-0.782464
H	2.088366	0.428613	-0.053983
H	1.267317	-0.765876	0.979030
H	-1.283745	-0.962944	-0.781538
H	-1.267875	-0.764972	0.979514
H	-2.088368	0.428452	-0.055386

Table S5. Coordinates of structures with one $CHCl_3$ molecule included explicitly (Å) optimized at B3LYP/6-311+G(2d,p)/SMD(solvent=chloroform) level of theory using Integral=ultrafine option.

AZ3 (az3_kl0.log)

$Ph-N_3$

N	2.205437	-0.982754	-0.000000
N	3.313302	-0.761564	-0.000000
N	1.036389	-1.364793	-0.000000
C	0.000000	-0.390105	-0.000000
C	-1.305246	-0.883462	-0.000000
C	-2.374425	0.003540	0.000000
C	-2.150292	1.378367	0.000000
C	-0.844496	1.861634	0.000000
C	0.235365	0.985393	0.000000
H	-1.467249	-1.954202	-0.000000
H	-3.386812	-0.383093	0.000000
H	-2.985895	2.067698	0.000000
H	-0.659850	2.929577	0.000000
H	1.248472	1.371592	0.000000

GU · $CHCl_3$ (gv_kl6.log)

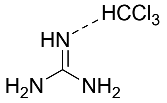

C	2.960567	0.135889	-0.052535
N	1.850337	0.780611	-0.170731
N	2.960754	-1.237933	0.062456
N	4.221268	0.700570	-0.035697
H	2.005374	1.786347	-0.158747
H	4.250628	1.695121	0.139726
H	4.942301	0.187487	0.454353
H	3.770577	-1.721817	-0.301013
H	2.085397	-1.676197	-0.187470
C	-1.229023	0.050425	-0.005092
H	-0.160771	0.274806	-0.074087
Cl	-1.604064	-0.371312	1.693413
Cl	-2.140739	1.503680	-0.514682
Cl	-1.579273	-1.330390	-1.091625

1TS3a · $CHCl_3$ (gv_az3a_ts1_kl6.log)

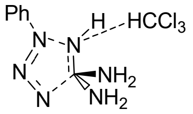

N	-2.729678	1.697743	-0.268538
N	-2.332275	2.754381	0.093577
N	-2.102932	0.735945	-0.834303
C	-2.303942	-0.590931	-0.401030
C	-0.453954	2.653615	0.011947
N	-0.237936	1.492114	-0.643507
N	-0.066419	2.654054	1.376748
N	-0.253993	3.883569	-0.703345
H	-0.203868	1.683724	-1.642753
H	-0.516105	3.393459	1.906506
H	-0.235971	1.759038	1.823929
H	-0.788475	4.637154	-0.280423
H	0.727555	4.148503	-0.665474
C	-1.773592	-1.598925	-1.209748
C	-1.890949	-2.926610	-0.821853
C	-2.556785	-3.257520	0.355510
C	-3.097967	-2.250354	1.152428
C	-2.965694	-0.917785	0.788238
H	-1.269909	-1.330120	-2.129936
H	-1.470383	-3.705591	-1.446348
H	-2.658186	-4.295299	0.649420
H	-3.623368	-2.503912	2.065645
H	-3.388132	-0.135372	1.407038

C	2.334604	-0.481211	0.020775
H	1.443618	0.115022	-0.168665
Cl	3.734538	0.627595	0.121544
Cl	2.101443	-1.353501	1.564816
Cl	2.534344	-1.631325	-1.335827

1M3a · $CHCl_3$ (gv_az3a_m1_kl6.log)

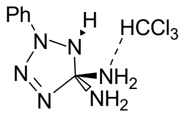

C	4.531378	0.204316	-0.515034
C	3.259470	-0.122285	-0.027407
C	3.012369	-1.397401	0.489002
C	4.037649	-2.337321	0.514340
C	5.302699	-2.023411	0.031544
C	5.538235	-0.748924	-0.482340
N	2.228282	0.824098	-0.053130
N	2.350492	2.066132	-0.541510
N	1.285688	2.720980	-0.419130
C	0.217922	1.839205	0.182953
N	0.973350	0.628333	0.570053
N	-0.774065	1.484110	-0.810238
N	-0.410483	2.451437	1.315431
H	1.110034	0.648715	1.582622
H	-1.110017	2.322582	-1.280839
H	-0.350309	0.888859	-1.517820
H	0.103147	3.265939	1.634746
H	-1.369992	2.712584	1.120220
H	2.027549	-1.652114	0.855933
H	3.836966	-3.323894	0.915398
H	6.096869	-2.759558	0.054526
H	6.519920	-0.489606	-0.861313
H	4.721390	1.191718	-0.912033
C	-3.357438	-0.529694	-0.113894
H	-2.488021	0.108605	-0.257762
Cl	-3.453222	-1.652903	-1.503029
Cl	-4.803447	0.523215	-0.046876
Cl	-3.150631	-1.425980	1.418200

2TS3a · $CHCl_3$ (gv_az3a_ts3_kl6.log)

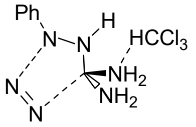

N	-2.542818	-2.366369	0.276335
N	-1.615229	-2.619881	-0.394130
N	-2.219705	-0.784814	1.016310
C	-3.166996	0.134237	0.507408
C	-0.507588	-1.352824	-0.507015
N	-0.903404	-0.600115	0.659228
N	-0.797006	-0.860356	-1.803567
N	0.833834	-1.771270	-0.419050
H	-0.326621	-0.886977	1.446990
H	1.007300	-2.370921	0.381351
H	1.126065	-2.247999	-1.265106
H	-1.716933	-0.438518	-1.863785
H	-0.099308	-0.185101	-2.102784
C	-2.814426	1.380709	-0.024332
C	-3.803696	2.268817	-0.438196
C	-5.149553	1.934053	-0.337140
C	-5.500709	0.696046	0.200225
C	-4.526050	-0.193881	0.627346
H	-1.770962	1.658530	-0.087756
H	-3.512839	3.233380	-0.839331
H	-5.914933	2.628649	-0.661629
H	-6.545598	0.422715	0.294783
H	-4.806364	-1.146638	1.058233

C	3.500965	0.340993	0.062889
H	2.577926	-0.202242	-0.115303
Cl	4.761595	-0.361885	-0.992721
Cl	3.222350	2.061309	-0.332770
Cl	3.927871	0.142097	1.787923

2M3a · $CHCl_3$ (gv_az3a_m3_kl6.log)

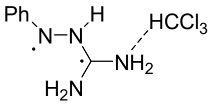

N	-2.749353	-1.486383	0.083936
C	-3.463369	-0.320886	0.039507
C	-0.457234	-1.998571	0.251680
N	-1.500094	-1.331353	0.686903
N	-0.589332	-2.788700	-0.826821
N	0.764197	-1.904694	0.871552
H	-1.421733	-0.839423	1.575280
H	0.746876	-1.500670	1.800231
H	1.321502	-2.750621	0.838734
H	-1.474833	-2.693838	-1.312525
H	0.231269	-3.031828	-1.361853
C	-3.006005	0.966530	0.424463
C	-3.843736	2.077327	0.355123
C	-5.152274	1.972597	-0.103460
C	-5.614400	0.711305	-0.502572
C	-4.801784	-0.403493	-0.433384
H	-1.982980	1.106481	0.753115
H	-3.455130	3.044978	0.657684
H	-5.795370	2.842631	-0.156937
H	-6.629957	0.601631	-0.869804
H	-5.176739	-1.375325	-0.736691
C	3.246224	0.406613	-0.102902
H	2.368311	-0.226474	-0.047338
Cl	4.676148	-0.625092	0.199247
Cl	3.311217	1.131114	-1.732649
Cl	3.091830	1.666439	1.155778

3TS3a · $CHCl_3$ (gv_az3a_ts6_kl6.log)

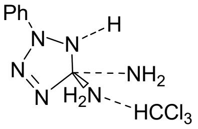

N	-2.425217	-2.046652	-0.464940
N	-1.287501	-2.645901	-0.448142
N	-2.301240	-0.794185	-0.074743
C	-3.372416	0.114251	-0.052960
C	-0.282474	-1.691389	0.021233
N	-0.994838	-0.438591	0.257133
N	0.919415	-1.586147	-0.764085
N	0.099658	-1.955932	1.479134
H	-0.557553	-0.872080	1.518023
H	-0.391687	-2.760110	1.869023
H	1.098267	-2.011811	1.684637
H	1.314190	-2.508373	-0.941284
H	0.682903	-1.182627	-1.666361
C	-3.131141	1.444744	0.297308
C	-4.189036	2.347809	0.326683
C	-5.480747	1.939270	0.014954
C	-5.710903	0.607649	-0.330298
C	-4.670201	-0.308177	-0.365424
H	-2.125919	1.761419	0.536573
H	-3.994633	3.379318	0.596499
H	-6.300748	2.646672	0.040527
H	-6.713258	0.276030	-0.575124
H	-4.853369	-1.339519	-0.632517
C	3.486774	0.480865	-0.112100
H	2.587765	-0.102928	-0.296292

Cl	4.881572	-0.639719	-0.179405
Cl	3.613091	1.733798	-1.378623
Cl	3.345511	1.222140	1.509758

2P3a · $CHCl_3$ (gv_az3a_m6_kl6.log)

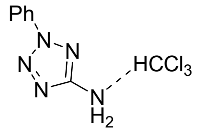

N	-1.921570	1.860368	-0.560219
N	-0.729614	2.261598	-0.188712
N	-2.196747	0.782951	0.148048
C	-3.415184	0.055245	0.022538
C	-0.317899	1.380717	0.757292
N	-1.218062	0.437738	0.994800
N	0.922675	1.434683	1.344347
H	1.333803	2.360246	1.340380
H	0.973710	0.994647	2.254903
C	-3.530472	-1.189227	0.635280
C	-4.720812	-1.895655	0.508077
C	-5.779839	-1.368662	-0.224090
C	-5.647164	-0.122439	-0.831453
C	-4.467478	0.599836	-0.710846
H	-2.701501	-1.594872	1.197813
H	-4.815235	-2.864675	0.982975
H	-6.704552	-1.924551	-0.320647
H	-6.468251	0.296297	-1.400571
H	-4.362800	1.570481	-1.174945
C	3.513624	-0.315139	-0.128075
H	2.625261	0.199226	0.224509
Cl	4.744979	0.921816	-0.515646
Cl	4.079478	-1.388375	1.186630
Cl	3.076375	-1.259689	-1.579489

4TS3a · $CHCl_3$ (gv_az3a_ts5_kl6.log)

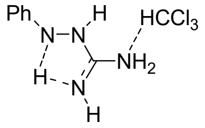

N	-2.776369	-1.481318	0.283669
C	-3.488721	-0.298882	0.148513
C	-0.531590	-1.916279	0.143929
N	-1.497507	-1.319670	0.896364
N	-0.972926	-2.291885	-1.027110
N	0.737172	-2.025785	0.615789
H	-1.451900	-1.452769	1.900907
H	0.872640	-1.889467	1.609426
H	1.318767	-2.744616	0.208164
H	-2.140336	-2.032421	-0.834301
H	-0.363152	-2.683771	-1.729510
C	-3.106590	0.953074	0.669992
C	-3.915030	2.073629	0.497638
C	-5.119826	1.994336	-0.193405
C	-5.508361	0.757384	-0.715009
C	-4.716441	-0.366399	-0.547710
H	-2.171738	1.044416	1.208118
H	-3.591995	3.023496	0.911618
H	-5.744252	2.870236	-0.322319
H	-6.443920	0.669614	-1.257579
H	-5.033239	-1.323641	-0.948211
C	3.264061	0.446939	-0.107491
H	2.251385	0.063729	-0.104631
Cl	3.289183	1.954381	-1.060322
Cl	3.737653	0.750848	1.589871
Cl	4.314349	-0.797995	-0.847278

2P3a · $CHCl_3$ (gv_az3a_m5_kl6.log)

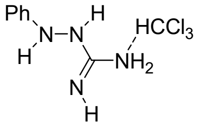

N	2.709839	1.550649	0.625812
C	3.250479	0.290788	0.290631
C	0.519310	2.142629	-0.137056
N	1.331405	1.613539	0.861332
N	1.002456	2.406887	-1.293435
N	-0.810726	2.260742	0.250882
H	1.116547	1.924402	1.800815
H	-0.984223	2.290540	1.248863
H	-1.328418	2.974011	-0.246118
H	2.992643	2.276342	-0.026751
H	0.277560	2.727764	-1.931351
C	2.641710	-0.908358	0.667624
C	3.259845	-2.121982	0.380988
C	4.484780	-2.162717	-0.277368
C	5.089431	-0.964457	-0.653637
C	4.481506	0.251851	-0.375391
H	1.687598	-0.888247	1.177396
H	2.772735	-3.044555	0.676657
H	4.960202	-3.110921	-0.497496
H	6.040187	-0.975563	-1.174639
H	4.955962	1.180210	-0.675975
C	-2.995504	-0.382182	-0.084935
H	-2.170050	0.318045	-0.157740
Cl	-4.513249	0.565666	-0.128477
Cl	-2.905389	-1.507887	-1.466686
Cl	-2.836708	-1.255907	1.467460

5TS3a · $CHCl_3$ (gv_az3a_ts4_kl6.log)

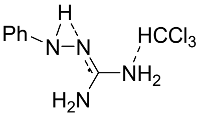

N	2.734432	1.492842	0.073214
C	3.460427	0.304450	0.066451
C	0.425904	2.006179	0.267690
N	1.404839	1.308814	0.835196
N	0.575298	2.616623	-0.911642
N	-0.789099	2.044685	0.891229
H	2.329999	1.854661	1.276502
H	-0.779475	1.738927	1.855818
H	-1.370023	2.855123	0.719304
H	1.476955	2.480554	-1.355885
H	-0.221855	2.914719	-1.453069
C	2.953383	-0.971152	0.376345
C	3.769404	-2.096599	0.292381
C	5.099031	-1.994146	-0.103377
C	5.609737	-0.731852	-0.417400
C	4.812037	0.397175	-0.332138
H	1.920277	-1.071833	0.679574
H	3.352665	-3.068222	0.537156
H	5.726601	-2.874983	-0.168051
H	6.643896	-0.628635	-0.728699
H	5.215968	1.374129	-0.574982
C	-3.215115	-0.427907	-0.045913
H	-2.333601	0.165413	0.167000
Cl	-2.806905	-1.587212	-1.339888
Cl	-3.682945	-1.277252	1.454655
Cl	-4.505947	0.694024	-0.573289

1P3a · $CHCl_3$ (gv_az3a_m4_kl6.log)

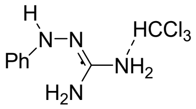

N	-2.562978	1.450535	-0.035719
C	-3.360412	0.297167	-0.045628
C	-0.381433	2.016208	-0.300488
N	-1.382475	1.398047	-0.840576
N	-0.418790	2.737676	0.862981
N	0.850270	1.963962	-0.938346
H	-3.107274	2.277772	-0.264279
H	0.772570	1.642156	-1.896266
H	1.390813	2.819113	-0.876096
H	-1.221825	2.530425	1.445069
H	0.454578	2.828623	1.364449
C	-2.815411	-0.978013	-0.233862
C	-3.633322	-2.101837	-0.164497
C	-4.995638	-1.983073	0.093715
C	-5.538972	-0.711799	0.277774
C	-4.735635	0.416976	0.204961
H	-1.757772	-1.080937	-0.435016
H	-3.195037	-3.082432	-0.314589
H	-5.625151	-2.863101	0.147414
H	-6.599610	-0.597063	0.472102
H	-5.167579	1.403274	0.342581
C	3.123453	-0.407039	0.027199
H	2.317908	0.278721	-0.218967
Cl	4.528329	0.572958	0.547356
Cl	2.568448	-1.475264	1.347472
Cl	3.512659	-1.350614	-1.439523
